# Cation–polymer interactions drive water expulsion and deswelling in n-type ladder organic mixed conductors

**DOI:** 10.1038/s41563-025-02478-2

**Published:** 2026-02-11

**Authors:** Tom P. A. van der Pol, Dongxun Lyu, Zoé Truyens, Vincent Lemaur, Demetra Tsokkou, Arianna Magni, Chiara Musumeci, Han-Yan Wu, Junpeng Ji, David Cornil, Chi-Yuan Yang, Scott T. Keene, Gabriele D’Avino, Alberto Salleo, Natalie Banerji, Clare Grey, David Beljonne, Simone Fabiano

**Affiliations:** 1 Laboratory of Organic Electronics, Department of Science and Technology, Linköping University, Norrköping, Sweden; 2 Yusuf Hamied Department of Chemistry, University of Cambridge, Cambridge, UK; 3 Laboratory for Chemistry of Novel Materials, Materials Research Institute, University of Mons, Mons, Belgium; 4 Department of Chemistry, Biochemistry and Pharmaceutical Sciences, University of Bern, Bern, Switzerland; 5 Department of Materials Science and Engineering, Stanford University, Stanford, CA USA; 6 Department of Engineering, Electrical Engineering Division, University of Cambridge, Cambridge, UK; 7 Department of Materials Science and NanoEngineering, Rice University, Houston, TX USA; 8 Department of Molecular Sciences and Nanosystems, Ca’ Foscari University of Venice, Venice, Italy; 9 Wallenberg Initiative Materials Science for Sustainability, Department ofScience and Technology, Linköping University, Norrköping, Sweden

**Keywords:** Polymers, Electronic devices

## Abstract

Controlling ion–polymer interactions in organic mixed ionic-electronic conductors is crucial for optimizing device performance in applications ranging from bioelectronics and energy storage to photonics. Achieving this requires a molecular-level understanding of how ion uptake, solvation and polymer structure evolve during electrochemical doping. Here using a multimodal operando approach, we uncover an unexpected response in the prototypical n-type ladder polymer poly(benzimidazobenzophenanthroline) (BBL) on doping with protic cations such as ammonium. At high doping levels, strong ion–polymer interactions (primarily hydrogen bonding) between cations and the BBL backbone promote charge localization and disrupt ion hydration, leading to a pronounced reduction in mass and thickness. Operando ^2^H NMR identifies water expulsion, rather than ion removal, as the origin of this deswelling. Our combined experimental and modelling results reveal a previously unobserved regime of ion–polymer coupling in organic mixed ionic-electronic conductors, establishing a framework for material design and applications that span (bio-)electronics to photonics.

## Abstract (web summary)

A molecular-level understanding of structural evolution upon electrochemical doping in organic mixed ionic-electronic conductors is required. A pronounced reduction in mass and thickness is observed in an n-type ladder polymer upon doping with protic cations owing to water expulsion.

## Main

Organic mixed ionic-electronic conductors (OMIECs) enable key applications in bioelectronics^1^, optoelectronics^2,3^, energy harvesting and/or storage^4–6^ and neuromorphic computing^7,8^ due to their ability to simultaneously transport ionic and electronic charges^9^. The functional properties of OMIECs are governed by ion–polymer interactions, which dictate swelling, charge transport and material or device stability^10–13^. A molecular-level understanding of these interactions is crucial, as effective material or device optimization relies on comprehensive structure–property relationships. However, the mechanisms governing counterion uptake, charge mobility and morphology evolution during electrochemical doping remain poorly understood.

A defining characteristic of OMIECs is their ability to uptake solvated ions, which influences their electronic, electrochemical and mechanical properties. Swelling is typically considered integral to OMIEC functionality, as counterion uptake expands the polymer network, facilitating charge compensation and enhancing volumetric capacitance^14–16^. Yet, the complexity of electrochemical doping is not fully described by swelling alone. Beyond simple volumetric expansion, solvated counterions dictate charge transport^17,18^, hydration dynamics or levels^19^ and morphology evolution^20^, ultimately shaping the material’s performance in devices such as actuators^21^, electrochemical transistors^22^, batteries^23^ and metasurfaces^24,25^.

Even though the mechanisms governing ion uptake in OMIECs have been extensively studied, comparatively little is known about how ion–polymer interactions (and their impact on device operation) evolve during electrochemical doping. In particular, decoupling ion uptake and solvation shell movement has proved difficult, and ion injection together with water influx is the generally accepted convention. To enable directed optimization for a range of applications, a holistic understanding of ion–polymer interactions governing material function during electrochemical doping is required, which includes the role of water.

**Fig. 1:**
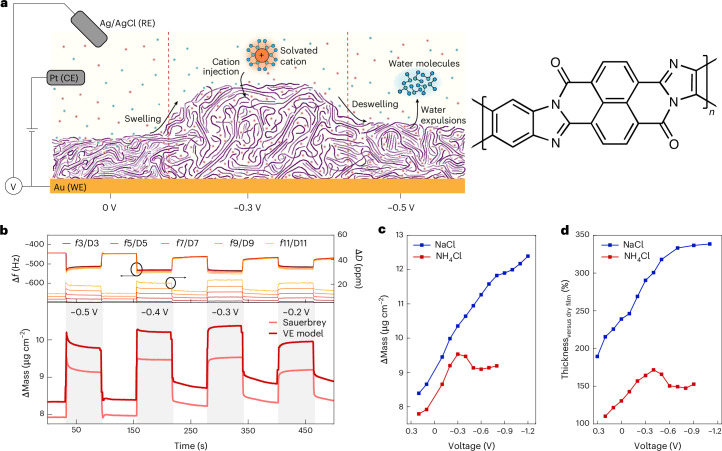
Decreasing mass and thickness with extensive doping of BBL. **a**, Schematic illustration of deswelling on cation injection. CE, counter electrode; RE, reference electrode; WE, working electrode. **b**, The measured Δ*f* (left axis) and Δ*D* (right axis) for the third to eleventh overtones from EQCM-D measurements on BBL submerged in 0.1 M NH_4_Cl. The applied bias is decreased stepwise from −0.5 V to −0.2 V versus Ag/AgCl with 0 V applied between steps. Bottom panel includes the Δmass from the Sauerbrey equation (light red) and viscoelastic (VE) modelling (dark red) relative to the dry mass of the BBL film. While the VE model provides higher mass values, both methods show identical trends. The intermittent 0-V steps already show considerable Δmass due to the swelling on initial doping cycles. **c**, Summary of the Δmass from EQCM-D (Supplementary Figs. 2 and 3) at each voltage step (from the Sauerbrey equation) for 0.1 M NaCl (blue) and 0.1 M NH_4_Cl (red). Error bars denoting standard deviation (<5 ng cm^−2^, obtained from the final 10 datapoints in each voltage step) are smaller than the symbols. **d**, The normalized thickness from EC-AFM of a BBL film submerged in 0.1 M NaCl (blue) and 0.1 M NH_4_Cl (red). Values are normalized to the dry film thickness. Error bars represent propagated standard deviations of the mean and are smaller than the symbols. Underlying data are provided in Supplementary Figs. 6–9. Source data

Here we leverage a multimodal operando characterization approach to reveal how strong cation–polymer interactions influence charge localization, hydration dynamics and morphology evolution in OMIECs. Using poly(benzimidazobenzophenanthroline) (BBL)—a ladder-type, side-chain-free, electron-transporting OMIEC with highly reversible doping behaviour^8,26^—we demonstrate that at high doping levels, protic cations enhance hydrogen bonding, promoting charge localization and disrupting ion hydration, ultimately leading to mass and thickness reduction. Unlike conventional mass loss due to ion ejection^27–30^, we show that this behaviour is driven by water expulsion rather than ion removal, as schematically depicted in Fig. 1a. This molecular-scale structural transformation challenges existing models of ion uptake and charge compensation in OMIECs. Furthermore, deswelling at high doping density is potentially beneficial for long-term electrochemical stability and can enable new applications in tunable metasurfaces. To elucidate this process, we used an array of advanced operando characterization and simulation techniques, providing a comprehensive picture of cation–polymer interactions governing device function during electrochemical doping. Our findings establish a mechanistic understanding of how electrolytes influence ion–polymer interactions, charge localization and morphological response, offering new insights for the application of OMIECs in electrochemical energy devices, metalenses, neuromorphic computing and bioelectronics.

## Effect of counterions on mass and thickness response in BBL

Electrochemical quartz crystal microbalance gravimetry with dissipation monitoring (EQCM-D) was used to track mass changes in BBL films during electrochemical doping in 0.1 M NH_4_Cl. Before measurements, films were precycled to remove effects from initial swelling^31^. The potential was stepped from −0.8 V to +0.2 V versus Ag/AgCl, with 0 V applied between steps, each held for 60 s to reach equilibrium (Supplementary Fig. 1). The −0.8-V bias ensures full doping, consistent with earlier studies^8,26^. As expected, doping caused a negative frequency shift and a positive dissipation shift, indicating mass uptake from solvated cations compensating injected electronic charges (Fig. 1b). Typically, higher doping levels lead to greater counterion influx and mass gain. However, in 0.1 M NH_4_Cl, the mass at −0.4 V exceeded that at −0.5 V (Fig. 1a,b and Supplementary Fig. 2), deviating from the expected doping-mass relationship on ion uptake and differing from the results obtained using 0.1 M NaCl (Supplementary Fig. 3). This anomalous mass response persisted when a +0.2 V versus Ag/AgCl was applied between steps (corresponding to fully dedoped BBL) and when the applied potential was increased rather than decreased (Supplementary Figs. 4 and 5). In addition, the electrochemical response of BBL in 0.1 M NH_4_Cl showed a shift to lower reduction potentials compared with NaCl, as discussed below.

To highlight the unusual mass variation with bias, we summarize the maximum Δmass at the end of each bias step for NaCl and NH_4_Cl in Fig. 1c. In NaCl, Δmass increases continuously, whereas NH_4_Cl shows an initial rise followed by a distinct decrease. Notably, the onset of mass decrease for NH_4_Cl (−0.4 V) and the inflection point for NaCl (−0.8 V) coincide with the bias at the peak conductivity (see previously reported organic electrochemical transistor (OECT) transfer curves^8^ and operando terahertz spectroscopy data below). While the mass changes observed in EQCM-D suggest potential changes in molecular packing, operando grazing-incidence wide-angle X-ray scattering (GIWAXS) of BBL doped in NaCl or NH_4_Cl provides limited insight due to the weakly diffracting nature of BBL, especially after doping (Supplementary Note 1).

To further investigate the doping response of BBL, we conducted electrochemical atomic force microscopy (EC-AFM) on patterned BBL on gold-coated substrates. Thickness changes during doping are summarized in Fig. 1d (also Supplementary Figs. 6–9). As films were precycled, the thickness increase in the dedoped state reflects swelling from the first cycle^31^. The mass decrease observed in EQCM-D during extensive doping with NH_4_^+^ at ~−0.4 V versus Ag/AgCl correlates with a reduction in film thickness. In contrast, BBL in 0.1 M NaCl exhibits a continuous increase in thickness with increasing bias. As more ions are injected at higher biases to compensate for the added charges, a decrease in mass and thickness indicates the expulsion of water molecules from the BBL film.

## Water expulsion at high doping levels

**Fig. 2:**
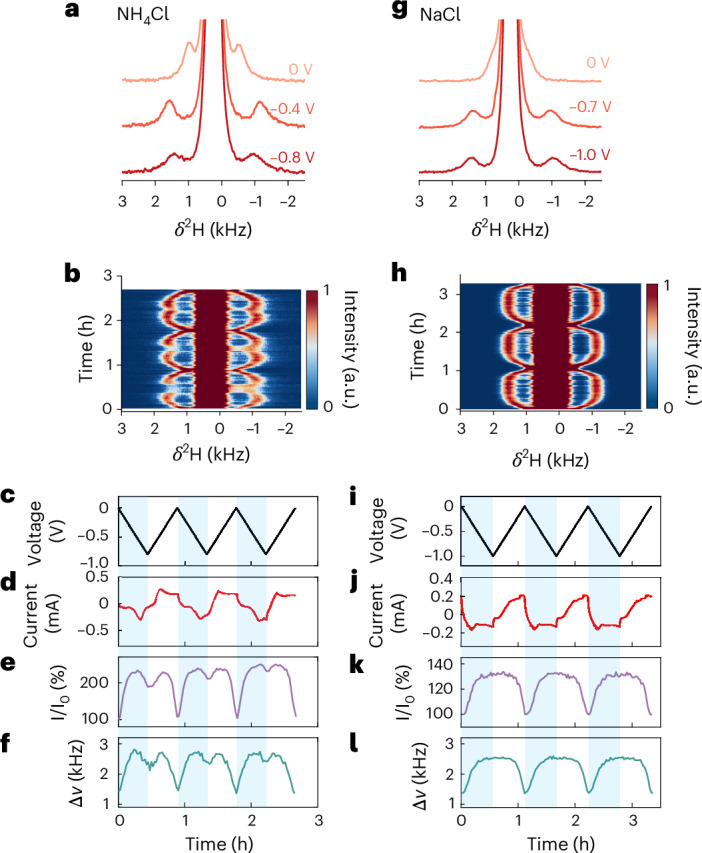
Hydration and dehydration of BBL during doping. **a**–**l**, Operando ^2^H NMR tracking water movement within BBL films cycled in 0.1 M NH_4_Cl (**a**–**f**) and NaCl (**g**–**l**) in D_2_O at a scan rate of 0.5 mV s^−1^ between 0 V and −0.8 V or −1.0 V (versus Ag/AgCl), respectively. **a**,**b**,**g**,**h**, Three selected representative ^2^H NMR spectra (**a**,**g**) extracted from the operando ^2^H NMR spectra (**b**,**h**). **c**,**i**, Voltage profiles (black). **d**,**j**, Charging currents (red). **e**,**k**, Normalized ^2^H intensities for D_2_O inside BBL films (violet). **f**,**l**, The quadrupolar splittings of the anisotropic-water signal (teal), defined as the splitting between two doublet peaks. Source data

The presence of water in structured BBL domains was assessed using operando electrochemical ^2^H NMR spectroscopy, which distinguishes water inside the polymer from bulk electrolyte^32^. This distinction is enabled by the quadrupolar nature of the spin-1 ^2^H nucleus, whereby water confined within ordered BBL domains produces a characteristic doublet with a 1.5 kHz quadrupolar splitting before doping, while isotropic bulk water appears as a singlet at 216 Hz (4.7 ppm; Fig. 2a,g and Supplementary Note 2). Operando ^2^H NMR measurements were performed on BBL films in 0.1 M NH_4_Cl or NaCl during cyclic voltammetry (CV) sweeps, and representative ^2^H NMR slices were extracted from the contour plots (Fig. 2a,b for NH_4_Cl and Fig. 2g,h for NaCl). The current response is consistent with the expected electrochemical behaviour of BBL in both electrolytes (Fig. 2c,d for NH_4_Cl and Fig. 2i,j for NaCl). The ^2^H NMR spectra show voltage-dependent and charge-dependent changes in both quadrupolar splitting and intensity of the ‘anisotropic’ water (Fig. 2e,f for NH_4_Cl and Fig. 2k,l for NaCl). The doublet intensity (Fig. 2e,k) directly reflects changes in the water population within the anisotropic BBL domains, while the splitting (Fig. 2f,l) provides an indirect probe of water content and polymer-domain orientation. Further experimental and theoretical details are provided in Supplementary Note 2.

Water movement inside BBL films is directly tracked from the evolution of the anisotropic-water signal during the CV sweep. In NH_4_Cl, this signal increases at low doping levels and then decreases at higher bias (Fig. 2c,e), with the downward sweep (that is, decreasing the applied bias and recording a positive current (Fig. 2c,d)) mirroring this trend. The intensity profile in Fig. 2e closely matches the EQCM-D mass changes (Supplementary Fig. 10), confirming water expulsion from the BBL film on electrochemical doping beyond a certain threshold. The decrease in ^2^H NMR intensity (~30%) is consistent with the reduction in mass (~25%) and thickness (~30%), compared to their values of maximum increase, indicating that water loss, probably due to reduced cation hydration, accounts for the observed reductions in both mass and thickness. The plateauing observed in EQCM-D and EC-AFM at high bias, which contrasts with the continuously decreasing ^2^H NMR signal, suggests a competition between cation uptake and water expulsion at these high doping levels.

In NaCl, in contrast, the anisotropic-water signal does not decrease at maximum doping. Instead, both intensity and peak splitting plateau beyond ~−0.7 V (Fig. 2k,l), even though additional charges are injected (Fig. 2j). This plateau explains the inflection point observed in EC-AFM and EQCM-D at ~−0.7 V (Fig. 1c,d), suggesting that Na^+^ becomes less hydrated at high doping but does not drive net water loss from the film. Thus, while both electrolytes reach a regime of reduced cation hydration, only NH_4_Cl results in measurable water expulsion, pointing to a distinct interaction mechanism between BBL and NH_4_^+^ rather than a simple concentration-dependent ion-dehydration effect (Supplementary Note 2). The reduced hydration detected by ^2^H NMR at high doping levels arises primarily from the loss of cation-solvating water, with a secondary contribution from chloride expulsion, supported by operando ^35^Cl NMR (Supplementary Note 3). Further discussion of water movement inferred from ^2^H NMR, EC-AFM and EQCM-D is provided in Supplementary Notes 2 and 4.

## Cation–BBL interactions

**Fig. 3:**
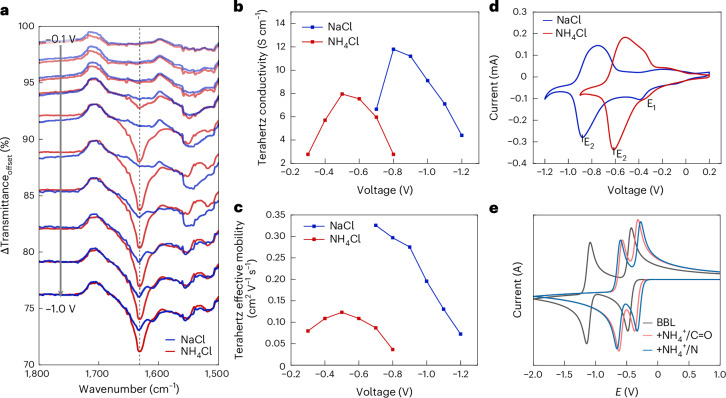
Cation–carbonyl interactions and charge localization from IR and terahertz spectroscopy and CV. **a**, Operando differential IR transmittance spectra focusing on the carbonyl vibration region of BBL doped in 0.1 M NaCl (blue) and NH_4_Cl (red). Applied biases range from −0.1 V to −1.0 V versus Ag/AgCl with −0.1 V steps. An increasing absorption yields a lower Δtransmittance, while the loss of an absorption peak translates to a positive contribution in this graph. **b**,**c**, Terahertz conductivity (**b**) and short-range mobility (**c**) as a function of applied bias for BBL submerged in 0.1 M NaCl (blue) or NH_4_Cl (red). The higher short-range conductivity compared to standard macroscopic (OECT) conductivity can be attributed to the influence of long-range defects, traps, grain boundaries and/or disorder, which increasingly affect charge transport over longer distances, a characteristic commonly observed in organic semiconductors^42^. **d**,**e**, The measured CV in 0.1 M NaCl and NH_4_Cl (**d**) and the simulated CV with or without hydrogen bonding (**e**). Calculations are performed on the syn-form of BBL, while results for the anti-form are provided in Supplementary Note 6. Source data

The striking differences between NH_4_Cl and NaCl suggest strong cation–BBL interactions during electrochemical doping. To investigate this, we performed operando infrared (IR) spectroscopy, which is particularly sensitive to vibrations involving BBL’s heteroatoms (Methods). Differential IR transmittance spectra were recorded as BBL was doped from −0.1 V to −1.0 V versus Ag/AgCl in 0.1 M NaCl or NH_4_Cl (Supplementary Figs. 11–13), revealing spectral changes consistent with previous studies^33,34^. Figure 3a compares the carbonyl (C=O) vibration region for both electrolytes (Δtransmittance; pristine spectrum in Supplementary Fig. 14). Up to ~−0.3 V, the IR spectra are identical (Supplementary Fig. 11), with the original C=O vibration (~1,710 cm^−1^) evolving into a broad absorption band (1,685–1,600 cm^−1^), in line with earlier reports showing similar conductivities in this regime^8^. Beyond −0.4 V, however, a strong absorption at ~1,632 cm^−1^ emerges for NH_4_Cl, whereas a similar feature appears for NaCl only at ~−0.7 V and intensifies towards −1.0 V. This band has been attributed to either partial protonation of the imine (C–N(H)–C deformation)^35^ or to a red-shifted C=O stretch vibration arising from increased partial negative charge on the carbonyl^36^. At maximum doping, both electrolytes yield similar IR spectra, but the ~1,632 cm^−1^ band is more intense for NH_4_^+^ (Supplementary Fig. 11), indicating a larger transition dipole and stronger interaction. These observations are supported by density functional theory (DFT) calculations suggesting that both hydrogen bonding and partial protonation contribute to this behaviour (Supplementary Note 5).

Notably, the onset of this strong vibration in both electrolytes coincides with the deswelling observed in EQCM-D and EC-AFM, as well as with the conductivity maximum reported for OECTs^8^ and terahertz spectroscopy (vide infra). In addition, the aromatic core breathing mode at ~1,540 cm^−1^ and free-electron absorption at higher wavenumbers follow the conductivity–voltage trend^13,37,38^ (Supplementary Figs. 15 and 16). These results indicate that charge localization underlies the characteristic conductivity maximum (antiambipolarity) in BBL^8,26^.

To further investigate how cations influence short-range electronic transport, we performed operando terahertz spectroscopy (Supplementary Fig. 17 and Methods). From the terahertz measurements, we extracted conductivity, short-range mobility and charge density as functions of applied bias^39,40^ (Fig. 3b,c and Supplementary Fig. 18). The terahertz conductivity for both electrolytes (Fig. 3b) mirrors the macroscopic OECT conductivity^8^, showing a pronounced maximum, indicating that short-range effects play a key role in BBL antiambipolarity. The effective mobility extracted from the Drude–Smith model (Methods) decreases sharply beyond this maximum (Fig. 3c), governed by an increasingly negative localization parameter (Supplementary Fig. 19). This trend is consistent with the IR data and supports the view that charge localization at high doping levels gives rise to the observed antiambipolar behaviour.

## Hydrogen bonding, protonation and water movement

CVs of BBL gated in NH_4_Cl and NaCl exhibit clear differences (Fig. 3d). Both electrolytes display a broad feature between −0.2 V and 0 V versus Ag/AgCl (E_1_), but in NH_4_Cl, the main redox peak (E_2_) is shifted by ~0.3 V towards lower potentials. This suggests enhanced stabilization of reduced BBL species by NH_4_^+^, probably via hydrogen bonding and/or protonation involving carbonyl and imine groups in the BBL backbone^41^. To investigate this, we performed extensive DFT calculations using perinone (BBL’s monomer) interacting with both hydronium and ammonium ions across models of varying complexity (Supplementary Note 6). Gibbs free energies from DFT were used in a thermodynamic square scheme including electron transfer and protonation steps, and simulated CVs were generated via Butler–Volmer kinetics.

The DFT results span from full protonation to weak hydrogen bonding, depending on doping level, and are sensitive to subtle differences in ion arrangement and solvation. The imine shows stronger proton affinity than the carbonyl, and some protonation is possible for both cations. However, the simulated CVs suggest only limited protonation at the experimental pH (5–7). These simulations align with experimental CVs recorded in NaCl and various amounts of HCl but do not reproduce the E_2_ shift observed with NH_4_Cl (Supplementary Note 6).

In contrast, when explicit hydrogen bonding between water-solvated NH_4_^+^ and either the imidazole nitrogen or carbonyl oxygen is included, the redox potentials—particularly for the doubly reduced state—are notably stabilized (Fig. 3e). This results in a reduced E_1_–E_2_ separation by ~0.3 V, consistent with the experimental CV response in NH_4_Cl (Fig. 3d). We therefore conclude that the pronounced changes observed on switching from NaCl to NH_4_Cl arise primarily from stronger ammonium–BBL interactions dominated by hydrogen bonding and potentially accompanied by partial protonation of the imidazole groups for both cations.

**Fig. 4:**
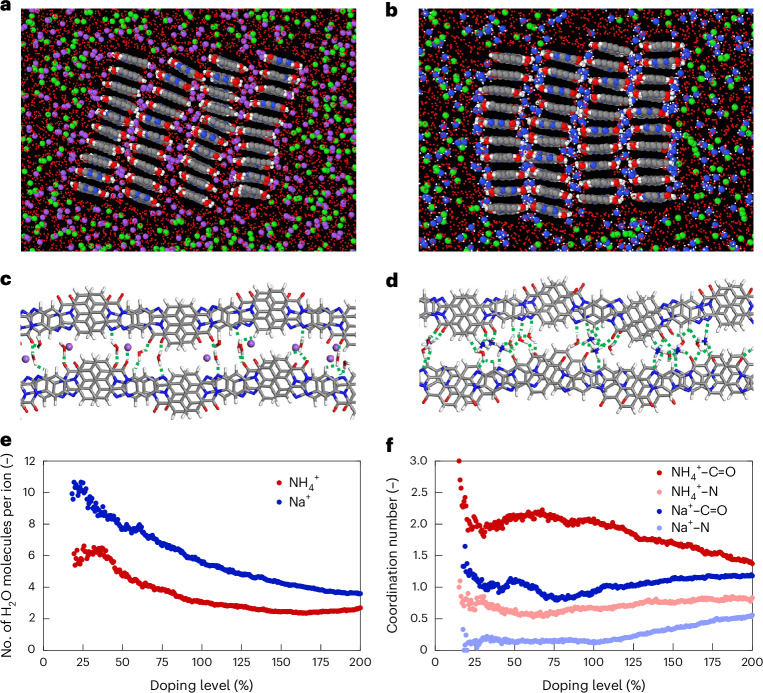
Cation–BBL interactions from MD simulations. **a**,**b**, Representation of the simulated BBL:ion systems at 100% doping level (Na^+^ (**a**), NH_4_^+^ (**b**)). The doping level percentage denotes the number of electrons per BBL repeat unit, with 200% representing the fully doped state with 2 electrons per repeat unit^8^. The atoms are colour-coded as follows: Na (purple), N (blue), Cl (green), O (red), C (grey) and H (white). For clarity, water molecules are depicted as small red spheres. See technical details in the Methods. **c**,**d**, Zoomed visualization of the interlayer regions for BBL:ion systems at 100% doping level (Na^+^ (**c**), NH_4_^+^ (**d**)). The green dashed lines correspond to H-bonds. **e**, Evolution of the number of water molecules per inserted ion on electrochemical doping. **f**, Coordination numbers of the ions with the oxygen of the carbonyl groups or the nitrogens of the BBL chains. The cut-off distances are set to 3.0 Å for Na^+^ and 3.5 Å for NH_4_^+^. Source data

To further probe short-range interactions, molecular dynamics (MD) simulations of a BBL crystallite in NH_4_Cl or NaCl were conducted. These simulations reveal gradual insertion of both ions and water between BBL stacks on doping (Fig. 4a,b). In the interlayer region, NH_4_^+^ (Na^+^) ions are coordinated by ~6 (~10) water molecules at ~25% doping, decreasing to ~3 (~6) at 100% doping (Fig. 4e), consistent with ^2^H NMR observation of decreased cation hydration. NH_4_^+^ forms hydrogen bonds primarily with carbonyl groups and, to a lesser extent, with nitrogen atoms (Fig. 4c,d). Counting ion-BBL contacts from the MD trajectories shows that, at 100% doping, the C=O^−^–H–NH_3_^+^ coordination number is ~2, twice that for C=O^−^–Na^+^, indicating a more extensive hydrogen-bonded network (Fig. 4f). In addition, average interaction energies from MD are 5–10 kcal mol^−1^ per ion higher for NH_4_^+^ than for Na^+^ (Supplementary Fig. 20). We note that the MD simulations use a 4 M ion concentration to achieve feasible simulation timescales, which affects relative permittivity and ion hydration shell compared to the 0.1 M experiments.

Although these simulations provide qualitative insight, they do not fully explain the mechanism behind deswelling. Both cations show gradually shrinking solvation shells, leading to slower water uptake rather than net water loss. On the basis of the CV and IR simulations, we proposed that partial protonation of the imidazole group might promote deswelling. To test this, we performed additional MD simulations in which covalent N–H-bonds were manually enforced (the classical force field does not support bond formation). After relaxation, we observed up to a 35% reduction in water content and the emergence of a more ordered phase featuring water monolayers intercalated between BBL layers (Supplementary Note 7). While further investigation is warranted, these results indicate that partial protonation facilitates the formation of a hydrogen-bonding network that drives water expulsion at high doping levels. Protonated, fully reduced BBL chains also exhibit a notable bandgap opening near the Fermi level (Supplementary Note 8), which may contribute to the reduced conductivity at full doping.

## Methylated ammonium–BBL interactions

**Fig. 5:**
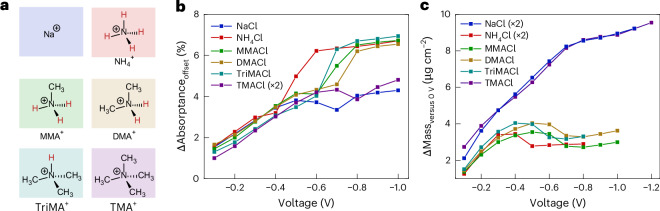
Cation–BBL interactions from doping with methylated ammonium chloride salts. **a**, Chemical structures of the cations used. **b**, The differential IR absorptions at 1,633 cm^−1^. **c**, ΔMass (from the Sauerbrey equation) of a BBL film submerged in NaCl (blue), NH_4_Cl (red), MMACl (green), DMACl (yellow), TriMACl (light blue) and TMACl (purple) electrolytes at 0.1 M as function of applied bias relative to the preswollen 0 V mass. The (×2) denotes that TMACl IR absorption and NaCl/NH_4_Cl Δmass datapoints are multiplied by two for ease of comparison. A comparison of the absolute mass changes for selected cations is provided in Supplementary Figs. 27 and 28. The various electrolytes are colour-coded as indicated in the schematic on the left. Source data

To further explore cation–BBL interactions driving the mass and thickness reduction observed in NH_4_Cl, we investigated the electrochemical doping of BBL using several methyl-substituted ammonium cations (Fig. 5a). Differential IR transmittance and Δmass were recorded as functions of applied bias versus Ag/AgCl for monomethylammonium chloride (MMACl), dimethylammonium chloride (DMACl), trimethylammonium chloride (TriMACl) and tetramethylammonium chloride (TMACl) using IR spectroscopy and EQCM-D (Supplementary Figs. 21–26). Figure 5b,c summarizes the differential IR absorptance at 1,633 cm^−1^ and Δmass (relative to 0 V) for each cation.

In Fig. 5b, all electrolytes show an initial absorptance increase at low bias due to the tail of the free-charge absorption. At higher biases, this contribution diminishes first and then the strong vibration emerges, leading to a pronounced Δabsorptance increase following a small dip. For NH_4_Cl, this vibration dominates at ~−0.5 V, whereas for NaCl it appears only near ~−0.8 V and with a weaker increase, as discussed above. Methyl-substituted ammonium salts show distinct behaviour: protic cations (MMA^+^, DMA^+^ and TriMA^+^) exhibit the same strong absorption as NH_4_^+^, whereas TMA^+^ resembles Na^+^ (Supplementary Fig. 22). EQCM-D (Fig. 5c) similarly reveals that protic cations induce a characteristic Δmass decrease at high doping levels, while TMA^+^ again behaves like Na^+^. Notably, the bias at which mass decreases and the new vibration appears follows the cation p*K*_a_ order (NH_4_^+^ < TriMA^+^ < MMA^+^ < DMA^+^; Supplementary Table 1), reflecting the electropositivity of the hydrogen atom and its potential to form H-bonds. Although the redox peaks in the CV shift markedly with the choice of cation, these shifts do not correlate with bulk electrolyte pH, which changes only slightly (Supplementary Fig. 29). This indicates that hydrogen bonding rather than protonation underlies the stronger cation–polymer interaction, consistent with CV simulations (Supplementary Note 6). The onset bias also matches the bias at which conductivity maxima occur for these electrolytes^8^, indicating that the cation determines the onset of charge localization.

## Outlook

The results above indicate a clear correlation between strong cation–polymer interactions and the unique mass and thickness responses of BBL, which coincide with its electrical antiambipolarity, as the onset biases for these phenomena match. However, the fact that TriMA^+^ also induces a mass decrease at higher doping levels excludes the possibility that protic cations simply act as bridging bonds, since TriMA^+^ has only one highly polarized hydrogen atom available for hydrogen bonding. Instead, we propose that the formation of a hydrogen-bonding network drives the observed water expulsion for protic cations. Combined IR and CV measurements and simulations support contributions from both hydrogen bonding and partial protonation, with stronger interactions for protic cations. MD simulations further show that hydrogen-bonding network formation can promote deswelling via water expulsion. Altogether, we argue that protic cations facilitate a more extensive hydrogen-bonding network that underlies the water loss observed in EQCM-D, EC-AFM and ^2^H NMR. This network probably arises from a complex interplay of partial protonation and hydrogen bonding with solvated cations, making it challenging to fully resolve computationally. Nonetheless, the CV simulations and the systematic behaviour of methylated ammonium cations suggest that hydrogen bonding is the primary driver of cation–polymer interactions governing charge localization, electrochemical response and mass changes in BBL at high doping. The deswelling observed at high doping levels is expected to enhance device stability during repeated cycling, as confirmed by stability measurements (Supplementary Fig. 30). Beyond stability, deswelling also offers opportunities for optical modulation in organic metasurfaces. Operando ellipsometry combined with optical modelling shows that the ~14% thickness decrease relative to the peak value (Fig. 1b) yields an optical-path length change comparable to that of state-of-the-art OMIEC metasurfaces^25^, owing to the high refractive index of BBL (Supplementary Note 9).

In summary, we uncovered how cation–polymer interactions drive a unique deswelling mechanism during the electrochemical doping of the n-type polymer BBL. EQCM-D and EC-AFM show pronounced mass and thickness decreases with NH_4_Cl, while operando ^2^H NMR confirms water expulsion. Operando IR and terahertz spectroscopies reveal that these interactions induce partial charge localization, reducing conductivity and, for protic cations, triggering mass loss, supported by DFT and MD simulations. Our study provides mechanistic insights into ion–polymer interactions in OMIECs, enabling optimized electrolyte–material combinations and improved device control. This deswelling mechanism challenges existing paradigms and provides structure–property relations relevant for applications spanning bioelectronics to photonics.

## Methods

### Materials

BBL was synthesized according to a previously reported procedure in ref. ^43^. NaCl (≥99.0%), MMACl (≥98%), TMACl (≥99.0%), methanesulfonic acid (MSA, ≥99.0%) and HCl (37% in water) were purchased from Sigma-Aldrich. DMACl (≥98.0%) and TMACl (≥98.0%) were obtained from Merck, and NH_4_Cl (≥99.5%) was obtained from Sigma.

### Polymer casting

BBL was dissolved in MSA at 2 mg ml^−1^ and spin-coated at 1,000 rpm for 60 s onto Ti/Au-coated quartz crystal (EQCM-D), Cr/Au-coated wafer (EC-AFM, vide infra) or back-etched silicon wafers (IR). For terahertz conductivity, the same solution was drop-cast onto quartz substrates with patterned gold contacts. For operando ^2^H NMR, thicker films, required to achieve reasonable signal-to-noise ratios, were prepared by drop-casting 10 mg ml^−1^ or 20 mg ml^−1^ BBL solutions in MSA onto glass. After deposition, films were soaked in isopropanol (>5 min) and demineralized water (>15 min) and dried under N_2_. Drop-cast ^2^H NMR films detached during soaking and were dried in air by pressing them onto lint-free cloth to obtain free-standing flakes. For GIWAXS, BBL was spin-coated from a 10 mg ml^−1^ MSA solution (1,000 rpm, 60 s) onto silicon substrates and rinsed in deionized water. For operando GIWAXS, films were lifted off in water and transferred to gold-coated porous silicon substrates.

### CV

CV was performed using a three-electrode setup comprising the BBL-coated working electrode, a Pt mesh counter electrode and an Ag/AgCl pellet reference electrode. Measurements were carried out using a BioLogic SP200 potentiostat at a scan rate of 50 mV s^−1^ unless otherwise stated.

### EQCM-D

Ti/Au-coated quartz crystals (QSX 338, Biolin Scientific) were coated with BBL (see above) and mounted in a QSense module (Biolin Scientific) filled with electrolyte of interest. QCM data (frequency and dissipation shifts) were recorded using a QSense Analyzer (Biolin Scientific). Electrochemistry was performed using the Ti/Au-coated quartz crystal as the working electrode, a Pt film counter electrode, and an Ag/AgCl reference electrode (Dri-Ref-2SH, World Precision Instruments). A potentiostat (µAutolab, Metrohm) was used to apply the bias. Measurements typically consisted of 8 CV cycles (0.05 V s^−1^) from the lowest stable potential to +0.2 V versus Ag/AgCl, followed by a stepwise increase in applied bias from this lowest stable potential to +0.2 V in steps of 0.1 V for 60 s, with intermittent voltage holds at 0 V versus Ag/AgCl for 60 s. Qsense Dfind software (Biolin Scientific) was used to calculate the Δmass from the Sauerbrey equation (third overtone) or through viscoelastic modelling using the Dfind Broadfit algorithm.

### EC-AFM

Glass substrates were cleaned by successive sonication in acetone, deionized water and isopropyl alcohol, and dried with N_2_. Cr (5 nm) and Au (50 nm) layers were thermally deposited, after which BBL was spin-coated from MSA solution, rinsed with deionized water and dried under N_2_. The films were patterned by photolithography and plasma etching. EC-AFM measurements were performed on a Dimension Icon XR (Bruker) using an electrochemical cell with a Pt wire counter electrode and an Ag/AgCl pellet reference electrode. Imaging was conducted in off-resonance mode using silicon nitride probes having a nominal tip radius of 20 nm and a spring constant of 0.7 N m^−1^. Films were initialized by eight CV cycles at 0.05 V s^−1^, after which measurements were performed by stepping the potential from negative to positive, allowing at least 60 s of equilibration before imaging. Image distortions were corrected using a first-order plane fit, and the film thickness was obtained as the height difference between the two dominant features (gold substrate and polymer film) measured from the peak-to-peak distance of Gaussians fitting the raw data histograms.

### GIWAXS

GIWAXS measurements were performed at the 11-BM CMS beamline at NSLS-II with a Pilatus 300K detector. A beam energy of 13.5 keV was used for all measurements. The distance between the sample and the detector was calibrated using a BeH standard. For ex situ measurements, an angle of incidence of 0.14° was used with a 10 s exposure time. For operando measurements, an electrochemical cell (Supplementary Fig. 31) was placed in a sealed chamber with Kapton windows, where humid N_2_ was continuously flowing to reduce oxygen side reactions. The whole chamber was kept in vacuum to minimize air scatter. The X-ray scattering data were reduced in Python using the open-source pyFAI and pygix modules^44,45^, following the general procedure reported in refs. ^25,46^. The out-of-plane (*q*_*z*_) and in-plane (*q*_*xy*_) linecuts were taken from cake slices from *χ* = −20° to 20° and 65° to 87° with respect to the *q*_*z*_ direction, respectively. Small-angle scattering between *χ* = −3° and 3° was excluded from the out-of-plane linecuts. Scattering peaks were fitted to Gaussian–Lorentzian lineshapes using the lmfit package in Python v.3.9. Electrochemical measurements were taken on a Gamry potentiostat in a three-electrode setup with a leakless Ag/AgCl reference pellet (eDAQ) and a platinum wire counter electrode. The electrolytes (0.1 M NaCl and 0.1 M NH_4_Cl) were degassed before cell assembly.

### Operando IR absorption spectroscopy

A back-etched silicon wafer (universal ATR element, IRUBIS) was sputter-coated with indium tin oxide while at 300 °C, yielding ~20 nm indium tin oxide with a sheet resistance of ~220 Ω per square. This wafer was subsequently covered with BBL as described above. The sample was loaded into a spectroelectrochemical cell (Jackfish J1, PIKE Technologies) and mounted atop the Veemax III accessory (PIKE Technologies) for use with the Fourier transform IR spectrometer (Spectrum 3, PerkinElmer). The spectroelectrochemical cell was filled with the chosen electrolyte. A Pt wire counter electrode and an Ag/AgCl reference electrode (in saturated KCl with glass frit, PN 162-4723 PIKE Technologies) were submerged in the electrolyte to complete the three-electrode setup. Spectra were recorded using a liquid nitrogen-cooled mercury cadmium telluride detector and with the Veemax III at 55°. Measurements spanned 4,000–450 cm^−1^, with a 1 cm^−1^ datapoint spacing, and included 50 repeats. Bias was applied for 80 s (SP200 potentiostat, BioLogic) during spectral acquisition, followed by a 30-s dedoping step at 0 V versus Ag/AgCl. Pristine BBL films submerged in electrolyte were used as the background IR spectrum, and the differential transmittance spectra reported were referenced to this background.

### Operando ellipsometry

Spectroscopic ellipsometry was measured with an RC2 Mueller Matrix ellipsometer (J.A. Woollam) using an electrochemical ellipsometry cell (redox.me). At a 70° incident angle, the ellipsometer response of a layer of BBL on a Cr/Au-coated Si substrate was recorded under varying applied bias. Baseline measurements were performed on the empty cell (with substrate) and on the cell filled with electrolyte to determine the electrolyte refractive index and the specifications of the substrate used (including Au/Cr). Fitting was performed using CompleteEase (J.A. Woollam).

### Terahertz spectroscopy

Terahertz time-domain spectroscopy was performed using an ultrafast Ti:sapphire amplified laser system (Astrella, Coherent; 35-fs pulse duration, 800-nm centre wavelength, 1-kHz repetition rate, 6 W). Terahertz pulses were generated by optical rectification of part of the fundamental beam in a ZnTe crystal and directed onto the sample using two off-axis parabolic gold mirrors. The transmitted terahertz pulses were refocused by two additional off-axis parabolic mirrors onto a second ZnTe crystal for detection, where they overlapped spatially and temporally with a gate pulse derived from the fundamental beam, and electro-optic sampling was used. The terahertz pulses induced a birefringent refractive index in the detection ZnTe crystal, altering the polarization of the gate pulses from linear to slightly elliptical. The elliptically polarized gate beam was passed through a quarter-wave plate to render it nearly circularly polarized. The two orthogonal polarizations were split by a Wollaston prism and focused onto a balanced photodiode, which measured the intensity difference between them, proportional to the electric field. By changing the time delay between the terahertz and gate pulse using a micrometre translation stage, the terahertz electric field was measured at different time delays. The resulting terahertz pulses had a duration of ~1 ps and a spectral bandwidth of 0.1–2.5 THz.

For terahertz spectroscopy on electrochemically doped samples, polymer films were deposited between two parallel Au electrodes (5-mm spacing) and mounted in an electrochemical cell. A thin layer of electrolyte (0.1 M NaCl or NH_4_Cl in water) was applied, and an Ag/AgCl pellet electrode served as the reference. The Au electrodes were short-circuited during the terahertz measurements. Frequency-dependent complex conductivity spectra were obtained by measuring the transmitted terahertz electric field through the electrolyte–doped polymer–substrate and a reference transmission through the electrolyte–substrate or electrolyte–neat polymer–substrate. Fourier transforms of both time-domain signals yielded the experimental complex transmission coefficient, which was fitted to a theoretical transmission coefficient accounting for electric-field-induced changes, pulse propagation through each layer, transmission at each interface and multiple reflections within the polymer film to obtain the complex conductivity spectra^40^. The complex terahertz conductivity spectra are analysed using the Drude–Smith model to extract charge-carrier densities and charge mobility contributing to short-range, nanometre-scale transport^41^. In this phenomenological model, the complex conductivity is given by:

$$\mathop{\sigma }\limits^{ \sim }(\omega )=\frac{{\varepsilon }_{0}{\,\omega }_{{\rm{P}}}^{2}\tau }{(1-i\omega \tau )}\left[1+\frac{{c}_{1}}{1-i\omega \tau }\right]$$

where *ω*_P_ is the plasma frequency, *ε*_0_ the vacuum permittivity, *τ* the scattering time and *c*_1_ the localization parameter. The effective short-range mobility was calculated as ${\mu }_{{\rm{eff}}}=\frac{e\tau }{{m}^{\ast }}\,(1+{c}_{1})$, where m* is the effective mass. The short-range mobility reflects charge motion over nanometre distances during the ~1-ps duration of the terahertz pulse.

### Operando ^2^H NMR

The operando NMR cell consisted of a gold mesh (1.3 × 0.5 cm^2^, 0.004 mm thick; Sigma GF14297430) and two free-standing BBL and PEDOT:PSS films (0.6 × 0.5 cm^2^) separated by a borosilicate glass fibre (Whatman, GF/A). PEDOT:PSS (Clevios PH1000, Heraeus Holding) was mixed with 1 wt% 3-glycidoxypropyltrimethoxysilane (Sigma-Aldrich), filtered through a 0.45-µm polyvinylidene fluoride filter and heated at 70 °C for 12 h. The resulting self-standing films were rehydrated in deionized water, rinsed with deionized water (1 h soaking) three times and stored in D_2_O. 0.1 M NaCl or NH_4_Cl in D_2_O was used as the electrolyte. An Ag/AgCl-coated silver wire (0.127 mm diameter; Sigma) served as a pseudo-reference electrode. The cell was configured to detect only one electrode at a time, with the BBL electrode positioned within the NMR coil. Further details of the cell design can be found in refs. ^32,47^. CV was performed using a Biologic SP-100 potentiostat to check the quality of the operando cell before and after the experiments. Operando NMR electrochemical measurements were performed using the same Biologic VSP potentiostat.

Operando ^2^H NMR experiments were performed on a Bruker Avance spectrometer operating at 7.05 T (^2^H Larmor frequencies of 40 MHz) using in-house-designed static and double-resonance probes and a six-turn silver-coated copper solenoidal coil (11 mm diameter). The electrochemical cell was aligned with the electrodes parallel to the magnetic field. Quantitative single-pulse experiments were conducted using a 10-μs (100 W) 90° pulse, a recycle delay of 0.2 s and 32 scans. A spin-lattice relaxation time of 33 ms was measured for anisotropic water with a saturation recovery pulse sequence. Thus, the chosen 0.2 s recycle delay was sufficiently long to ensure quantitative information extraction. Spectra were referenced to D_2_O at 4.75 ppm and processed using Bruker TopSpin v.4.0.5 and MATLAB.

### MD simulation

Ion and water uptake in BBL crystallites were modelled using MD. The system consisted of 4 layers of 10 stacked BBL tetramers surrounded by an explicit electrolyte containing 13,808 water molecules, 1,008 Na^+^ or NH_4_^+^ cations and 1,008 Cl^−^ anions, corresponding to an electrolyte concentration of approximately 4 mol l^−1^. Periodic boundary conditions were applied, ensuring that the BBL tetramers behave as infinite polymer chains. The central BBL crystallite structure was obtained from a conformational search of the syn conformer following established procedures^48,49^. Interatomic interactions were described using the Dreiding force field (Materials Studio software), with atomic charges derived from the DDEC6 partition scheme applied on a periodical DFT charge density^50^. The density was computed on a BBL dimer repeated along the molecular axis using the projected-augmented wave method with the Perdew–Burke–Ernzerhof functional as implemented in the VASP code^51,52^ (see Supplementary Note 8 for details). Water molecules and ions were subsequently added to the BBL crystallite, using the well-established extended simple point charge model to describe interactions involving water molecules^53^.

A 10 ns-long MD simulation (NPT ensemble; *p* = 1 atm, *T* = room temperature) was conducted to equilibrate the system before doping the BBL chains. The doping procedure involved adding one charge every 200 ps during a subsequent NPT simulation. A doping level of 100% (200%) is achieved after 32 (64) ns. Rather than assuming a uniform distribution of charges, atomic charge differences between the neutral and charged BBL were computed from the DDEC6 partition scheme applied on their respective periodical DFT charge densities. A fraction of the charge was assigned to each atom, proportional to these computed changes, thereby properly accounting for differences in electronegativity between atoms. During the doping simulations, atomic coordinates were saved every 200 ps for postprocessing analysis. This procedure was replicated five times for each electrolyte. All simulations, except the conformational search performed in Materials Studio, were carried out using the LAMMPS package^54^, with a 12.0-Å cut off for van der Waals interactions and the particle–particle–particle–mesh method for electrostatic interactions.

To characterize the systems, the number of water molecules and ions within the interlayer regions, the interlayer and/or intermolecular distances, the coordination numbers between ions and the nitrogen and carbonyl groups, and the interaction energies between cations and the BBL layers were analysed. To this end, the centres of mass of each BBL chain were calculated, excluding the first and last chains of each π-stacked layer to avoid edge effects. These 32 points served as reference points to define three surfaces, which were converted into volumes by using the long axis of the BBL polymer chains as the third dimension, as illustrated in Supplementary Fig. 32. Further details on the analysis are listed below:

- The number of water molecules and ions in the interlayer regions was determined by counting the number of species within the three defined volumes for each of the 320 frames saved during the MD simulation.
- The intermolecular distance was calculated as the average distance between BBL chains in a given frame.
- The interlayer distance was measured as the distance between the best-fit plane going through the centres of mass of one layer and the centre of mass of the adjacent layer.
- Coordination numbers between ions and the carbonyl groups or nitrogens of the BBL chains were computed as the average number of oxygen (carbonyl groups) or nitrogen atoms within a radial distance of 3.0 Å (for Na^+^) or 3.5 Å (for NH_4_^+^), relative to the centre of mass of each cation for each frame. The choice of the radial distances was made in such a way as to select only the first coordination shell around the ions.
- Interaction energies were calculated as the sum of the van der Waals and electrostatic interaction energies between the two central BBL layers and the ions located in the interlayer region (excluding water molecules). These energies were then normalized by the number of ions per frame to facilitate comparison between ion types.

## Online content

Any methods, additional references, Nature Portfolio reporting summaries, source data, extended data, supplementary information, acknowledgements, peer review information; details of author contributions and competing interests; and statements of data and code availability are available at 10.1038/s41563-025-02478-2.

## Supplementary information

Supplementary Information Supplementary Figs. 1–71, Notes 1–9 and Tables 1–6.

## Source data

Source Data Fig. 1 Statistical source data, each panel on a separate tab.

Source Data Fig. 2 Statistical source data, each cation on a separate tab.

Source Data Fig. 3 Statistical source data, each panel on a separate tab.

Source Data Fig. 4 Statistical source data, each panel on a separate tab.

Source Data Fig. 5 Statistical source data, each panel on a separate tab.

## Data Availability

The data supporting the findings of this study are available within the paper and its Supplementary Information. Source data are provided with this paper.
