# Supplementary material for: Cation–polymer interactions drive water expulsion and deswelling in n-type ladder organic mixed conductors
Source: Nat Mater. 2026 Feb 11;25(5):832–9. doi: 10.1038/s41563-025-02478-2 (PMC13143810; doi:10.1038/s41563-025-02478-2)
Supplement: Supplementary file 1 — Supplementary Figs. 1–71, Notes 1–9 and Tables 1–6. [file 41563_2025_2478_MOESM1_ESM.pdf]

nature materials

Supplementary information

https://doi.org/10.1038/s41563-025-02478-2

Cation–polymer interactions drive water expulsion and deswelling in n-type ladder organic mixed conductors

In the format provided by the authors and unedited

Contents

EQCM-D on NaCl and NH_4Cl	Figs. 1-5	p. 2-6
Operando AFM micrographs and mass/thickness statistics	Figs. 6-9	p. 7-8
CV sweep in EQCM-D	Figs. 10	p. 9
Extended IR spectra of doping BBL with 0.1 M NH_4Cl and NaCl	Figs. 11-13	p. 10-11
The pristine IR spectrum of BBL	Figs. 14	p. 11
Aromatic core breathing IR absorption of BBL doped in 0.1 M NH_4Cl and NaCl	Figs. 15	p. 12
Comparing mobile electron absorption in IR spectroscopy	Figs. 16	p. 12
THz conductivity spectra	Figs. 17	p. 13
THz conductivity charge density and localization parameter c_1	Figs. 18-19	p. 14
Molecular dynamics simulations	Figs. 20	p. 15
IR of methylated ammonium cations	Figs. 21-22	p. 16
EQCM-D of methylated ammonium cations	Figs. 23-26	p. 17-20
Mass differences from EQCM-D on the same BBL film	Figs. 27-28	p. 21-22
pKa of different ammonium cations	Table 1	p. 22
CV and pH on the methylated ammonia	Figs. 29	p. 23
BBL transfer curve stability	Figs. 30	p. 24
Operando GIWAXS cell	Figs. 31	p. 25
Molecular dynamics analysis regions	Figs. 32	p. 25
**Supplementary Note 1:** (Operando) GIWAXS on BBL	Figs. 33-39<br>Table 2	p. 26-31
**Supplementary Note 2:** ^{2}H NMR	Figs. 40-45	p. 32-39
**Supplementary Note 3:** ^{35}Cl NMR	Figs. 46	p. 40-43
**Supplementary Note 4:** Additional observations on morphological changes upon doping and dedoping from EQCM-D and ^{2}H NMR	Figs. 47-48	p. 44-45
**Supplementary Note 5:** DFT study of IR absorption spectra as a function of charge state and hydrogen bonding	Figs. 49-55<br>Table 3-4	p. 46-52
**Supplementary Note 6:** Computational modelling of cyclic voltammetry data – influence of pH and H-bonding	Figs. 56-62<br>Table 5-6	p.53-59
**Supplementary Note 7**: MD simulations of fully doped protonated samples	Figs. 63-64	p. 60-61
**Supplementary Note 8**: Periodic DFT calculations	Figs. 65-67	p. 62-63
**Supplementary Note 9**: Optical response of BBL upon doping in NaCl and NH_4Cl	Figs. 68-71	p.64-67
**References**	-	p.68-69

EQCM-D on NaCl and NH_4Cl

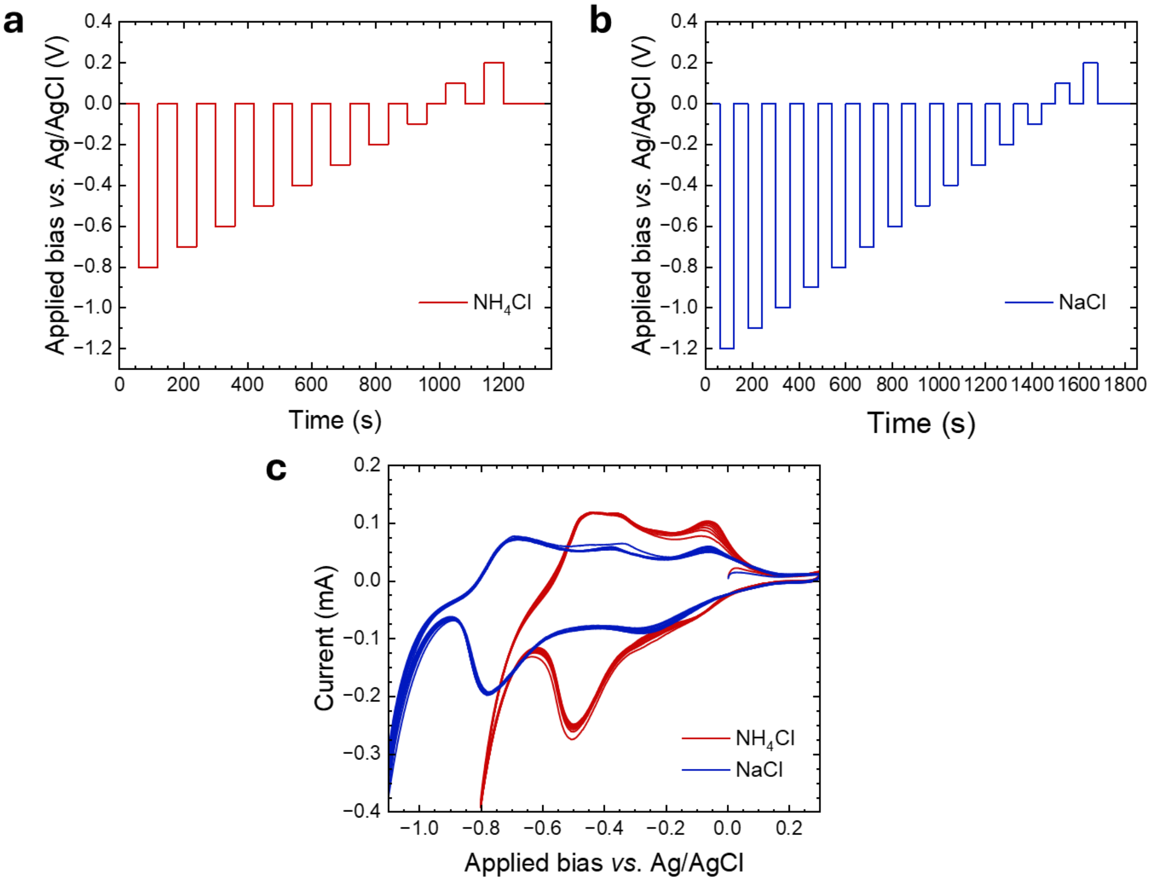

Supplementary Fig. 1 | a-b, The applied bias (vs. Ag/AgCl) during the EQCM-D experiments on BBL submerged in (**a**) 0.1 M NH_4Cl and (**b**) NaCl. **c**, Typical cyclic voltammetry (CV) of BBL gated in 0.1 M NH_4Cl and NaCl at a scan rate of 0.05 V/s.

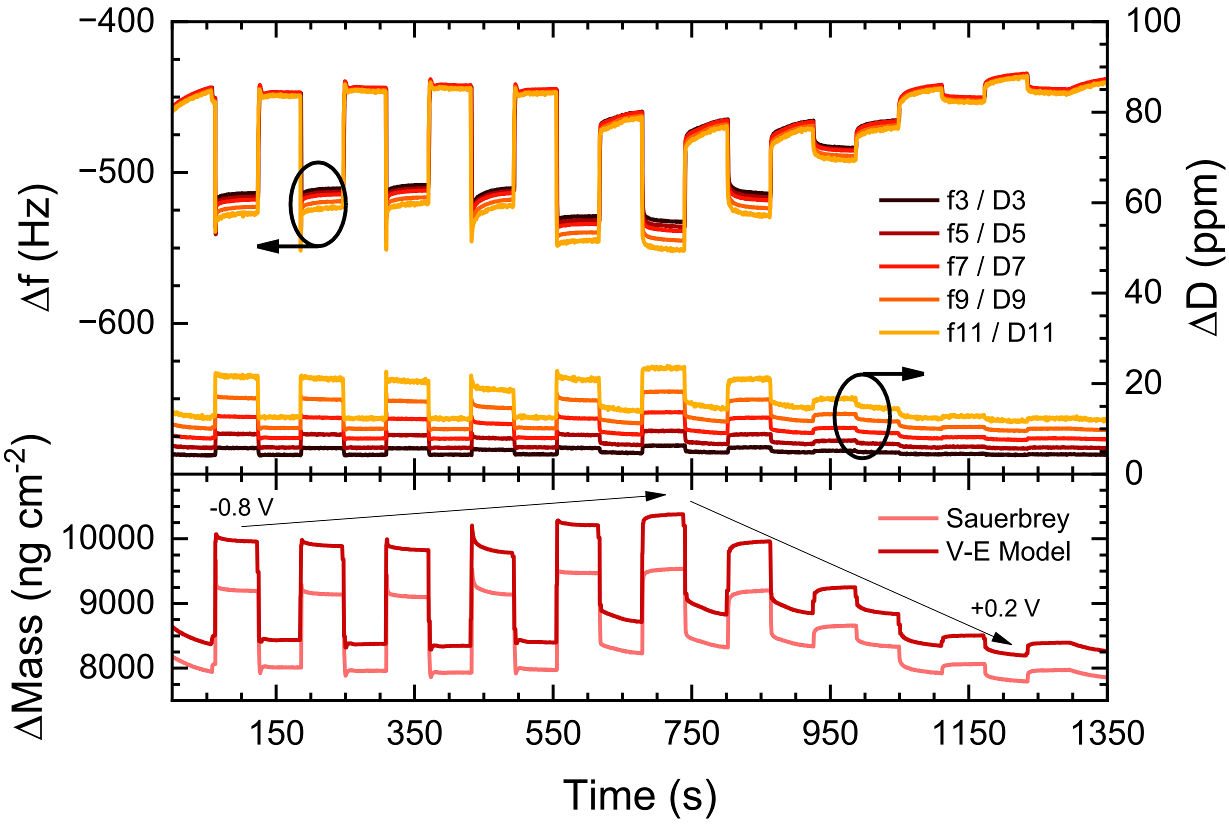

Supplementary Fig. 2 | The measured Δf (left axis) and ΔD (right axis) for the 3rd-11th overtone from EQCM-D measurements on BBL submerged in 0.1 M NH_4Cl. The applied bias is decreased stepwise from -0.8 V to +0.2 V *vs* Ag/AgCl with intermittent 0 V steps. Bottom panel includes the Δmass from the Sauerbrey equation and visco-elastic (V-E) modeling relative to the dry mass of the BBL film.

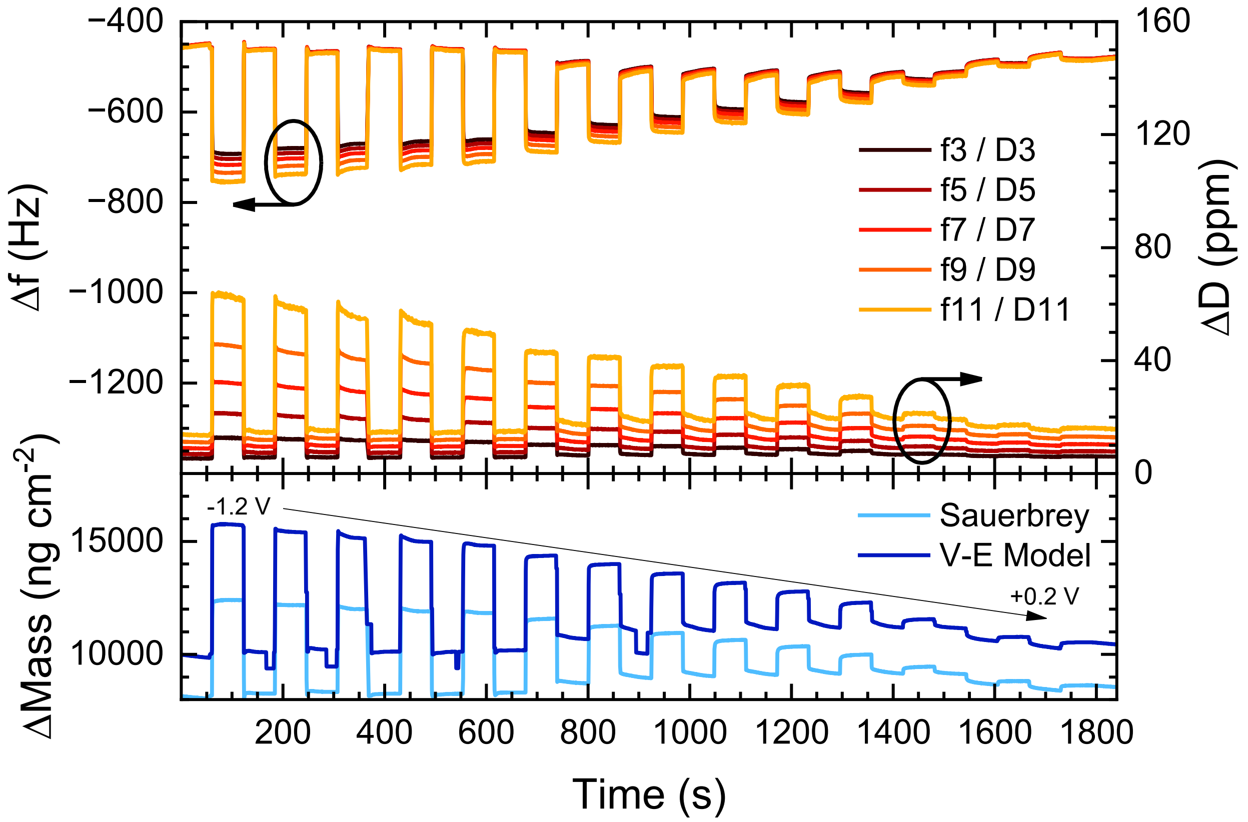

Supplementary Fig. 3 | The measured Δf (left axis) and ΔD (right axis) for the 3rd-11th overtone from EQCM-D measurements on BBL submerged in 0.1 M NaCl. The applied bias is decreased stepwise from -1.2 V to +0.2 V *vs* Ag/AgCl with intermittent 0 V steps. Bottom panel includes the Δmass from the Sauerbrey equation and visco-elastic (V-E) modeling relative to the dry mass of the BBL film.

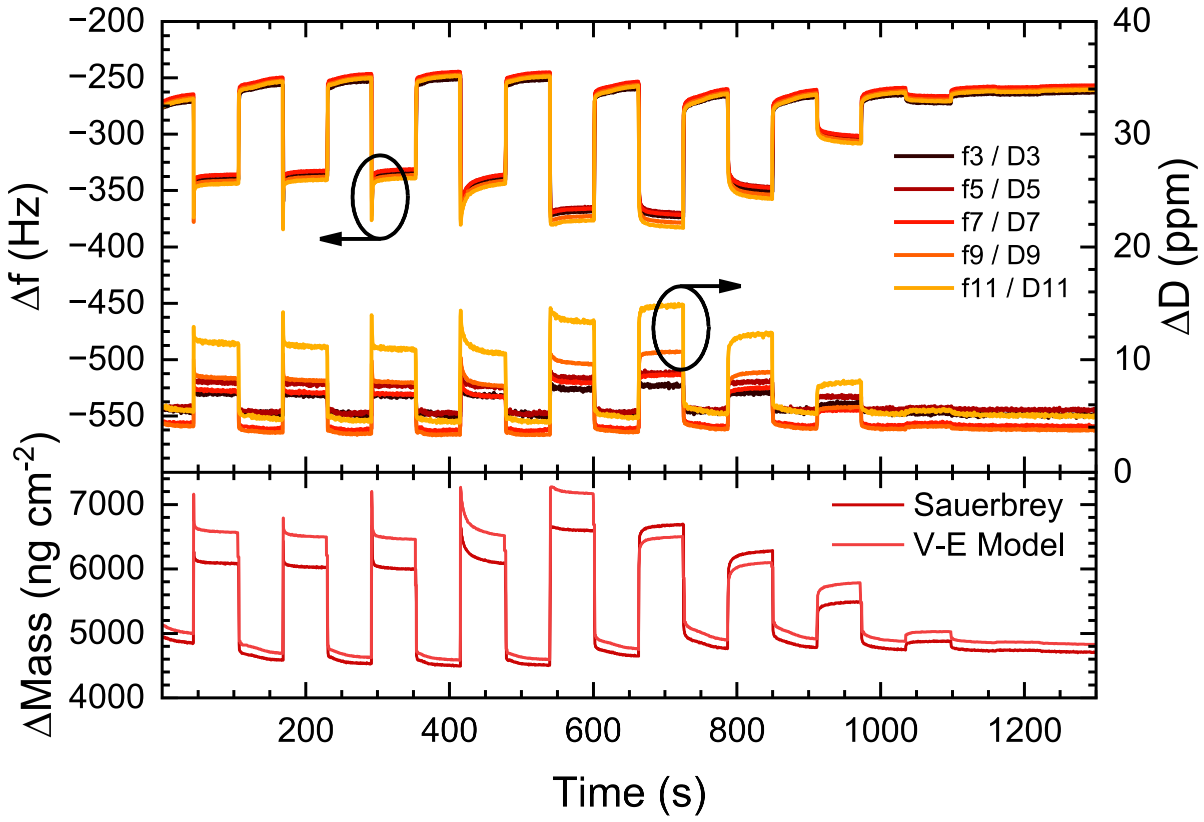

Supplementary Fig. 4 | The measured Δf (left axis) and ΔD (right axis) for the 3rd-11th overtone from EQCM-D measurements on BBL submerged in 0.1 M NH_4Cl. The applied bias is decreased stepwise from -0.8 V to +0.2 V *vs* Ag/AgCl with intermittent +0.2 V steps. Bottom panel includes the Δmass from the Sauerbrey equation and visco-elastic (V-E) modeling relative to the dry mass of the BBL film.

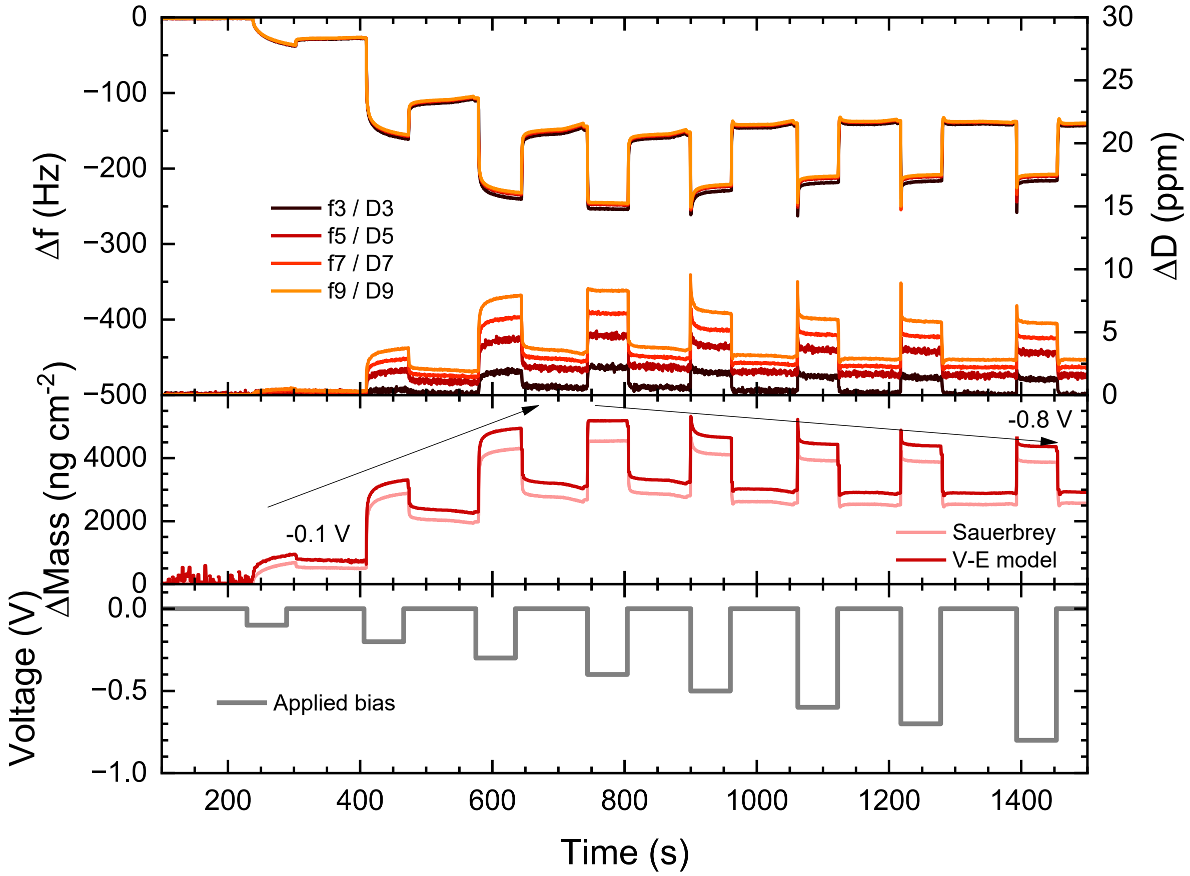

Supplementary Fig. 5 | The measured Δf (left axis) and ΔD (right axis) for the 3rd-9th overtone from EQCM-D measurements on BBL submerged in 0.1 M NH_4Cl. The applied bias is increased stepwise from -0.1 V to -0.8 V *vs* Ag/AgCl with intermittent 0.0 V steps. Middle panel includes the Δmass from the Sauerbrey equation relative to the dry mass of the BBL film. The bottom panel includes the applied bias. Here we find a pronounced remnant initial swelling, which impacts the first few bias steps. For other measurements, great care is taken to precycle the films sufficiently, and we opt to apply decreasing bias steps to alleviate any remnant initial swelling with the first step. Regardless, we crucially find that the observed deswelling for NH_4Cl is independent on the direction of the bias steps, which is also corroborated by the CV data in Supplementary Fig. 10.

Operando AFM micrographs and mass/thickness statistics

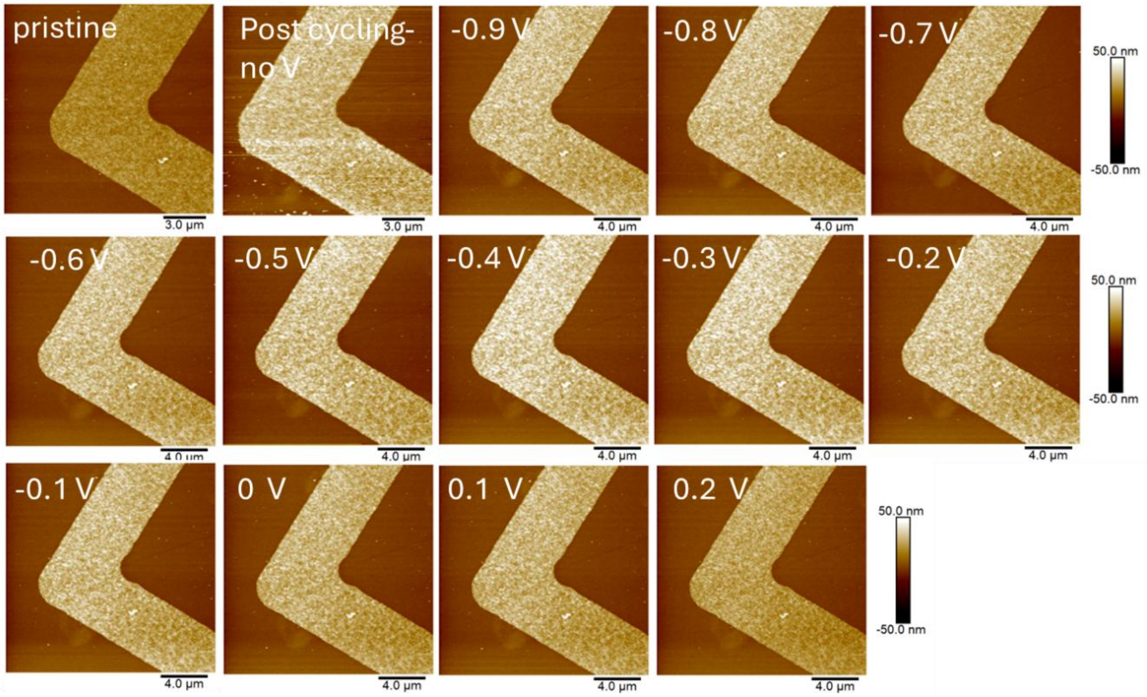

Supplementary Fig. 6 | AFM micrographs of BBL submerged in 0.1 M NH_4Cl upon electrochemical doping at the specified bias (*vs* Ag/AgCl) and pre-/post-electrochemical doping.

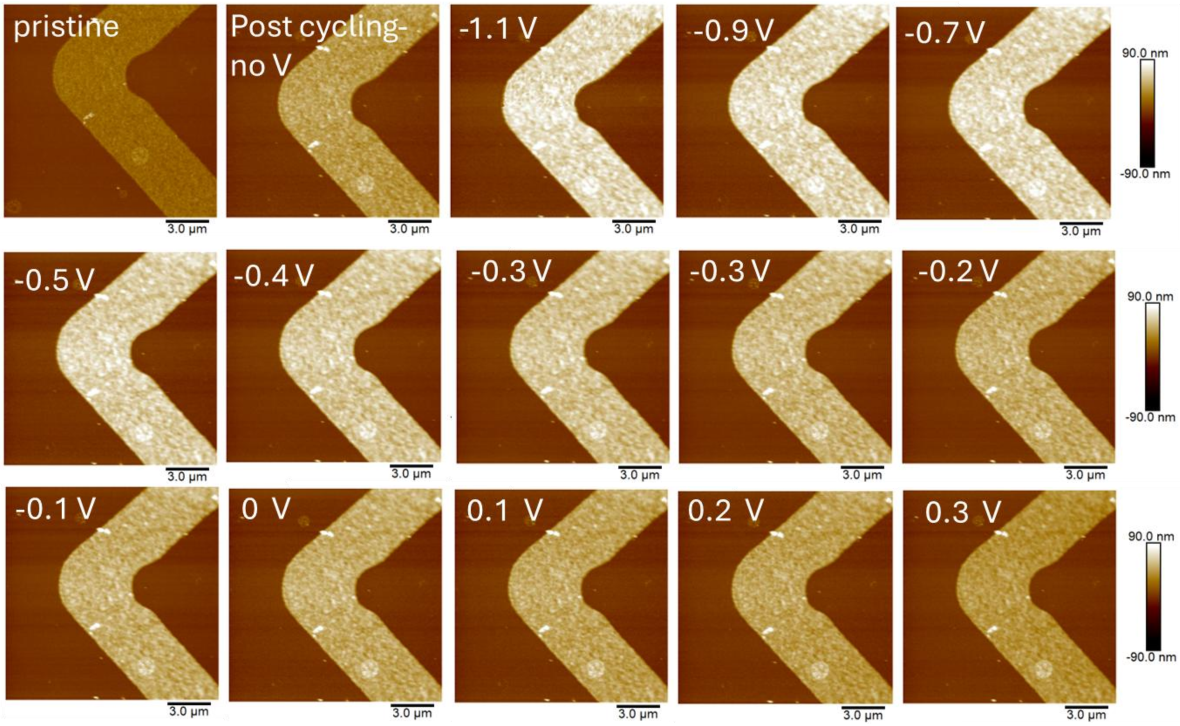

Supplementary Fig. 7 | AFM micrographs of BBL submerged in 0.1 M NaCl upon electrochemical doping at the specified bias (*vs* Ag/AgCl) and pre-/post-electrochemical doping.

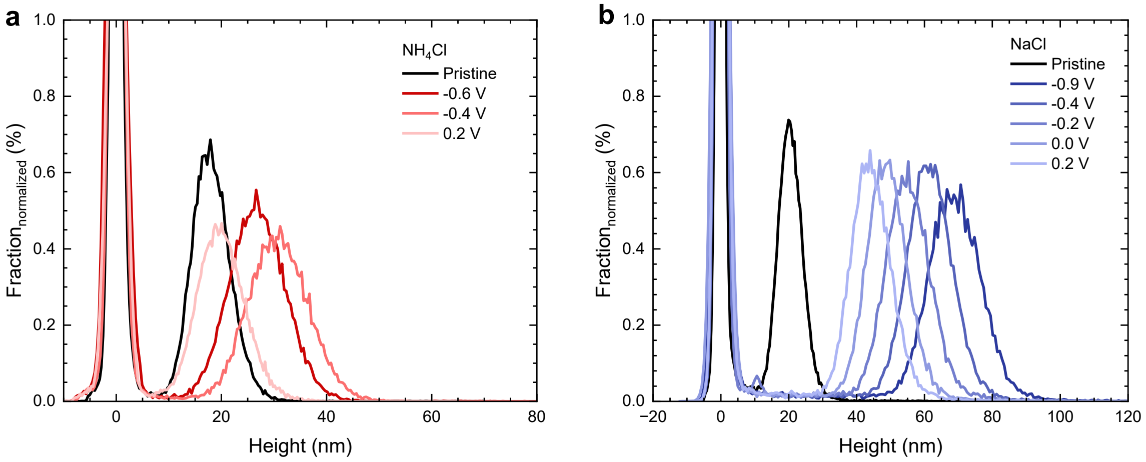

Supplementary Fig. 8 | a,b, The height distribution obtained from the AFM micrographs in Supplementary Fig. 6 (**a**) and Supplementary Fig. 7 (**b**) for doping BBL at selected biases with 0.1 M NH_4Cl and NaCl, respectively. The differences between applied biases are clear regardless the pronounced roughness (*i.e.*, the width in distribution).

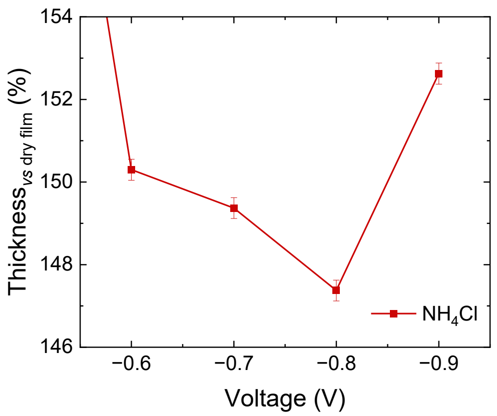

Supplementary Fig. 9 | Zoom-in of Fig. 1d highlighting the error bars. These error bars denote the standard deviation of the mean, including error propagation, obtained from fitting gaussian distributions to the data in Supplementary Fig. 8.

CV sweep in EQCM-D

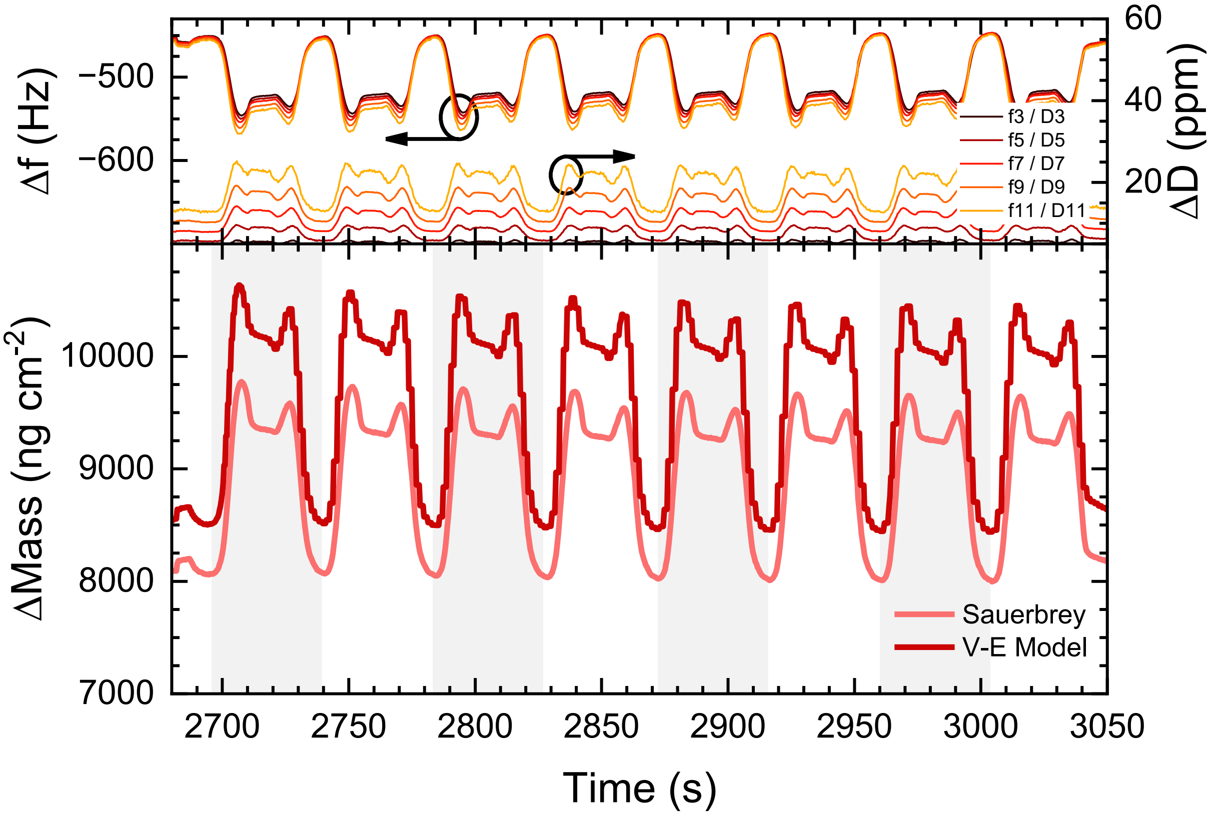

Supplementary Fig. 10 | The measured Δf (left axis) and ΔD (right axis) for the 3rd-11th overtone from EQCM-D measurements on BBL submerged in 0.1 M NH_4Cl. The applied bias is swept from -0.8 V to +0.3 V *vs* Ag/AgCl at 0.05 V s^{-1} for 8 consecutive cycles. Grey areas are guides to the eye denoting a single sweep. Bottom panel includes the Δmass from the Sauerbrey equation and visco-elastic (V-E) modeling relative to the dry mass of the BBL film. There is a clear maximum mass during the sweep, a local minimum at high applied biases, and a temporary increase in mass on the downward sweep.

Extended IR spectra of doping BBL with 0.1 M NH_4Cl and NaCl

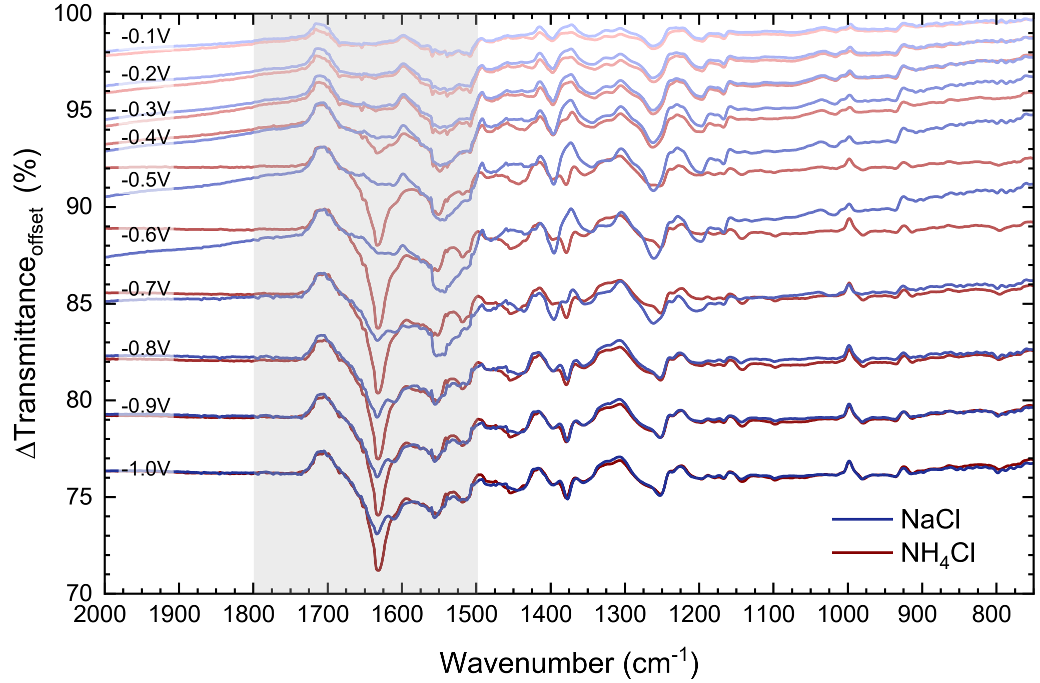

Supplementary Fig. 11 | The fingerprint region of the differential transmittance spectra from operando IR spectroscopy of BBL doped at -0.1 V to -1.0 V while submerged in 0.1 M NaCl and NH_4Cl. The spectra for both electrolytes overlap until -0.3 V and after approximately -0.9 V, with the notable exception of the strong absorption of the new vibration. Spectra as function of bias are offset for clarity and the spectral region of the carbonyl/imine vibration, featured in Fig. 3a of the main text, is highlighted in grey.

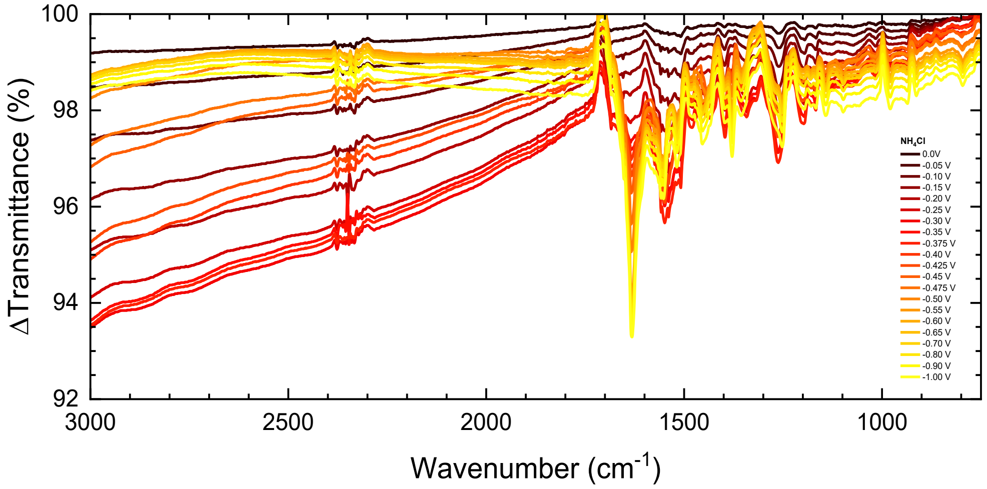

Supplementary Fig. 12 | The differential transmittance spectra from operando IR spectroscopy of BBL doped at -0.1 V to -1.0 V while submerged in 0.1 M NH_4Cl. A significant contribution of absorption by mobile electrons in the high wavenumber region rises upon initial doping, and is later lost at high applied bias. See also Supplementary Fig. 16.

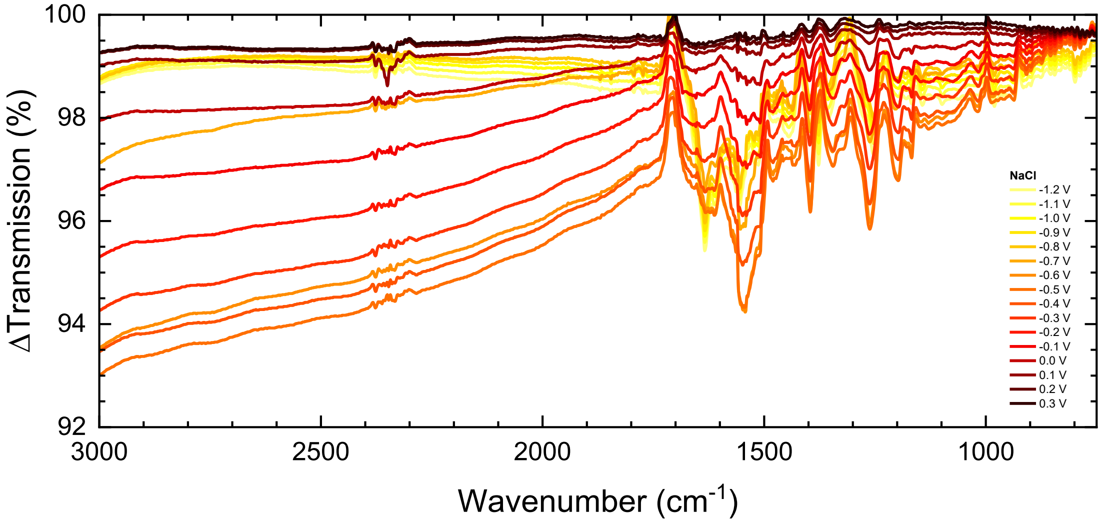

Supplementary Fig. 13 | The differential transmittance spectra from operando IR spectroscopy of BBL doped at -0.1 V to -1.2 V while submerged in 0.1 M NaCl. A significant contribution of absorption by mobile electrons in the high wavenumber region rises upon initial doping, and is later lost at high applied bias. See also Supplementary Fig. 16.

The pristine IR spectrum of BBL

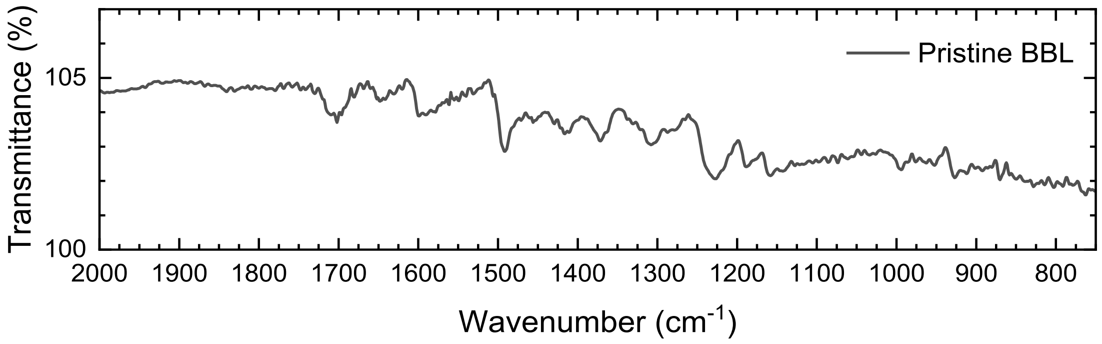

Supplementary Fig. 14 | The transmittance spectrum of pristine dry BBL measured in the operando IR spectroscopy setup exhibiting the typical peaks. Values are above 100% due to the different alignment of the coated crystal compared to the uncoated crystal used as a baseline.

Aromatic core breathing IR absorption of BBL doped in 0.1 M NH_4Cl and NaCl

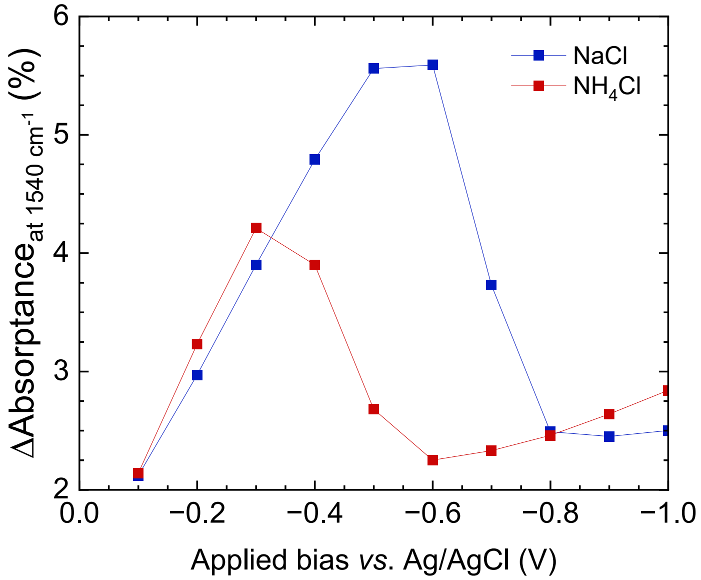

Supplementary Fig. 15 | The ΔAbsorptance at 1540 cm^{-1}, corresponding to the aromatic core breathing mode, recorded at various applied biases *vs* Ag/AgCl for BBL submerged in 0.1 M NH_4Cl and 0.1 M NaCl. This absorption as a function of applied bias follows the conductivity for both electrolytes. The quinoidal ring breathing is thereby associated with mobile charges on the BBL (see also Supplementary Fig. 16). Datapoints are extracted from Supplementary Fig. 11.

Comparing mobile electron absorption in IR spectroscopy

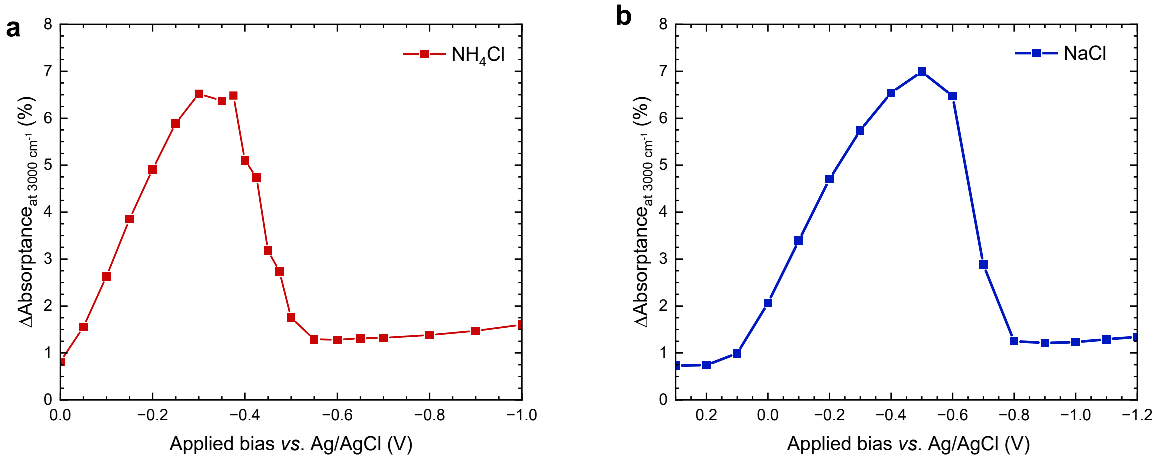

Supplementary Fig. 16 | a,b, The ΔAbsorptance at 3000 cm^{-1}, corresponding to the absorption of mobile charges, recorded at various applied biases *vs* Ag/AgCl for BBL submerged in 0.1 M NH_4Cl (**a**) and 0.1 M NaCl (**b**). The absorption of mobile charges as a function of applied bias follows the conductivity for both electrolytes. Datapoints are extracted from Supplementary Figs. 12 and 13, respectively.

THz conductivity spectra

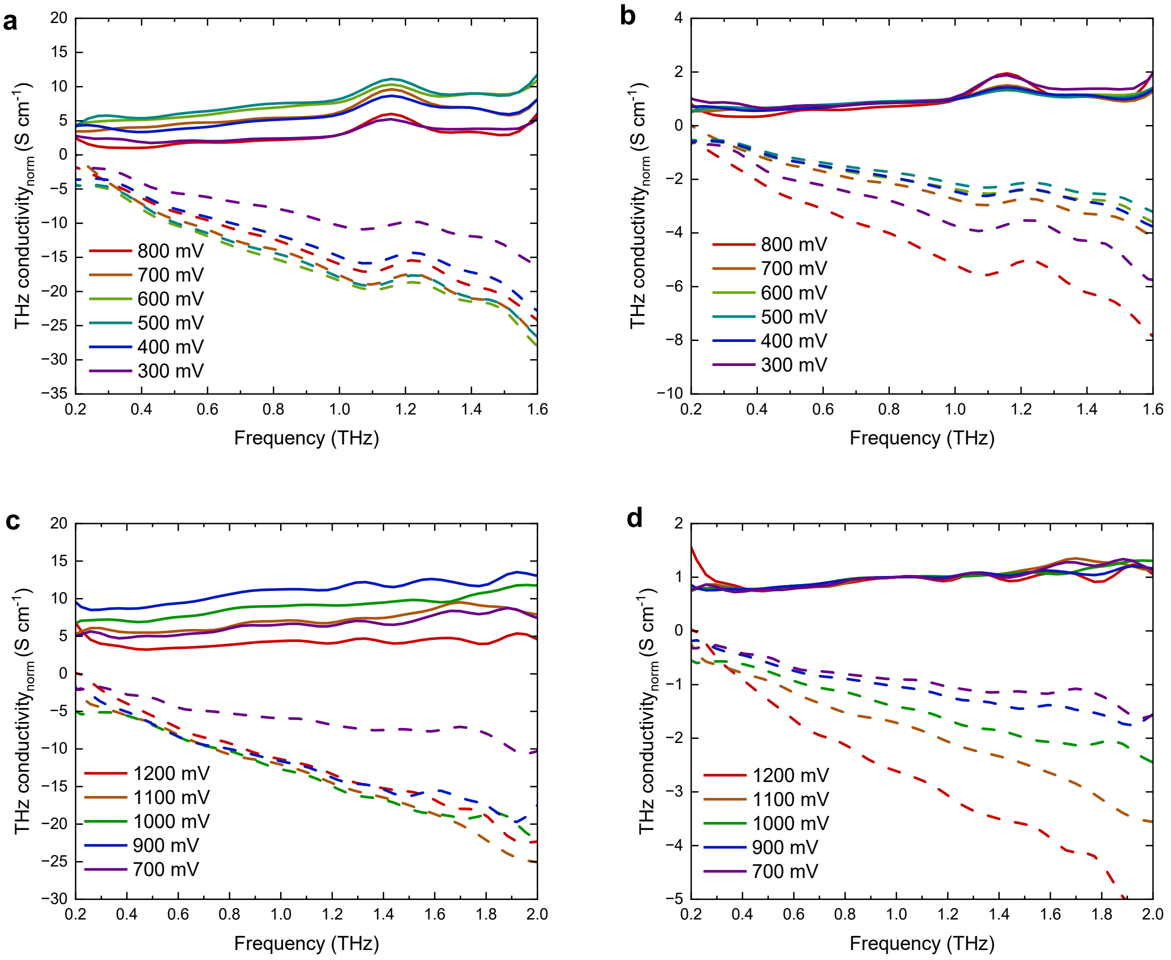

Supplementary Fig. 17 | a-d, The real and imaginary part of the THz conductivity spectra, for several applied biases *vs* Ag/AgCl, recorded for BBL submerged in (**a**,**b**) 0.1 M NH_4Cl and (**c**,**d**) 0.1 M NaCl. The spectra in **b**,**d** are normalized to the real part of the THz spectrum to facilitate comparison of the imaginary part, which relates to the short-range THz effective mobility. In the normalized conductivity spectra, the presence of a more negative imaginary conductivity indicates a higher charge localization and a lower charge mobility.

THz conductivity charge density and localization parameter c_1

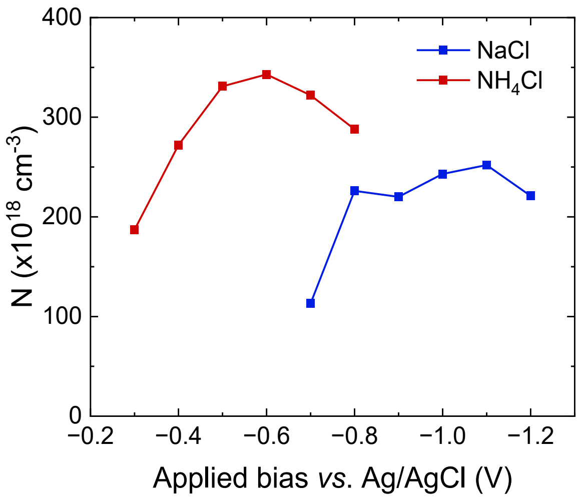

Supplementary Fig. 18 | The charge density of mobile charges extracted from THz spectroscopy as function of applied bias (*vs* Ag/AgCl) for BBL electrochemically doped in 0.1 M NH_4Cl and NaCl.

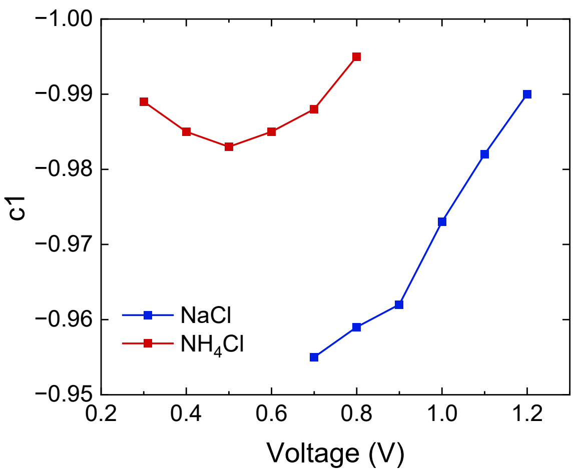

Supplementary Fig. 19 | Localization parameter c_1 as extracted from Drude-Smith (DS) model fittings of the complex THz conductivity spectra for BBL submerged in 0.1 M NH_4Cl (red) or NaCl (blue). The c_1 value closer to -1 is indicative of more localized charge transport, which can be related to several factors such as charge localization, energetic disorder, and stronger charge-ion interactions. From the DS fittings the scattering time is extracted (= 7 fs for both electrolytes). Both fitted parameters are used to extract the THz effective mobility at the different voltages (Fig. 3), where the more negative value of c_1 at higher doping levels underlies the lower charges mobilities.

Molecular dynamics simulations

For doping levels below 150%, the intermolecular distance remains largely insensitive to the nature of the cation (Supplementary Fig. 20a). However, above this threshold, a significant increase in the intermolecular distance is observed in the BBL:NH_4^+ systems. This increase arises from disruptions in the π-stacking of BBL chains caused by the intrusion of ions and water molecules between the chains (Supplementary Fig. 20c). Notably, these disruptions are localized and lead to pronounced increases in the intermolecular distances for a limited number of dimers. Consequently, the average intermolecular distance becomes less representative of the true spacing between π-stacked BBL chains. The evolution of the interlayer distance upon doping follows a similar trend for both cation types (Supplementary Fig. 20b). However, the interlayer distance saturates at a smaller value for the BBL:NH_4^+ system compared to BBL:Na^+. Our simulations further indicate that NH_4^+ exhibits stronger interactions with BBL chains than Na^+. At a 100% doping level, the interaction energy per ion is approximately -50 kcal/mol for the BBL:NH_4^+ system and -40 kcal/mol for the BBL:Na^+ system (Supplementary Figure 20d).

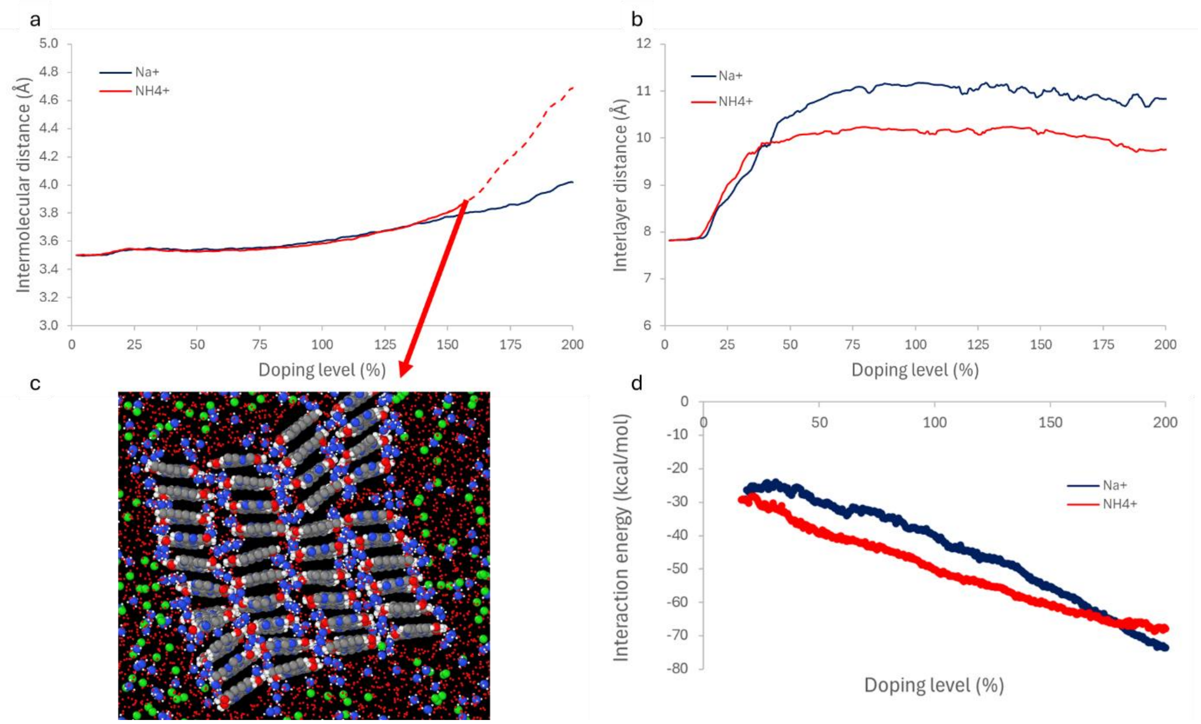

Supplementary Fig. 20 | a-b, Evolution of the intermolecular (**a**) and interlayer (**b**) distances in BBL:ions systems upon doping. The doping level refers to the number of electrons per repeat unit of BBL, with 200% representing the fully doped state with 2 electrons per repeat unit[1]. **c**, Representation of the BBL:NH_4^+ system at 160% doping level. **d**, Evolution of the interaction energies between the cations inserted in the central interlayer regions and the two central BBL layers. The reported interaction energies are divided by the number of inserted ions for easier comparison.

IR of methylated ammonium cations

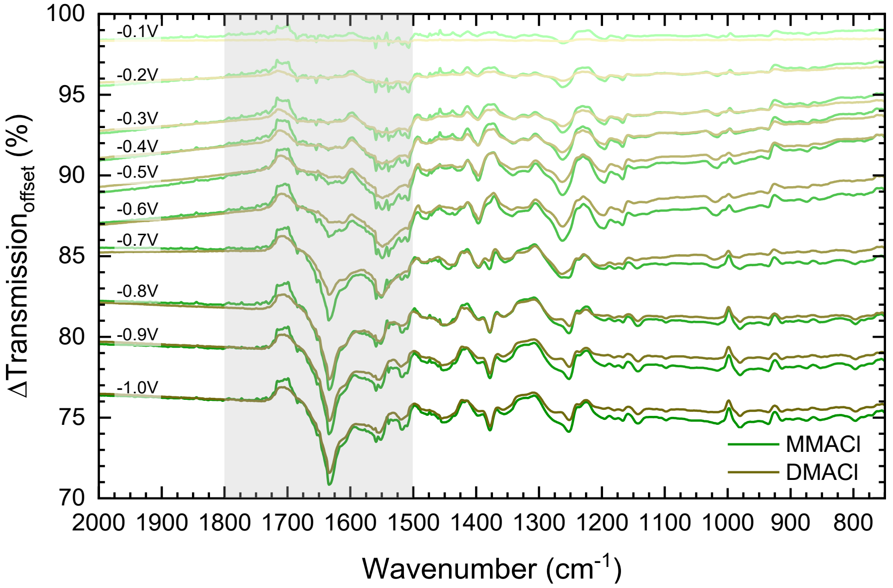

Supplementary Fig. 21 | The fingerprint region of the differential transmittance spectra from operando IR spectroscopy of BBL doped at -0.1 V to -1.0 V while submerged in 0.1 M MMACl and 0.1 M DMACl. Both electrolytes exhibit similar spectra to NH_4Cl, but with a distinct potential shift corresponding to their pK_a. Spectra as a function of bias are offset for clarity, and the spectral region of the carbonyl vibration, featured in Fig. 3a of the main text for NaCl and NH_4Cl, is highlighted in grey.

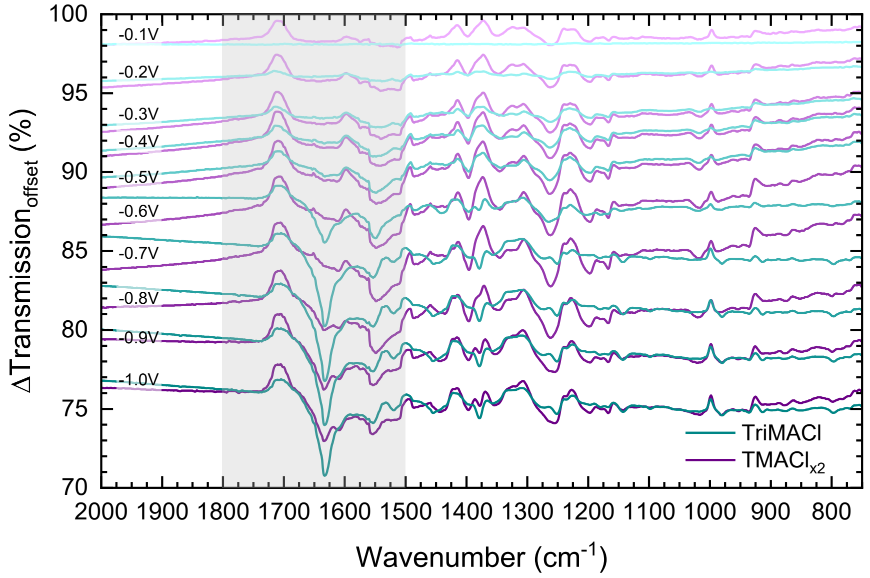

Supplementary Fig. 22 | The fingerprint region of the differential transmittance spectra from operando IR spectroscopy of BBL doped at -0.1 V to -1.0 V while submerged in 0.1 M TriMACl and 0.1 M TMACl. The spectra of TMACl are multiplied by 2 since TMACl only covered half of the IR substrate. TriMACl exhibits similar spectra to NH_4Cl, with a distinct potential shift corresponding to its pK_a, while TMACl displays virtual identical spectra to NaCl. Spectra as a function of bias are offset for clarity, and the spectral region of the carbonyl vibration, featured in Fig. 3a of the main text for NaCl and NH_4Cl, is highlighted in grey.

EQCM-D of methylated ammonium cations

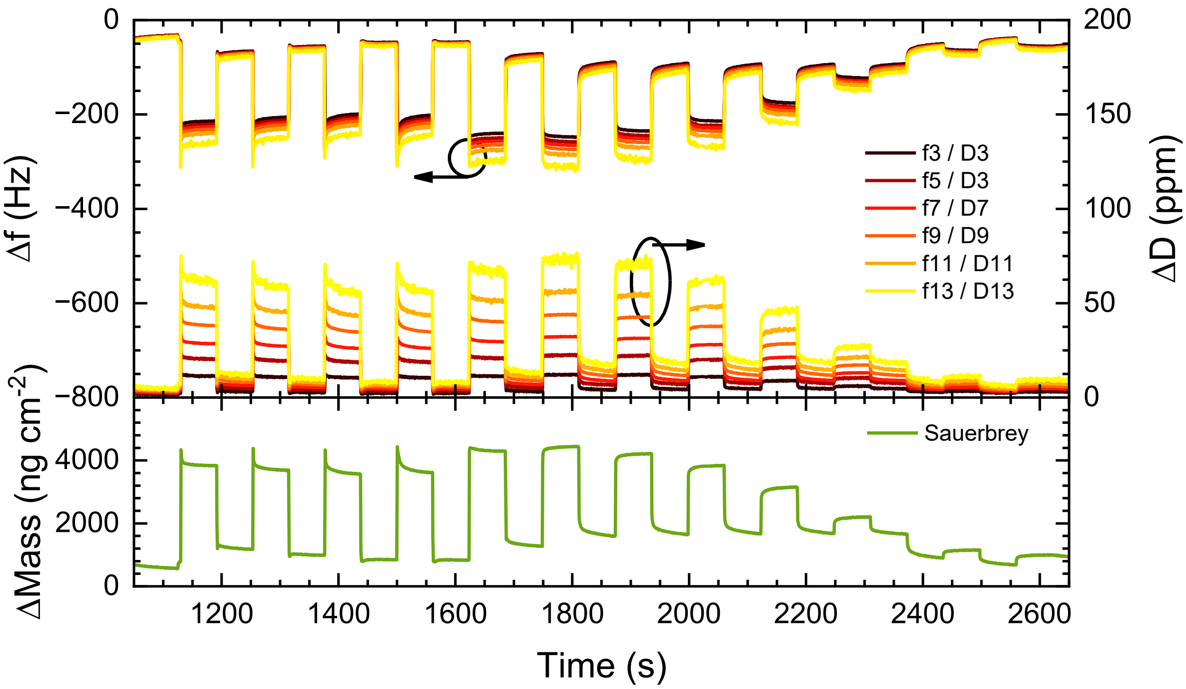

Supplementary Fig. 23 | The measured Δf (left axis) and ΔD (right axis) for the 3rd-13th overtone from EQCM-D measurements on BBL submerged in 0.1 M MMACl. The applied bias is decreased stepwise from -1.0 V to +0.2 V *vs* Ag/AgCl with intermittent 0 V steps. Bottom panel includes the Δmass from the Sauerbrey equation relative to the mass of the pre-cycled BBL film. The mass response as function of bias shows a local maximum, similar to NH_4Cl but at a shifted applied bias.

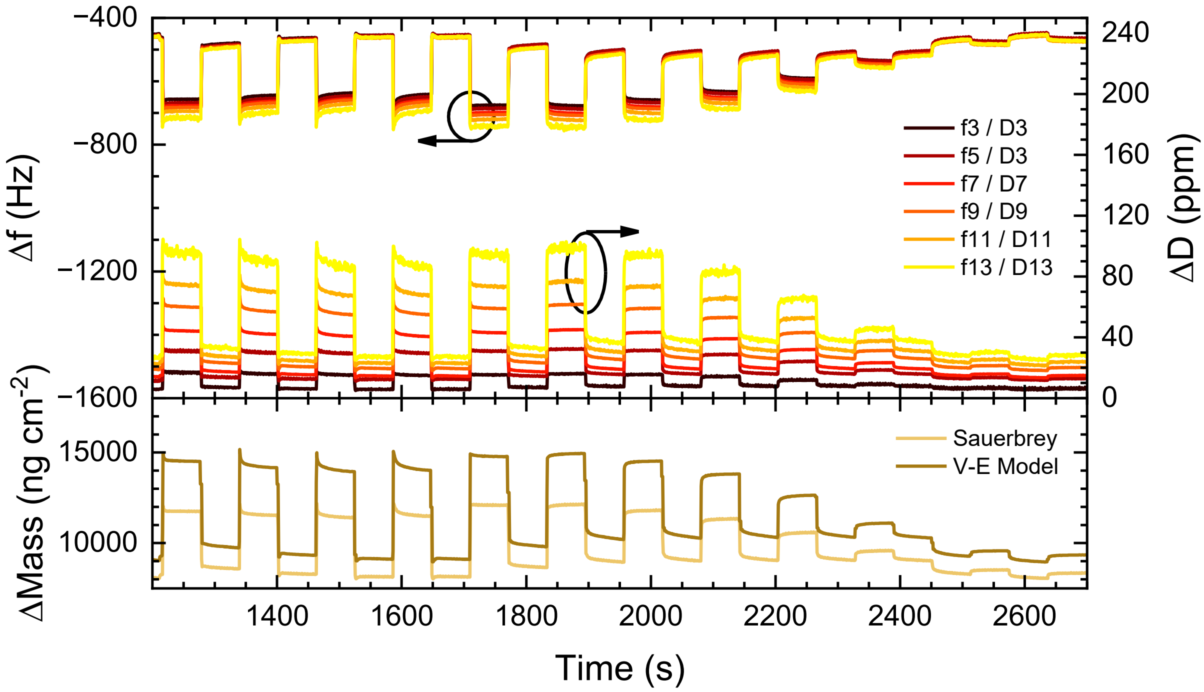

Supplementary Fig. 24 | The measured Δf (left axis) and ΔD (right axis) for the 3rd-13th overtone from EQCM-D measurements on BBL submerged in 0.1 M DMACl. The applied bias is decreased stepwise from -1.0 V to +0.2 V *vs* Ag/AgCl with intermittent 0 V steps. Bottom panel includes the Δmass from the Sauerbrey equation and visco-elastic (V-E) modeling relative to the mass of the pristine dry BBL film. The mass response as function of bias shows a local maximum, similar to NH_4Cl but at a shifted applied bias.

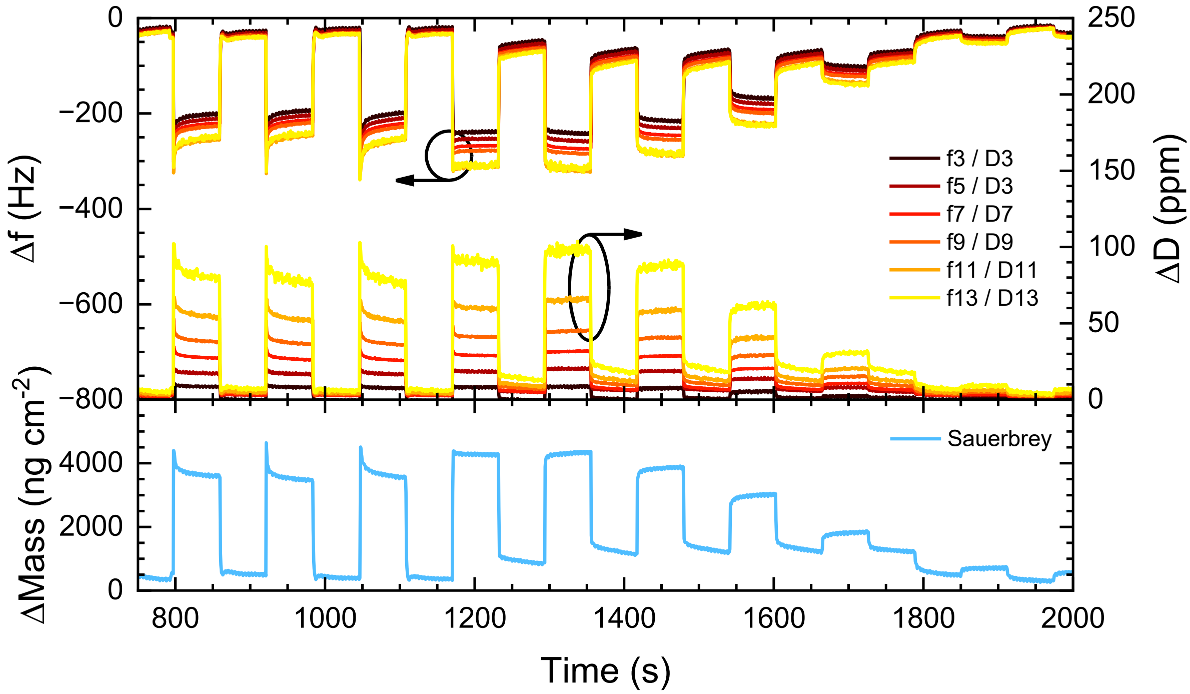

Supplementary Fig. 25 | The measured Δf (left axis) and ΔD (right axis) for the 3rd-13th overtone from EQCM-D measurements on BBL submerged in 0.1 M TriMACl. The applied bias is decreased stepwise from -0.8 V to +0.2 V *vs* Ag/AgCl with intermittent 0 V steps. Bottom panel includes the Δmass from the Sauerbrey equation relative to the mass of the pre-cycled BBL film. The mass response as function of bias shows a local maximum, similar to NH_4Cl but at a shifted applied bias.

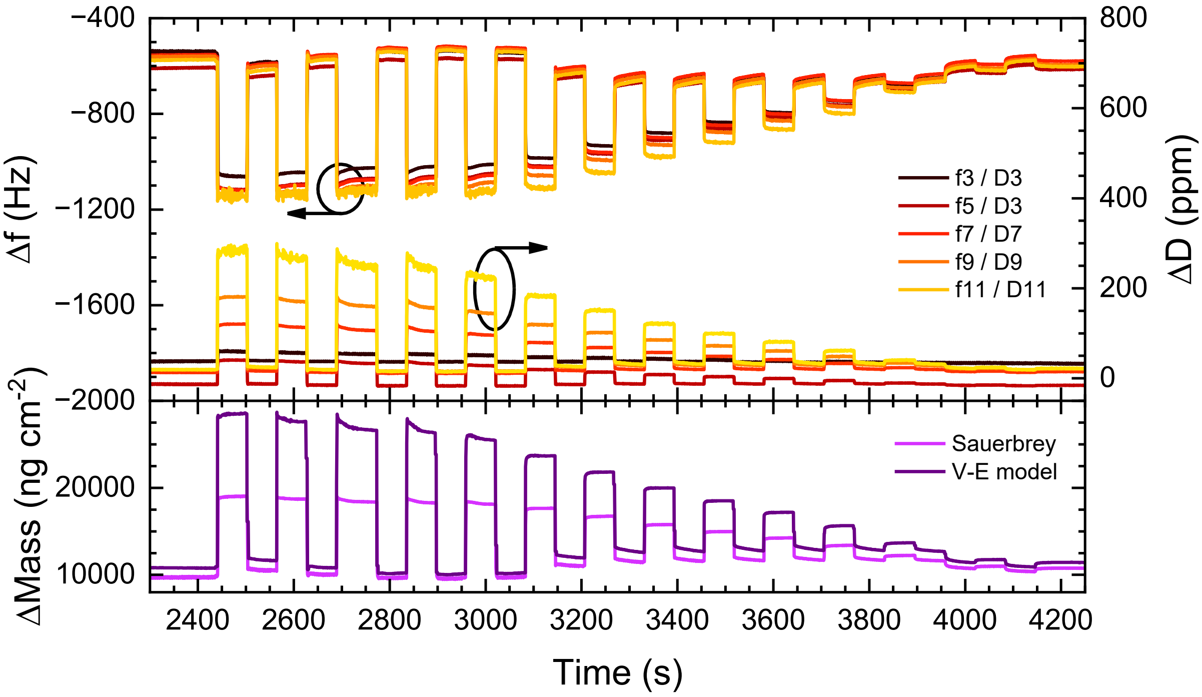

Supplementary Fig. 26 | The measured Δf (left axis) and ΔD (right axis) for the 3rd-11th overtone from EQCM-D measurements on BBL submerged in 0.1 M TMACl. The applied bias is decreased stepwise from -1.0 V to +0.2 V *vs* Ag/AgCl with intermittent 0 V steps. Bottom panel includes the Δmass from the Sauerbrey equation and visco-elastic (V-E) modeling relative to the mass of the pristine dry BBL film. The mass response as function of bias shows a semi-continuous increase with increased applied bias, similar to NaCl.

Mass differences from EQCM-D on the same BBL film

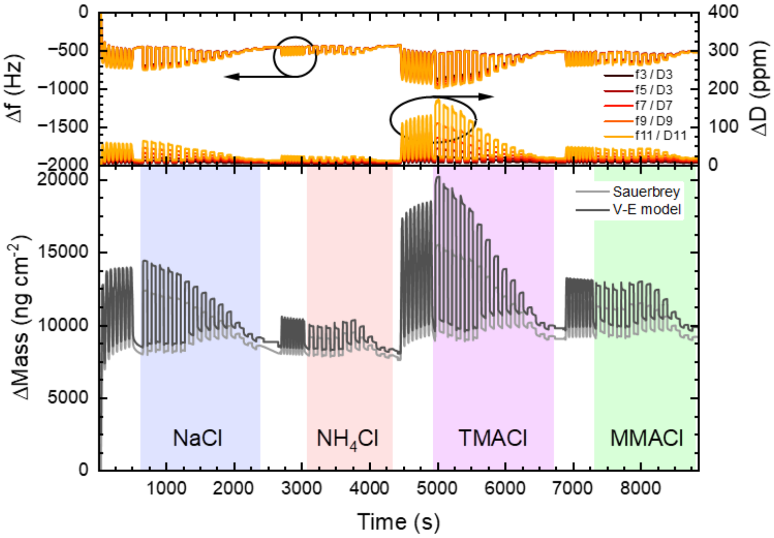

Supplementary Fig. 27 | The measured Δf (left axis) and ΔD (right axis) for the 3rd-11th overtone from EQCM-D measurements on a single BBL film submerged in 0.1 M solution of subsequently NaCl, NH_4Cl, TMACl, and MMACl. Upon introduction of each electrolyte, the film is cycled 8 times in CV followed by a stepwise decrease in applied bias from -1.2 V (NaCl and TMACl), -1.0 V (MMACl), or -0.8 V (NH_4Cl) to +0.2 V *vs* Ag/AgCl with intermittent 0 V steps. Bottom panel includes the Δmass from the Sauerbrey equation and visco-elastic (V-E) modeling relative to the mass of the pristine dry BBL film. The decreasing bias steps are color-coded for each electrolyte: NaCl (red), NH_4Cl (blue), TMACl (purple), and MMACl (green).

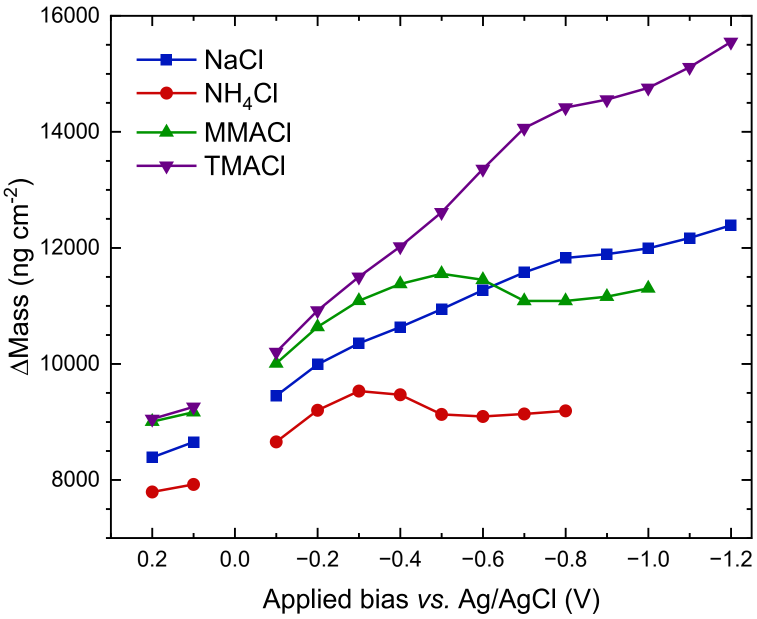

Supplementary Fig. 28 | Summary of the Δmass from EQCM-D at each voltage step (from the Sauerbrey equation) for 0.1 M NaCl, NH_4Cl, TMACl and MMACl. Datapoints are collected from the measurement displayed in Supplementary Fig. 27. We find that methylation increases the Δmass significantly when comparing NH_4Cl and MMACl. TMACl has a significantly higher Δmass than any of the other electrolytes.

pK_a of different ammonium cations

Supplementary Table 1 | The pK_a of the different ammoniumchloride salts used in this work.

Name (acronym)	pK_a	Reference
Ammoniumchloride (NH_4Cl)	9.25	2
Methylammonium chloride (MMACl)	10.63	3
Dimethylammonium chloride (DMACl)	10.78	3
Trimethylammonium chloride (TriMACl)	9.80	3

CV and pH on the methylated ammonia

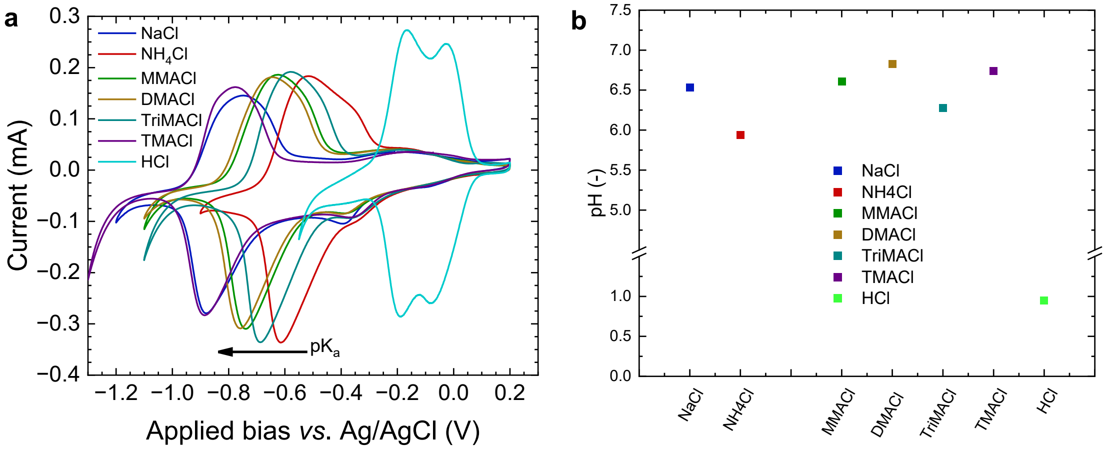

Supplementary Fig. 29 | a, Cyclic voltammetry measured in 0.1 M electrolyte for various methylated ammonium cations, including NaCl and HCl for reference, measured *vs* Ag/AgCl at a scan rate of 0.05 V/s. A distinct shift to higher applied bias is observed with increasing pK_a of the cation. **b**, Measured pH of the employed electrolytes. The measured pH shows small changes except for HCl.

BBL transfer curve stability

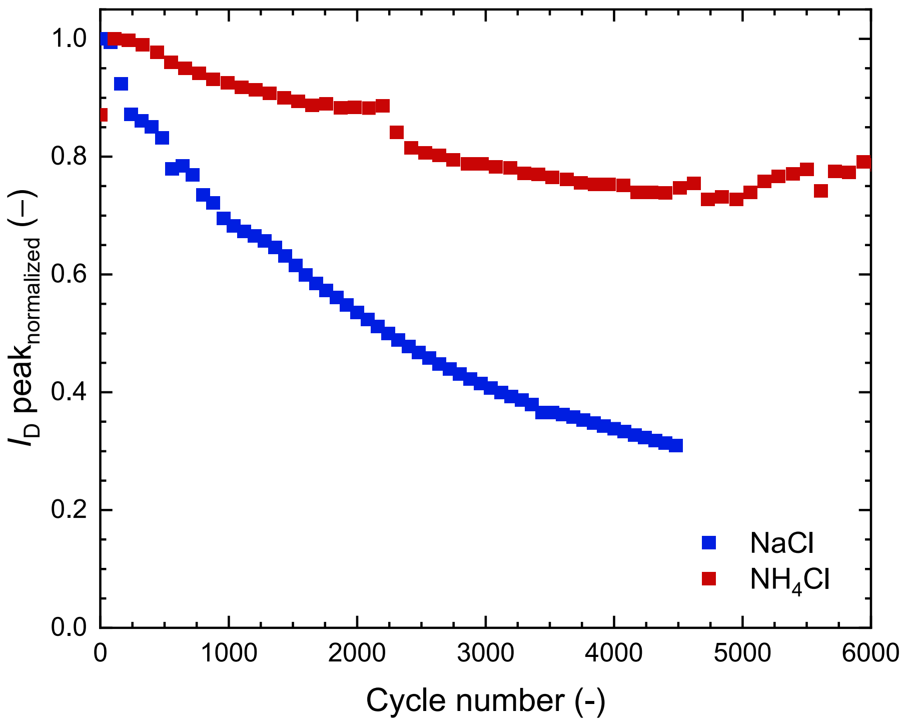

Supplementary Fig. 30 | Normalized peak drain current (I_d peak) vs. cycle number during repeated transfer curve measurements in 0.1 M NH_4Cl or NaCl. Gate bias is cycled from +0.1 V to -0.8 V (NH_4Cl) or -1.1 V (NaCl) vs Ag/AgCl. Drain voltage is 0.3 V. Gate bias limits were chosen to avoid side reactions.

Operando GIWAXS cell

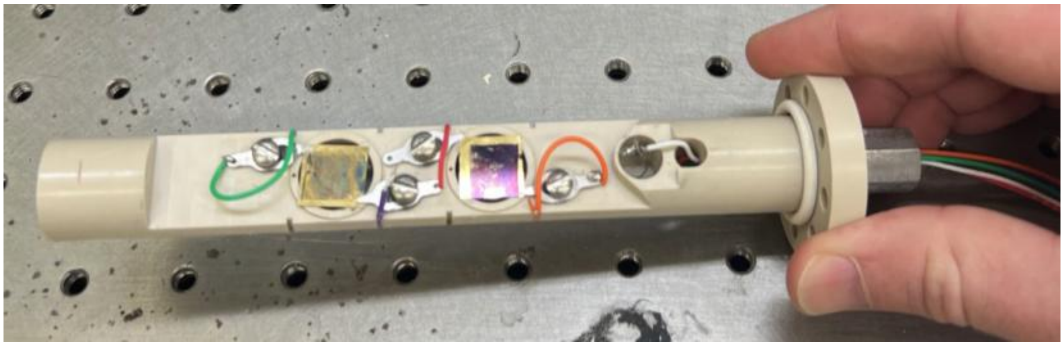

Supplementary Fig. 31 | Picture of the electrochemical operando GIWAXS cell. This cell can hold two different substrates. The electrolyte sits on the bottom (the interior part is hollow) and reference and counter electrodes are sitting in the small well on the right. This part sits in a sealed chamber kept in humid nitrogen atmosphere, inside a larger vacuum chamber.

Molecular dynamics analysis regions

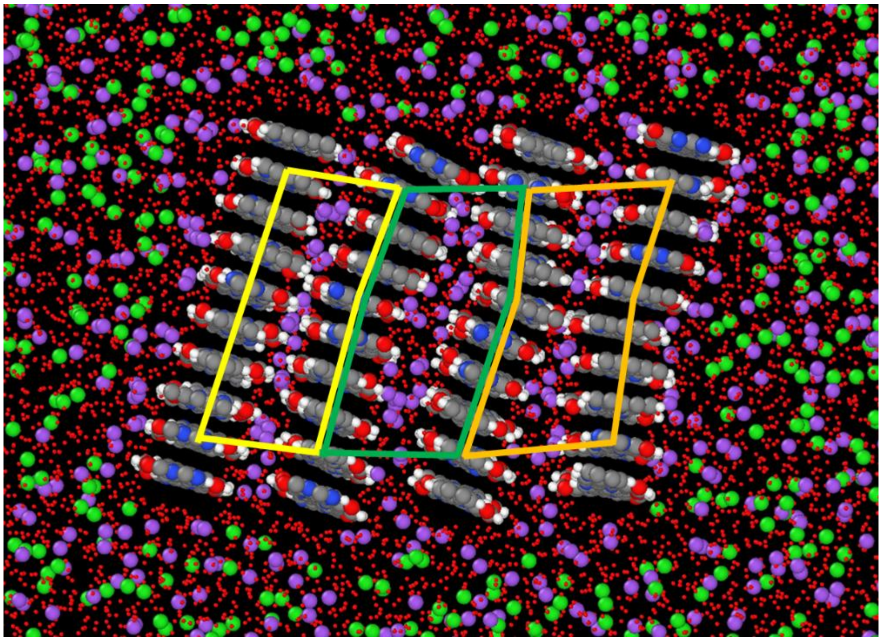

Supplementary Fig. 32 | Schematic representation of the different regions defined for the analysis of MD results.

Supplementary Note 1: (Operando) GIWAXS on BBL

Dry *vs* wet films

We compared the GIWAXS patterns of BBL dry film and BBL wetted by 0.1 M NaCl and 0.1 M NH_4Cl (Supplementary Fig. 33). The dry film was prepared on a Si substrate, while the wet films were transferred onto gold-coated Si frit cells (Supplementary Fig. 31). The wet films were further subjected to electrochemical impedance spectroscopy prior to the measurement (10 mV around the open circuit potential) to test the connections for subsequent electrochemical measurements.

In general, both wet films exhibit a weakening of crystalline features, which can only in part be attributed to the slightly different experimental setup. This suggests that BBL loses crystallinity in the wet state. Our results somewhat contradict the findings by Guo et al.[4], which showed a minor loss of crystallinity upon contacting with water concomitant with a small increase in height from AFM. In their work, however, the employed BBL had a particularly high molecular weight. We attribute the observed differences to the molecular weight of BBL used here and the prior electrochemical impedance measurements that we performed prior to the GIWAXS measurements to ensure working electrical connections.

Regardless, it is possible to identify the lamellar peak in the out-of-plane (oop) direction and the π-stacking peak in the in-plane (ip) direction. The most significant change (Supplementary Table 2) is observed in the lamellar (100) peak position: for the dry film, the d-spacing in the 100 direction is 8.7 Å, whereas in the wet film, the crystal structure expands to 12.6 Å and 13.1 Å for films soaked in NaCl and NH_4Cl, respectively. Minimal changes are noted for the π-stacking peak. The lattice parameters for the dry film are consistent with those reported in previous studies[4,5], while the lamellar spacings in the wet films are larger than those documented previously[4]. Another peak is visible in the oop direction around $q = 1.6$ Å^{-1}. This peak has been attributed to second-order lamellar scattering (200) but, in our measurement, this peak does not lie at double the q value of the 100 peak.

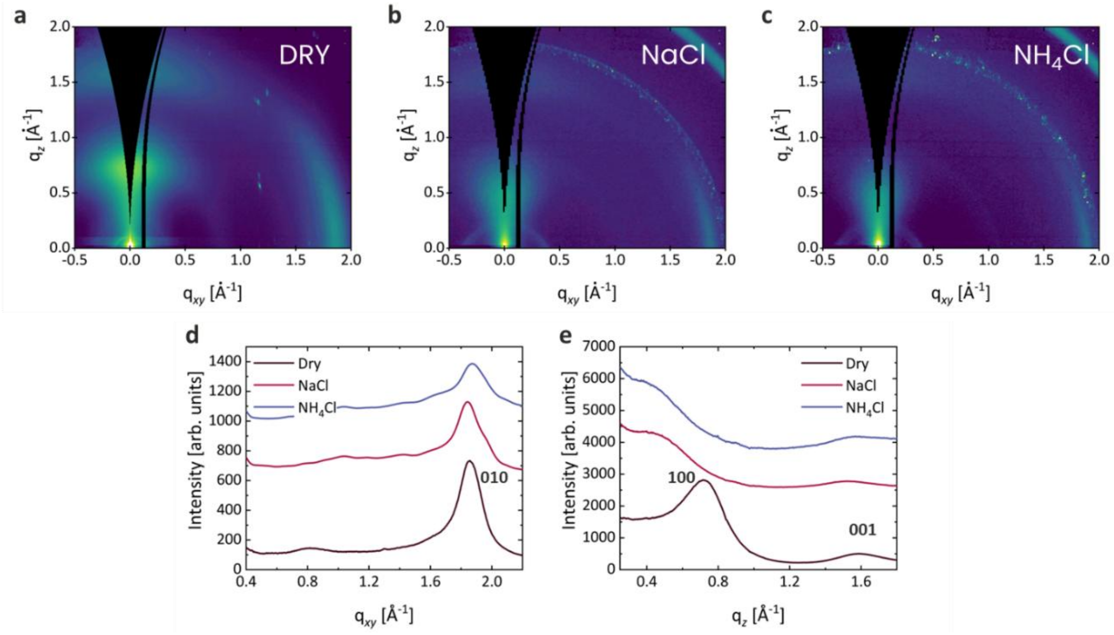

Supplementary Fig. 33 | a-c. 2D GIWAXS patterns of BBL films in dry state (**a**) and soaked in NaCl (**b**) and NH_4Cl (**c**). **d-e**, In-plane (**d**) and out-of-plane (**e**) X-ray scattering linecuts. GIWAXS linecuts are offset for clarity and peak labels are shown on the bottom trace.

Supplementary Table 2 | Lattice parameters for 100 and 010 peaks found by fitting the peaks with gaussians curves.

	Dry	NaCl	NH_4Cl
Out of plane (100)			
d-spacing [Å]	8.7	12.6	13.1
coherence length [Å]	24.9	22.7	25.9
In plane (010)			
d-spacing [Å]	3.38	3.39	3.34
coherence length [Å]	32.6	33.3	38.6

Electrochemical operando GIWAXS

We then performed electrochemical operando GIWAXS measurements on BBL thin films immersed in NaCl and NH_4Cl. Scattering images were acquired while running cyclic voltammetry (CV) on the polymer films. In this setup, the CVs (Supplementary Figs. 34 and 35) are not ideal, possibly due to poor contact between the gold electrode and the BBL film. Nevertheless, the polymer films did get charged, allowing us to observe changes in the microstructure (Supplementary Figs. 36 and 37).

Doping induces a general shift of lattice parameters toward higher q values, corresponding to smaller lattice distances, in both the lamellar and π-stacking peaks, although these shifts are relatively small. The other out-of-plane peak also exhibits a similar behavior. This peak is often referred to as the 200 peak; however, it cannot be assigned to this reflection, as it does not appear at twice the distance of the 100 reflection. We hypothesize it might be a π-stacking signal arising from the face-on fraction of BBL, which is usually less evident in dry films, or it could be something else entirely.

For both electrolytes, a signal at smaller q (~0.3 $Å^{-1}$) rises with doping (Supplementary Figs. 36a,b and 37a,b). This is not a well-defined peak, and it could be related to an increase in diffuse scattering from the polymer film due to swelling or longer-range order over distances of 2–3 nm. This feature is more evident for the film in NaCl.

To perform a deeper analysis, we examined the dynamic variation of lattice parameters versus the applied bias. To determine the peak center positions, and hence the lattice spacings, the peaks were fitted using Gaussian functions. It should be noted that the identification of these peaks, particularly the 100 peak, is influenced by the definition of the background, which is somewhat arbitrary due to the diffuse scattering lacking a well-defined shape. Consequently, while the absolute values derived from these fits may not be entirely reliable, the observed trends are consistent and trustworthy. The results of the fittings are reported in Supplementary Figs. 38 and 39.

For both electrolytes, the most significant changes are observed for the 100 peak, where the d-spacing goes from 10.0 Å to 9.2 Å in NaCl and from 10.7 Å to 10.4 Å in NH_4Cl, with the smallest value reached at higher doping conditions (Supplementary Figs. 38a and 39a). These values are smaller than those reported in Supplementary Table 1, likely due to a combination of improved packing after a couple of CV cycles, compression due to cation injection around the crystallite, and possible fitting artifacts. Minimal changes

are observed in the position of the π-stacking peak, with a d-spacing modulation on the order of 0.01 Å (Supplementary Figs. 38b and 39b). The other out-of-plane peak shows a d-spacing change from 3.7 Å to 3.6 Å in NaCl and from 3.605 Å to 3.575 Å in NH_4Cl, with the smallest value reached at higher doping conditions (Supplementary Figs. 38c and 39c).

In conclusion, the significant weakening of the scattering signal compared to the dry film, combined with the limited peak position modulation and rise of diffuse scattering signal at small q values, suggests that BBL films lose crystallinity upon doping in aqueous electrolytes. As a consequence, a large fraction of the film will be amorphous and counterion plus water uptake will mostly impact this amorphous fraction of the material, which is not probed by GIWAXS.

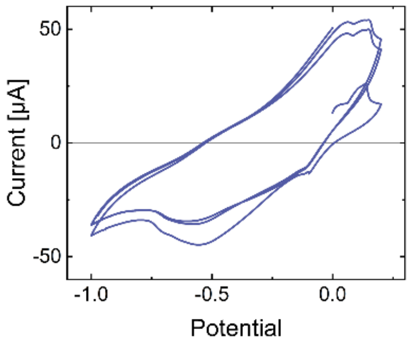

Supplementary Fig. 34 | Cyclic voltammetry of BBL in NH_4Cl 0.1 M (scan rate 10 mV s^{-1}, bias in V *vs* Ag/AgCl), run in the operando GIWAXS cell.

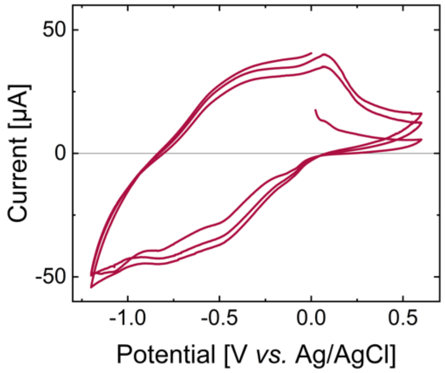

Supplementary Fig. 35 | Cyclic voltammetry of BBL in NaCl 0.1 M (scan rate 10 mV s^{-1}), run in the operando GIWAXS cell.

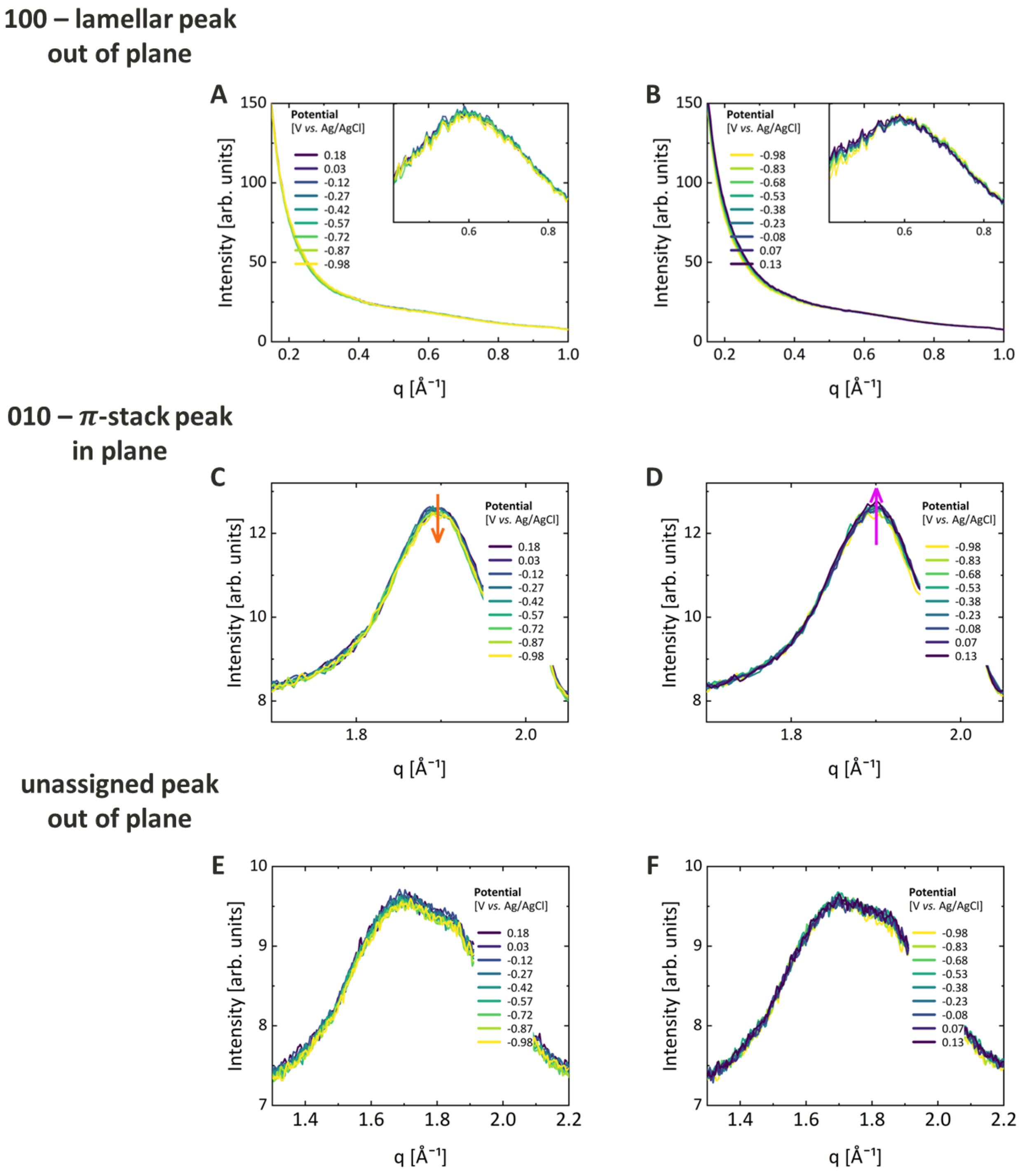

Supplementary Fig. 36 | a-f, Linecuts of the lamellar (**a**, **b**), π-stacking (**c**, **d**) and other unassigned peak (**e**, **f**) peak of BBL during the forward (**a**, **c**, **e**) and backward (**b**, **d**, **e**) scan of the 2nd cycle of the CV in NH_4Cl. Insets in (**a**, **b**) highlight the lamellar peak and are obtained after subtraction of the diffuse scattering component.

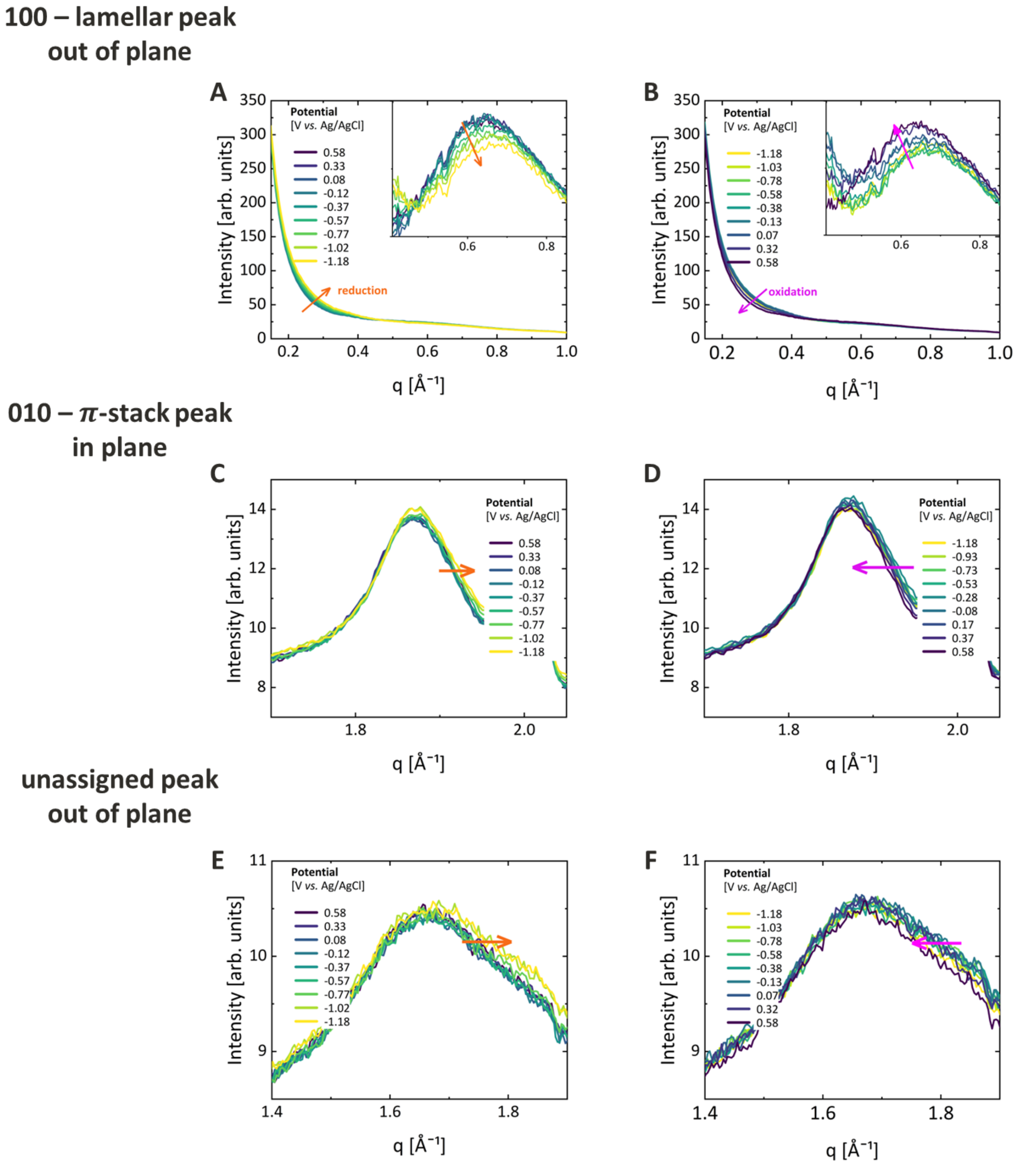

Supplementary Fig. 37 | a-f, Linecuts of the lamellar (**a**, **b**), π-stacking (**c**, **d**) and other unassigned peak (**e**, **f**) peak of BBL during the forward (**a**, **c**, **e**) and backward (**b**, **d**, **e**) scan of the 2nd cycle of the CV in NaCl. Insets in (**a**, **b**) highlight the lamellar peak and are obtained after subtraction of the diffuse scattering component.

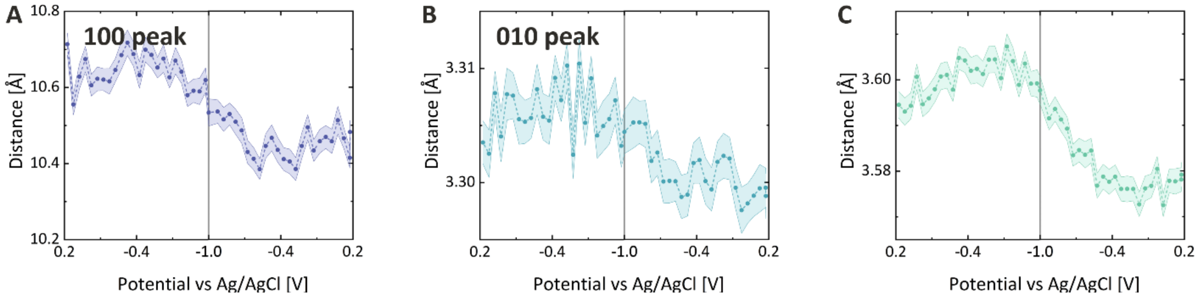

Supplementary Fig. 38 | a-c, Fit results from X-ray scattering peaks of BBL in NH_4Cl 0.1 M. Lattice distance of the lamellar (**a**), π-stacking (**b**) and other unassigned peak (**c**) as a function of the applied potential. Plots show fit results. The colored area represents the standard error of the fit and the points represent the fitted value.

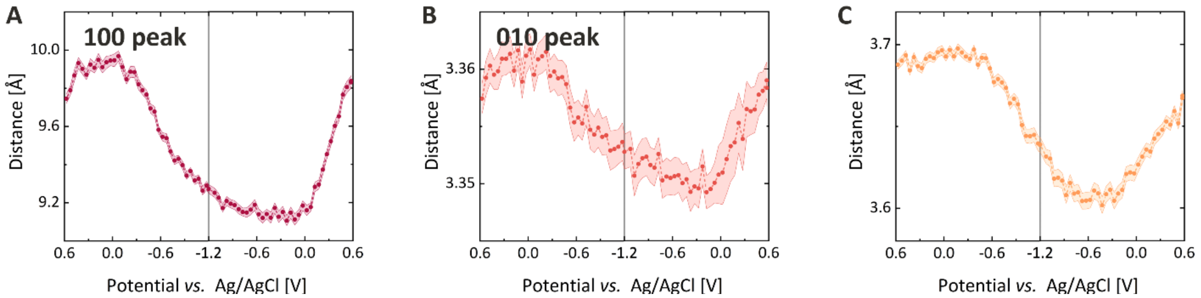

Supplementary Fig. 39 | a-c, Fit results from X-ray scattering peaks of BBL in NaCl 0.1 M. Lattice distance of the lamellar (**a**), π-stacking (**b**) and other unassigned peak (**c**) as a function of the applied potential. Plots show fit results. The colored area represents the standard error of the fit and the points represent the fitted value.

Supplementary Note 2: ^{2}H NMR

Nuclear magnetic resonance (NMR) spectroscopy is a quantitative, nucleus-specific method that provides insights into both local structure and dynamics within materials. Numerous NMR studies have been used to study the structure of a polymer,[6-8] the environment and motion of ions in a polymer film,[9,10] and the behaviors of water in polymer membranes[11,12,13]. When studying water, deuterium NMR presents an effective experimental approach for characterizing water molecules with restricted motion. This is due to the quadrupolar interaction of the spin-1 ^{2}H nucleus in D_2O, which is sensitive to the local electric field gradient (EFG) at the nucleus. This orientation of the EFG tensor (with principal component V_{ZZ}, Supplementary Fig. 40a) is dominated by the orientation of the O-D bond and its size will be modified by the extent of hydrogen bonding[14]. In free deuterated water, the rapid isotropic motion of the water results in a zero, time-averaged value of the electric field gradient and thus a removal of the quadrupolar interaction (*i.e.*, complete averaging to zero, Supplementary Fig. 40b). When water is constrained in an anisotropic medium, be it a pore, a vesicle or even a cell[15], while rapid motion may still occur, the time-average of the electric field gradient may no longer be zero. This results in incomplete averaging of the quadrupolar interaction (Supplementary Fig. 40c). This partial-averaging produces small residual quadrupolar couplings, distinct from the complete-averaging observed in isotropic water, which reflect the extent of anisotropy of the medium. Residual quadrupolar coupling is an important anisotropic NMR parameter detectable only in nuclei with spin quantum number $I>1/2$, such as ^{2}H, but not ^{1}H. ^{2}H NMR has been employed to study a range of hydrated materials by leveraging the sensitivity of quadrupolar interactions, including cartilage[16,17], collagens[18,19], hydrogels and polymers[20]. Thus, deuterium NMR spectroscopy is a valuable tool for investigating water interactions with macromolecules.

In this study, deuterated aqueous electrolytes were used to distinguish between free water and water molecules adsorbed within the BBL polymer film, leveraging the quadrupolar properties of ^{2}H nuclei. Operando NMR, in particular, enables real-time observation of individual nuclear species within an active device during electrochemical processes. Therefore, operando ^{2}H NMR spectroscopy is employed here as a non-destructive and quantitative technique to track water movement within the BBL film during electrochemical doping and dedoping. Although BBL films are inherently hydrophobic, electrochemical doping in aqueous electrolyte induces swelling of the BBL film, which enables water molecules to be transported into both isotropic and anisotropic regions of BBL films (Supplementary Fig 40b,c)[4]. In the ordered domains (Supplementary Fig. 40c), water molecules interact with the polymer matrix, resulting in constrained motion and partial alignment due to the local ordering of the BBL polymer chains with respect to the film substrate. The EFG tensor is partially averaged by the fast reorientation of D_2O molecules. For example, for rapid rotation about D_2O's C_2 symmetry axis, an axially symmetric tensor results with a principal component V_{ZZ} aligned along the C_2 axis[21]. More generally, the principal component V_{ZZ} can be thought of as the average orientation of the O-D bond of a water molecule, which is not random for water in the ordered polymer regions. The anisotropic motion of water subsequently generates a non-zero average of the quadrupolar interaction, which leads to a characteristic doublet lineshape corresponding to the two transitions, $|+1\rangle - |0\rangle$ and $|-1\rangle - |0\rangle$ (Supplementary Figure 40d). Conversely, in the disordered regions (Supplementary Fig. 40c), although the ^{2}H nuclei still interact with local EFGs, the motion of water between sites in between the randomly oriented polymer chains causes these interactions to average out, producing no splitting.

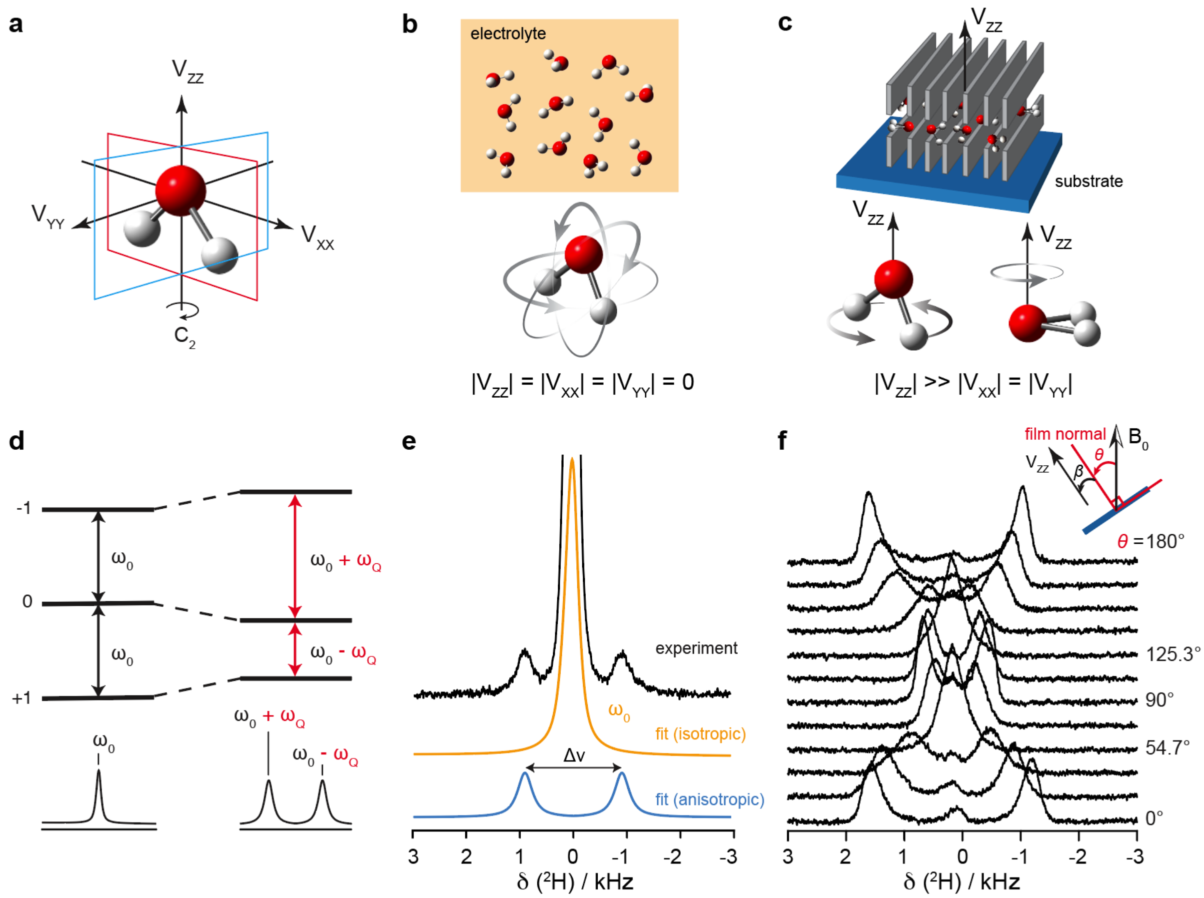

Supplementary Fig. 40 | a-c, Schematic illustration of a D_2O molecules with its local EFG tensors. The time-averaged direction of the principal component of the ^2H quadrupolar tensor in an D_2O molecule is either (**b**) randomly oriented in the bulk electrolyte or (**c**) oriented between the polymer layers. (**b**) For free D_2O molecules, the EFG tensors are completely averaged to zero by isotropic rotational and translational motion. (**c**) For confined D_2O molecules, the EFG tensors are only partially averaged due to restricted molecular motion within the layers, which results in a non-zero principal component V_{ZZ} that is oriented normal to the substrate. **d**, Zeeman splitting diagram for a spin-1 ^2H nucleus, where the first-order quadrupolar interaction results in two non-degenerate observable transitions, differing by +/- ω_Q, where ω_Q is the quadrupolar coupling frequency. **e**, Experimental data and fit of the ^2H NMR spectrum of a BBL film soaked in 0.1 M NaCl in D_2O with two components: a singlet peak assigned to D_2O in the aqueous NaCl solution ("isotropic", orange) and a doublet peak separated by the quadrupolar splitting frequency (Δν) which is assigned to D_2O inside the BBL film ("anisotropic", blue). **f**, The effect of film orientation with respect to the static magnetic field B_0 on the ^2H NMR spectra where θ is the angle between the film normal (red) and B_0 in the lab frame. The angle β is defined in the molecular frame as the angle between the principal component V_{ZZ} and the film normal. Note that the singlet water signal at 4.7 ppm (equivalent to about 0.22 kHz at a 7.05 Tesla magnetic field) in (**f**) is much weaker than in (**e**) due to the removal of the soaked separator from the operando cell, undertaken to improve resolution of the doublet splitting at certain film orientations.

The ^{2}H NMR spectrum of an operando cell containing BBL film soaked in 0.1 M NaCl in D_2O is shown in Supplementary Fig. 40e. Three NMR signals are observed, comprising two components: one singlet and one doublet. The singlet is assigned to the free water molecules in the excess NaCl electrolyte outside of the BBL film and possibly from water in isotropic regions of the BBL film. The doublet, with a quadrupolar splitting ($\Delta\nu = 2\,\omega_Q$) is characteristic of confined D_2O molecules, where molecular motion is constrained. This residual quadrupolar coupling clearly indicates that the film contains ordered anisotropic regions or domains, and water molecules in the anisotropic domains exhibit partial ordering, sharing a common orientation relative to the external magnetic field (B_0). To investigate how the local anisotropic domains are orientated with respect to the film substrate, a piece of hydrated BBL film was rotated inside the NMR detection coil about the coil axis. The film orientation (θ) is defined by the angle between the normal of the film and the external magnetic field B_0 (Supplementary Fig. 40f) in the lab frame. To describe the polymer alignment within the film substrate, an angle β is defined in the molecular frame as the angle between the principal component V_{ZZ} and the film normal. The observed quadrupolar splitting ($\Delta\nu$) exhibits a strong dependence on the film orientation (θ). Since a maximum in splitting is obtained for $\theta = 0°$, and a minimum for $\theta = 54.7°$, this indicates that the principal component V_{ZZ} is also aligned with the film normal ($\beta = 0°$), giving rise to the expression[22]:

$$\Delta\upsilon = \frac{1}{2}\,\chi\,|3\,cos^2\theta - 1| \qquad \text{Equation 1}$$

where χ is the residual quadrupolar coupling constant of the anisotropic water molecules that give rise to the doublet. This equation implicitly assumes that the quadrupolar tensor is axial. While the fit to the spectra with this equation is not perfect (Supplementary Fig. 41a), which suggests a small deviation from axial symmetry and also a distribution of quadrupolar coupling constants likely due to disorder within the film, further analysis of these tensors would require the development of a more detailed model beyond the scope of this work. The most important point that emerges from this rotation plot is that the principal component of the time-averaged value of the electric field gradient (V_{ZZ}) and thus quadrupolar tensor is oriented perpendicular (normal) to the film. If we assume that the anisotropy is caused by rapid motion within a two-dimensional plane or constrained within two parallel plates, this indicates that the domains that cause this anisotropy, as probed by the water, are oriented normal to the film, as shown schematically in Supplementary Fig. 40c. We note that the ^{2}H quadrupole coupling constant of a static water molecule is approximately 180-200 kHz[23], so the residual value extract here of 1-3 kHz implies considerable motion and disorder. Nonetheless, the non-zero quadrupole indicates that the ordered regions that contain the water are, at least to some degree, ordered with respect to the substrate.

Operando ^{2}H NMR experiments on BBL films (Fig. 2) were conducted in deuterated 0.1 M NaCl and NH_4Cl electrolytes to monitor the water movement while running cyclic voltammetry (CV). The film was orientated such that the normal to the film was aligned parallel to the magnetic field (θ = 0º), enhancing peak separation for operando ^{2}H NMR experiments. The electrochemical responses were slightly sluggish (Supplementary Fig. 41b,c) compared to regular CV measurements (Supplementary Fig. 1c), which is expected given the thicker BBL film used for operando NMR measurements.

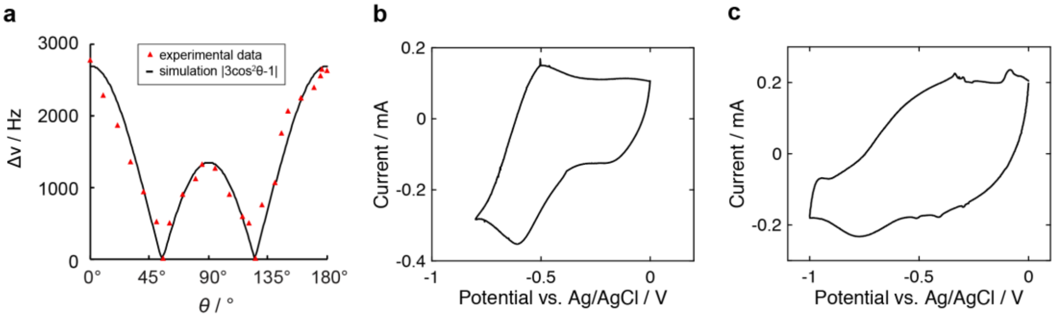

Supplementary Fig. 41 | a, The fitting of ^{2}H quadrupolar splitting frequency ($\Delta\nu$) vs. the film orientation (θ). The data points are extracted from Supplementary Fig. 40f and the solid line represents a fit to Equation 1. The residual quadrupolar coupling constant χ for this BBL film is about 5400 Hz. **b,c**, Cyclic voltammetry of BBL in deuterated 100 mM (**b**) NH_4Cl and (**c**) NaCl electrolytes at a scan rate of 0.5 mV/s, run in the operando NMR cell.

To understand the relationship between intensity and splitting better, the operando ^{2}H NMR data for the NH_4Cl electrolyte is divided into three voltage ranges based on how the intensity changes with applied potential. These are colour-coded in Supplementary Fig. 42a,b as follows: (1) from 0 V to –0.3 V (green), where the intensity of the doublet peaks increases sharply; (2) from –0.3 V to –0.5 V (purple), where the intensity continues to rise but more gradually; and (3) from –0.5 V to –0.8 V (yellow), where the intensity begins to decrease due to water expulsion from the film. These intensity changes mirror the EQCM-D data. The correlation between the doublet splitting and intensity for each regime is shown in Supplementary Fig. 42c–h across three doping/dedoping cycles. Linear fits are applied to each regime, with the corresponding gradient labelled. The gradient of the linear fit in each regime reflects the change in χ with water injection into the anisotropic domains, as explored below; similar trends are observed between the three cycles, illustrating the reversibility of the water injection process.

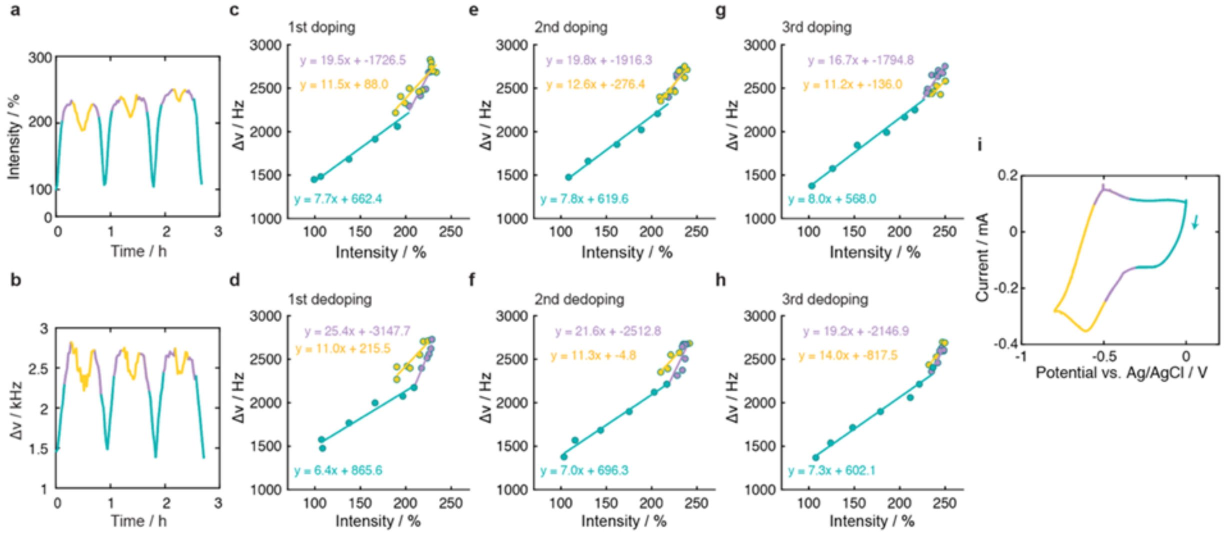

Supplementary Fig. 42 | a,b, Intensity of anisotropic water (**a**) and quadrupolar splitting (Δν) (**b**) from operando ^{2}H NMR measurements of BBL in 0.1 M NH_4Cl electrolyte, with colour-coded voltage ranges. **c–h**, Correlation between quadrupolar splitting and intensity across three doping and dedoping cycles, fitted

linearly within each regime. **i**, Colour-coded doping and dedoping regimes overlaid on the cyclic voltammogram, corresponding to the voltage ranges used in (**a–h**).

The quadrupolar splitting of water in anisotropic environments (Δν) depends on (i) the orientation of the BBL film substrate with respect to the external magnetic field B_0 (θ) and (ii) the quadrupolar coupling constant (χ), as described in Equation 1. The film orientation (θ) was constant during the operando ^{2}H NMR measurements. Consequently, the remaining variable in Equation 1 that can change with the doping level is the residual quadrupolar coupling constant (χ), which depends on (i) the nature of the interactions with water and the polymer domains restricting the water motion, (ii) the degree of ordering of the polymer domains that result in the constrained motion, and (iii) degree of ordering of these domains with respect to the film itself. The last two terms can be grouped together and quantified via an order parameter (S_{aniso}) which is likely to depend on water content in these regions and the level of doping. Thus, S_{aniso}, quantifies the degree of ordering and alignment of the domains, ranging from 0 for a completely disordered or isotropic domains to 1 for perfect alignment of ordered domains in the film with respect to each other and with respect to the substrate film. Exchange *i.e.*, dynamics of water molecules between the ordered (anisotropic) and disordered (isotropic) regions, and the quantity of water in each of the domains may also be important, resulting in an expression for the time-averaged (residual) value of the quadrupolar coupling constant associated with the doublet as described in Equation 2.

$$\chi = \frac{[D_2O]_{aniso}}{[D_2O]_{total}} S_{aniso}\chi_{aniso} + \frac{[D_2O]_{iso}}{[D_2O]_{total}} S_{iso}\chi_{iso} \approx \frac{[D_2O]_{aniso}}{[D_2O]_{total}} S_{aniso}\chi_{aniso} \qquad \text{Equation 2}$$

Here, $[D_2O]_{\text{aniso}}$ and $[D_2O]_{\text{iso}}$ represent the populations of anisotropic and isotropic water molecules, respectively. $[D_2O]_{\text{total}}$ is the total amount of water in the BBL films that is observed in these experiments (and is the sum of the water content in both the isotropic and anisotropic domains).

Water molecules are mobile and, at room temperature, they constantly undergo exchange between anisotropic and isotropic environments, which are associated with different time-averaged values of the quadrupolar coupling constants, χ_{aniso} and χ_{iso}, respectively. In isotropic domains, the quadrupolar coupling constant (χ_{iso}) and order parameter (S_{iso}) are by definition zero due to rapid isotropic motion, and no ordering of the polymer domains, which averages the quadrupolar interaction to zero. Therefore, the second term in Equation 2 is zero, and $\Delta\nu$ is proportional to the fraction of anisotropic water scaled by $S_{aniso}\chi_{aniso}$. According to Equation 2, the quadrupolar splitting ($\Delta\nu$) is expected to exhibit a linear relationship with the water intensity in the anisotropic domains, provided that the product of the anisotropic order parameter and quadrupolar coupling constant ($S_{aniso}\chi_{aniso}$) remains constant, *i.e.*, the BBL layered structure remains unchanged. This model assumes that the water is in rapid exchange with at least a subset of bulk (or isotropic) water. We explore the role of hydrated chloride ions in Supplementary Note 3.

Doping at low voltages (the green regime in Supplementary Fig. 42a), hydrated cations bring water molecules into the ordered BBL polymer matrix, consistent with the increase in intensity of the doublet resonances assigned to the water population in the anisotropic environment. This regime is also accompanied by a close-to-linear increase in peak splitting with intensity (Supplementary Fig. 42c), which has a gradient of 7.7 in the first doping cycle. In the second (purple) regime (from –0.3 V to –0.5 V, Supplementary Fig. 42c), the gradient is steeper; in this regime, the increase in intensity per unit of injected charge drops noticeably (Supplementary Fig. 43c). Together this indicates that fewer waters of hydration must accompany charge injection, indicating that the number of waters of hydration of the cations decrease

and/or that chloride ion expulsion commences. The EC-AFM measurements show that BBL film thickness increases from 0 to –0.3 V, followed by a decrease between –0.3 and –0.6 V, the thickness stabilising thereafter, consistent with the NMR observations.

In the first regime, 0 to –0.3 V, ions and water enter the layers, expanding the average layer spacing. Since an expansion of layers should actually result in a decrease in anisotropy as seen by the water, it is likely that in this regime layers that are originally empty or only partially hydrated are filled with more water (and associated cations). In the purple region (–0.3 to –0.5 V), the current increases rapidly suggesting the onset of a new process (Supplementary Fig. 42i). While the intensity and quadrupolar splitting change more slowly with time (Supplementary Fig. 42a,b), when the quadrupolar splitting is plotted against the intensity change (Supplementary Fig. 42c), the slope is larger for this regime. This is consistent with the onset of water expulsion and the layer collapse processes: we suggest that the expulsion of water leaves water within the constrained layers in between the polymer blocks, but now the water is even more constrained within the hydrated layer as the polymer film contracts, resulting in an increase in quadrupolar splitting. This phenomenon is much more pronounced above –0.5 V where the splitting now decreases (yellow regime in Supplementary Fig. 42b,c), yet ions are still being pumped into the film. At this point, the water intensity also decreases suggesting that the hydration number of the cations decreases. The number of water molecules in the anisotropic regions ($[D_2O]_{aniso}$) relative to the bulk decreases, reducing χ.

The quadrupolar splitting is also plotted as a function of injected charge and there is an inflection in the rate of change in the splitting at around -0.5 V (Supplementary Fig. 43a), indicating a transition in the mode of water incorporation due to the layer contraction. This interpretation is further supported by the water intensity vs. injected charge plot (Supplementary Fig. 43c), which provides a measure of the hydration state of the charge compensating cations inside the film. A decreasing water intensity with increasing injected charge (yellow regime in Supplementary Fig. 43c) suggests that water is leaving the film as more cations are being inserted.

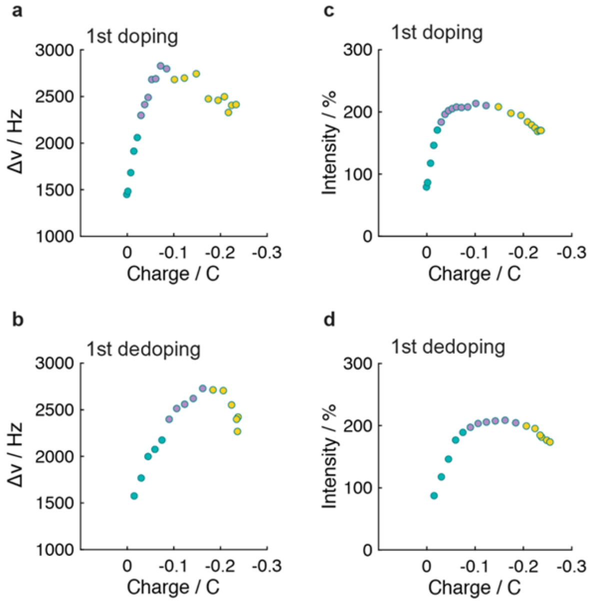

Supplementary Fig. 43 | a-d, Plots of ^{2}H NMR quadrupolar splitting ($\Delta\nu$) (**a,b**) and the water intensity in anisotropic domains (**c,d**) versus injected charge for the first doping and dedoping cycle in NH_4Cl, with

colour-coded voltage ranges same as previously defined in Supplementary Fig. 42 and the main text. Similar trends are seen in the subsequent cycles 2 and 3.

To summarise, in the first regime (green), the increase in χ is due to water accompanying cations into the space between the layers, resulting in swelling of the polymer, and increasing $[D_2O]_{aniso}$, since more water is contained in between the polymer layers. $S_{aniso}\chi_{aniso}$ likely also decreases as the polymer swells, reducing both terms, but the increase in $[D_2O]_{aniso}$ dominates. In the next regime (purple), the hydration number decreases and now the rate of change in both $[D_2O]_{aniso}$ and $S_{aniso}\chi_{aniso}$ are smaller. In the third region (yellow), $S_{aniso}\chi_{aniso}$ is expected to increase as the polymer shrinks, but $[D_2O]_{aniso}$ decreases more rapidly resulting in an overall decrease in quadrupolar splitting. Additionally, expulsion of hydrated chloride ions will also reduce $[D_2O]_{aniso}$ and thereby also contribute to the response in ^{2}H NMR. See Supplementary Note 3 for further detail.

Following the discussion of the NH_4Cl dataset, a similar analysis was carried out for the NaCl electrolyte to explore how the relationship between quadrupolar splitting and intensity differs during electrochemical cycling. The changes in anisotropic water intensity and quadrupolar splitting for NaCl are shown in Supplementary Fig. 44a,b, respectively. Due to the absence of a clear water expulsion phase, the voltage ranges were slightly redefined: (i) from 0 to –0.4 V (green), where the intensity increases sharply; (ii) from –0.4 to –1 V (purple), where the intensity increases more gradually and approaches a plateau; (iii) on dedoping from –1 to –0.4 V (yellow), where the intensity decreases slowly; and (iv) from –0.4 V back to 0 V (green), where the intensity drops sharply, similar to the initial doping phase, (i). The correlation between quadrupolar splitting and intensity is plotted in Supplementary Fig. 44c–h.

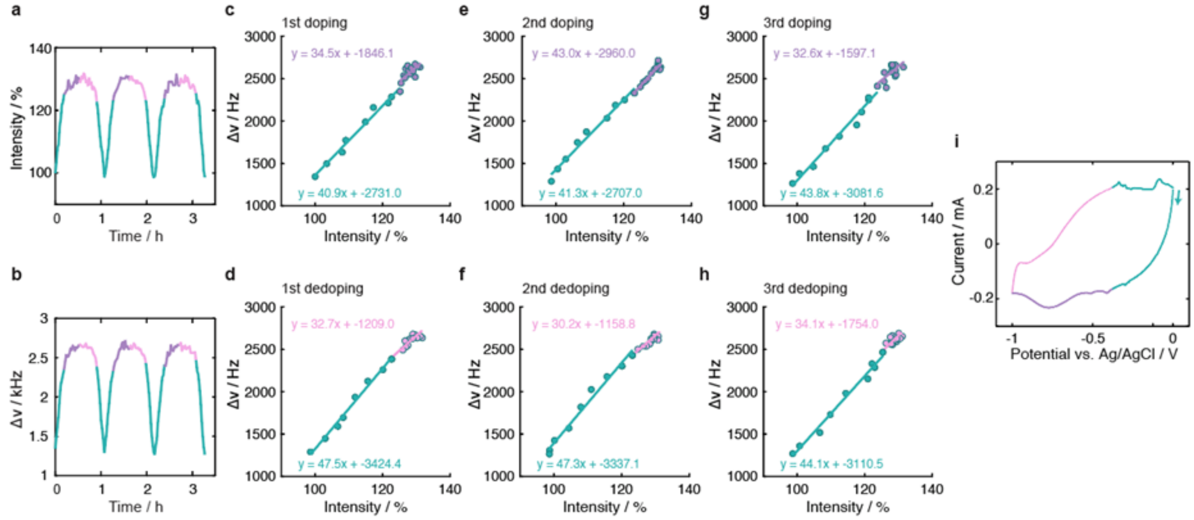

Supplementary Fig. 44 | a,b, Intensity of anisotropic water (**a**) and quadrupolar splitting (Δν) (**b**) from operando ^{2}H NMR measurements of BBL in 0.1 M NaCl electrolyte, with colour-coded voltage ranges. **c–h**, Correlation between quadrupolar splitting and intensity across three doping and dedoping cycles, fitted linearly within each regime. **i**, Colour-coded doping and dedoping regimes overlaid on the cyclic voltammogram, corresponding to the voltage ranges used in (**a–h**).

Across three electrochemical cycles, the average gradient in the green doping regime is 42.0 ± 1.6, which slightly decreases in the purple regime to 36.7 ± 5.5, suggesting a different mechanism. As the film doping

level increase, both the quadrupolar splitting and the water intensity increases with injected charge before gradually levelling off (Supplementary Fig. 45a,c).

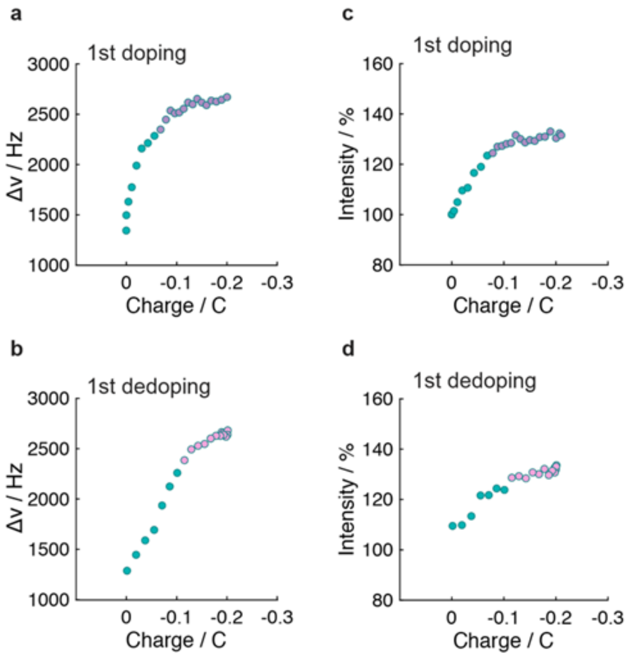

Supplementary Fig. 45 | a-d, Plots of ^{2}H NMR quadrupolar splitting ($\Delta\nu$) (**a,b**) and the water intensity in anisotropic domains (**c,d**) versus injected charge for the first cycle in NaCl, with colour-coded voltage ranges as defined in the text. Similar trends are seen in the subsequent cycles 2 and 3.

The initial rise indicates that at low to moderate doping levels (green region), the incorporation of hydrated ions drives additional water molecules into the BBL films, leading to an increase in $[D_2O]_{aniso}$ and thus $\Delta\nu$. However, as the doping level increases further (purple region), the film reaches a saturation point where additional charge compensation occurs with ions inserted with fewer accompanying water molecules. EC-AFM and EQCM-D data (Fig. 1c,d) support this interpretation. Upon dedoping, the trend reverses: the gradient increases from 32.3 ± 2.0 in the yellow regime to 46.3 ±1.9 in the final green regime, indicating a recovery of the initial structural state. The small changes in gradient compared to NH_4Cl indicate that there is a less pronounced change in the mechanism of charge compensation, with no clear feature associated with layer collapse and water expulsion.

In summary, BBL exhibits distinct structural responses when doped with NH_4Cl versus NaCl electrolytes, as evidenced by operando ^{2}H NMR, EQCM-D and EC-AFM measurements. BBL expands in both electrolytes at low doping, but at high doping it expels water (leading to a contraction) with NH_4Cl, but not NaCl electrolytes. These differences are attributed to the stronger interactions between NH_4^+ cations and the BBL backbone compared to Na^+. Importantly, operando ^{2}H NMR not only captures changes in the population of anisotropic water within the polymer matrix through signal intensity, but also reflects local structural transformations via variations in quadrupolar splitting.

Supplementary Note 3: ^{35}Cl NMR

During electrochemical doping of conjugated polymers, the movement of electrolyte ions is typically dominated by counter ion adsorption, however the possibility of ion expulsion of the opposite charge cannot be simply ignored. This competing process of ion expulsion has been shown to cause 'anomalous' mass changes in EQCM-D, as reported by Flagg and co-workers[24]. To investigate the role of chloride anions on the hydration of BBL films during electrochemical doping, operando ^{35}Cl NMR experiments were conducted in both 0.1 M NaCl and 0.1 M NH_4Cl electrolytes. Like ^{2}H, ^{35}Cl is a quadrupolar nucleus (I = 3/2), and its quadrupolar interactions can, in theory, be leveraged to probe the local environments of chloride ions, allowing us, in principle, to assess the chloride anion dynamics into and out of the BBL films during electrochemical doping. Supplementary Fig. 46a,b compares the ^{35}Cl NMR spectra of a 1 M NaCl solution in D_2O as a standard NMR reference to those of BBL films soaked in 0.1 M NH_4Cl and NaCl electrolytes in D_2O, respectively, prior to charging. In all cases, chloride ions appear as a singlet at 0 ppm. We then conducted operando ^{35}Cl NMR measurements during electrochemical doping (Supplementary Fig. 46c,d), with the corresponding voltage (Supplementary Fig. 46e,f) current (Supplementary Fig. 46g,h), and charge profiles (Supplementary Fig. 46i,j).

No anisotropic features (*i.e.*, peak splitting, satellite peaks or even peak broadening) were observed in the ^{35}Cl spectra during doping in either electrolyte. In contrast, the ^{2}H NMR measurements on the same system clearly showed residual quadrupolar splitting that tracked ion injection. Moreover, the linewidths of the ^{35}Cl signals in the soaked BBL samples are nearly identical to those in the NaCl solution, further indicating that ^{35}Cl nuclei experiences minimal interaction with its local environment. Throughout the electrochemical cycling, no changes were observed in the chemical shift of the singlet peak (Supplementary Fig. 46m,n), suggesting that the chloride ions remain in a similar chemical environment. However, the intensity of ^{35}Cl signal exhibited a clear dependence on the doping voltage in both electrolytes (Supplementary Fig. 46k,l). Specifically, a decrease in intensity was detected upon doping to more negative potentials, followed by a recovery to close to the original intensity during the reverse scan.

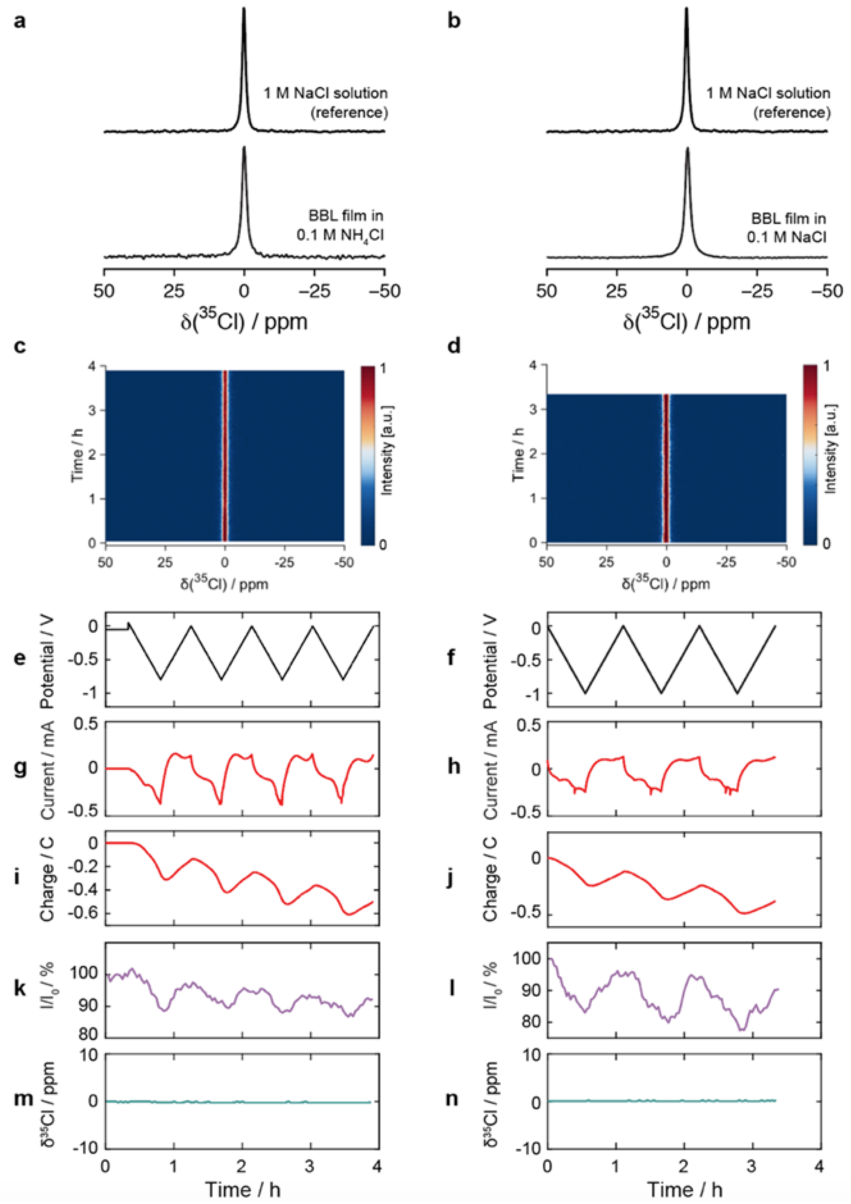

Supplementary Fig. 46 | ^{35}Cl NMR studies of BBL. **a,b**, ^{35}Cl NMR spectra of a 1 M NaCl solution in D_2O (as an NMR reference) compared with BBL films soaked in 0.1 M NH_4Cl and NaCl electrolytes in D_2O. Differences in signal-to-noise ratios among the spectra are due to varying numbers of scans: 8 scans for the 1 M NaCl solution, 128 scans for the BBL sample in 0.1 M NH_4Cl, and 117,760 scans for the BBL sample in 0.1 M NaCl. **c,d**, Operando ^{35}Cl NMR spectra recorded during electrochemical doping at a scan rate of 0.5 mV s^{-1} between 0 and -0.8 V or -1.0 V (vs Ag/AgCl), to monitor chloride ions movements in the bulk 0.1 M NH_4Cl and NaCl electrolytes and within the disordered isotropic region of BBL films, respectively. Corresponding **e,f**, voltage profiles; **g,h**, charging currents; **i,j**, injected charges; **k,l**, normalized ^{35}Cl signal intensities of the singlet peak; and **m,n**, chemical shifts of the singlet peak. Note that the drift in injected charge is likely caused by parasitic currents at high potentials.

^{35}Cl ions symmetrically coordinated by water will have a close to zero quadrupolar coupling. Furthermore, rapid isotropic motion will also average out any residual quadrupolar effects. If Cl^- ions were inserted into

a BBL film, we might expect that a distorted coordination (hydration) environment might induce a (transient) electric field gradient and thus quadrupolar interaction. Critically, bound chloride ions are generally invisible due to the fast quadrupolar induced relaxation[25]. We suggest that in this system the Cl^- ions are strongly hydrated (and thus in symmetric environment) and mobile, and are therefore relatively insensitive to insertion and removal from the film. ^2H nuclei by contrast in D_2O are associated with large χ values and the D_2O molecules appear to be more strongly affected (constrained) by the BBL films, potentially by hydrogen bonding. Further experiments using ND_4^+ (and Na^+) cations might provide insight into the hydrogen bonding interactions in these films. Additionally, we note that no ^{35}Cl peak splitting was seen in the PEDOT:PSS system[10], again suggesting that well defined interactions with the polymer matrix are required so as to observe a quadrupolar splitting, even if the underlying matrix is anisotropic.

The observed decrease in ^{35}Cl intensity indicates a loss of chloride ions (in an isotropic environment) on the BBL side of the cell, which can be assigned to chloride ions originating from the bulk electrolyte near the film or in hydrated environments within the BBL film. Specifically, any chloride ions expelled from the BBL film into the bulk electrolyte would subsequently migrate toward the counter electrode (PEDOT:PSS), which was outside the NMR detection region, and thereby contribute to a decreased signal. Such chloride ion expulsion is in agreement with previous work on counterion expulsion during electrochemical doping[24]. Furthermore, if chloride expulsion from the BBL film underlies the observed decrease in ^{35}Cl intensity, these ions must originate from isotropic environments in the film since expulsion from tightly bound (and thus not visible in ^{35}Cl NMR) environments would not change the intensity or even increase it as the expelled chloride ions (from these anisotropic environments) partially remain in the bulk electrolyte. Additionally, from ^2H NMR we know that the water (D_2O) intensity inside the film increases significantly on injecting negative charge which is not consistent with hydrated Cl^- ion expulsion.

For NH_4Cl, we observe a small and continuous decrease in ^{35}Cl intensity which becomes more pronounced at more extensive doping. Thereby the ^{35}Cl intensity is consistent with chloride expulsion and BBL polymer contraction, especially above -0.5V, and it is likely that some charge compensation does occur via this mechanism. It should be noted that there is an additional contribution from a baseline drift which partially underlies this intensity decrease (*vide infra*). For NaCl the steady (and more pronounced) drop in intensity over the entire doping range, even when the water content in the films is increasing, is in contrast to the mass/thickness response for NaCl.

Notably, there are two additional sources of decrease in ^{35}Cl signal: (i) the drift of Cl^- towards the positive electrode, (ii) the effect of the expansion of the BBL film on the free electrolyte detected in the NMR experiment. The sample inside the coil in our NMR design comprises a separator (soaked in electrolyte) approximately 1 mm thick, a thick BBL film and two current collectors, with external pressure applied via the capsule cell. The expansion of the BBL film will exert pressure on the separator and will likely expel some of the liquid in its pores, reducing the overall ^{35}Cl ion signal. Because we cannot separate bulk electrolyte from BBL-sorbed chloride ions, we must take this phenomenon into account and we suggest that the changes in intensity seen for the ^{35}Cl are also affected by this. Note that the ^2H water signals are not affected by this as we can quantify ions in the anisotropic regions in the film.

Another relevant observation is that the ^{35}Cl intensity gradually drops with cycle number. We find this decrease in intensity to track the additional (excess) negative charge injected into the film (Supplementary Fig. 46i,j), which we have ascribed to parasitic reactions consuming electrons. Thus, we suggest that a reaction such as

$$2\,H_2O + 2\,e^- \rightarrow \,H_2 + 2\,OH^- \qquad \text{Equation 3}$$

which liberates hydroxide ions could end up expelling Cl^- ions in or near the film. It is also possible that the separator becomes progressively more compressed with cycle number, resulting in a reduction of electrolyte in it.

In conclusion, we observe that the Cl^- is continuously expelled while doping with both electrolytes, with a significantly higher intensity loss for NaCl than for NH_4Cl. This is in stark contrast to the ^2H NMR signal and mass response which showed a decrease exclusively for NH_4^+ occurring only at the highest applied bias (Fig. 1 and Fig. 2). The more pronounced loss of ^{35}Cl intensity for NaCl, however, matches the preswollen Δmass (see Supplementary Figs. 27 and 28). Additionally, we note that the expelled chloride ions originate from isotropic environments and therefore do not contribute to the ^2H NMR signal. Therefore, we conclude that Cl^- anions are being expelled during doping, but this anion expulsion does not underly the observed deswelling and ^2H NMR signal which is ascribed to H_2O expulsion through these complementary operando ^2H and ^{35}Cl NMR measurements.

Supplementary Note 4: Additional observations on morphological changes upon doping and dedoping from EQCM-D and ^{2}H NMR

As discussed in the main text, we conclude from EQCM-D and ^{2}H NMR experiments that, at a sufficiently high bias, water is expelled from the BBL film (for protic cations, NH_4^+) or the amount of water in the film remains approximately constant (for aprotic cations, Na^+) upon injecting additional cations during doping. Both cases amount to a lowering of the amount of water molecules per cation in the BBL film caused by the high doping, as also confirmed by MD simulations (Fig. 4 and Supplementary Note 7).

Upon closer inspection of the EQCM-D, we find that the mass during the intermittent 0 V step is not a constant throughout a measurement. Specifically, we find that a high applied bias facilitates a rapid decrease in mass towards the 0 V level, but that switching from an intermediate applied bias to 0 V gives an initial fast decrease in mass followed by a slow continuous decrease towards the 0 V mass level (*e.g.*, Supplementary Fig. 47). Strikingly, this effect is found for all of the electrolytes employed here (Supplementary Figs. 2, 3, 23-26) and occurs at an applied bias beyond their inflection point in mass, or at the first bias exhibiting a mass decrease, hinting at a common mechanism among electrolytes.

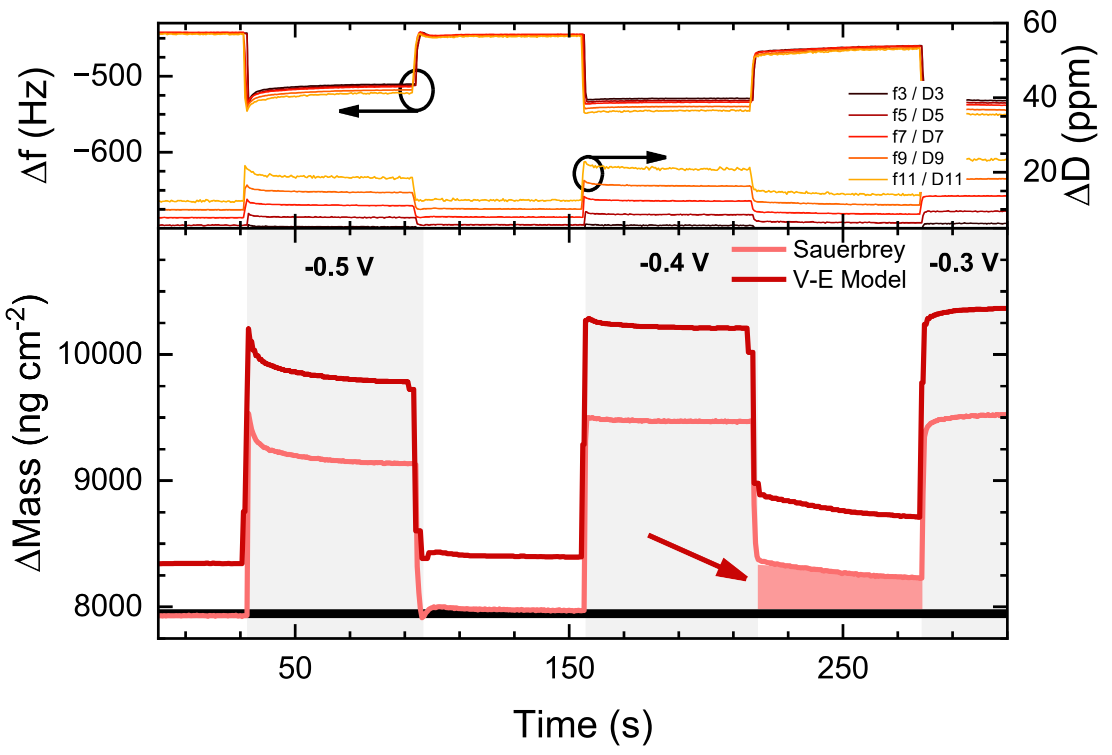

Supplementary Fig. 47 | The measured Δf (left axis) and ΔD (right axis) for the 3rd-11th overtone from EQCM-D measurements on BBL submerged in 0.1 M NH_4Cl. The applied bias is decreased stepwise from -0.5 V to -0.3 V vs Ag/AgCl with intermittent 0 V steps. Bottom panel includes the Δmass from the Sauerbrey equation and visco-elastic (V-E) modeling relative to the dry mass of the BBL film. The area below the second intermittent 0 V step is highlighted, which clearly shows a slow decrease in mass that follows the initial rapid decrease in mass upon switching from the intermittent (-0.4 V) bias to 0 V bias. This effect is not observed for switching from -0.5 V to 0 V in the preceding bias steps.

Based on our previous EQCM-D and ^{2}H NMR results, we can form a hypothesis on the morphological changes underlying this effect. We established that fewer water molecules per injected cation are present at high doping levels compared to intermediate doping levels, regardless the employed cation. Therefore, at high applied bias, the BBL film is in a relatively water-poor state with many cations that are rapidly removed upon switching to 0 V, bringing along a large fraction of the few water molecules in the film. At intermediate applied bias, the BBL film is instead in a water-rich state where fewer cations are surrounded by more water molecules. Upon switching from this state, the cations are again removed rapidly but they bring a comparatively lower fraction of the water molecules with them. The excess water compared to the 0 V state that remains does not have a strong driving force to leave the film, leading to a slow secondary decrease in mass. This mechanism is depicted in Supplementary Fig. 48 and would give rise to a rapid decrease in mass for highly doped states and a 2-step decrease in mass for intermediately doped states where the first step (moving ions and water) is quick, and the second step (excess water expulsion) is slow. Exactly as according to our observations from EQCM-D.

The observed slow mass relaxation upon switching from intermediate doping biases can have a pronounced effect on device and/or material function, as these mass changes will lag behind the electronic and ionic dedoping response.

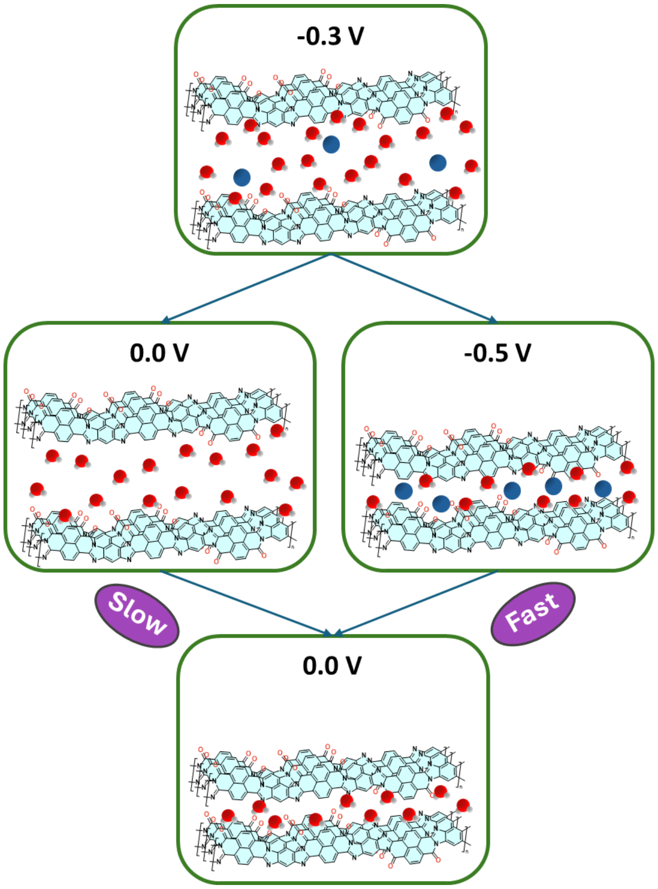

Supplementary Fig. 48 | Schematic depiction of the hypothesized morphological changes during doping underlying the observed fast and slow decrease in mass upon dedoping from high and intermediate biases, respectively.

Supplementary Note 5: DFT study of IR absorption spectra as a function of charge state and hydrogen bonding

We have examined the robustness of IR spectra predictions by comparing the results of three different theoretical models: 1) b3lyp/6-311g(d,p) functional/basis set with grimme D3 dispersion corrections and PCM solvation model for water; 2) b3lyp/def2tzvp functional/basis set with grimme D3 dispersion corrections and PCM solvation model for water; 3) wb97xd/6-31g* functional/basis set with PCM solvation model for water. In each case, we optimized the geometry of the BBL monomer, aka perinone, in a syn configuration, in two charge states: the neutral molecule interacting with one ammonium cation, BBL(0) : 1 NH_4^+ (+1), with a total charge = +1; and a singly negatively charged molecule interacting with one ammonium cation, BBL(-1) : 1 NH_4^+ (+1), with a total charge = 0. All calculations were performed using the Gaussian 16 package[26]. As commonly done in the literature[27], we rescaled the vibrational frequencies by *ad hoc* specific factors for each level of theory: 1) 0.98; 2) 1.00 (no scaling); 3) 0.945. The predicted spectra are shown in Supplementary Fig. 49 and important results tabulated in Supplementary Table 3. We conclude that the overall shape of the IR spectra is obtained at the three levels of theory, with only minor spectral shifts and changes in intensity going from one level to another.

Supplementary Table 3: IR frequencies of the C=O bond obtained with different theoretical set up. A scaling factor was used for the comparison of the IR spectra. All frequencies values are in cm^{-1}.

Method	b3lyp/6-311g(d,p)		b3lyp/def2tzvp		wb97xd/6-31g*	
Scaling factor	0.98		1.00		0.945	
System	BBL(0) qtot = +1	BBL(-1) qtot = 0	BBL(0) qtot = +1	BBL(-1) qtot = 0	BBL(0) qtot = +1	BBL(-1) qtot = 0
.ν (C=O) free	1707	1642	1716	1650	1719	1648
ν (C=O) ion	1673	1564	1673	1570	1693	1558
Δν	34	78	43	80	26	90
I(ratio) V(free)/v(ion)	0.73	0.80	0.78	0.98	0.65	0.61

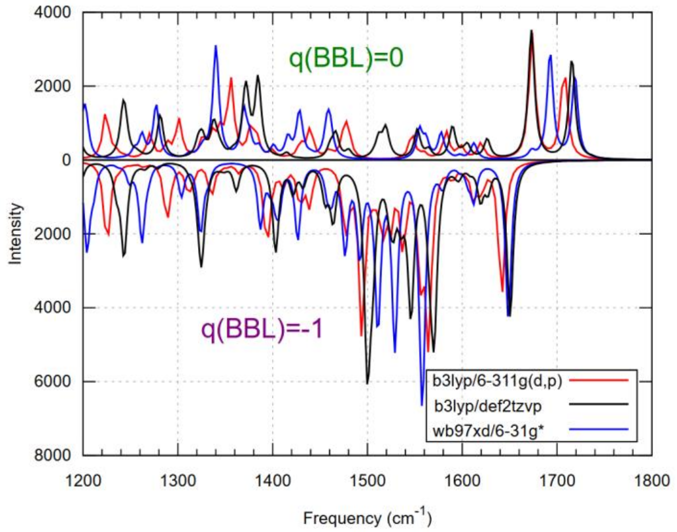

Supplementary Fig. 49: IR spectra computed for the neutral and singly reduced models of BBL at three different levels of theory, see text for details.

In view of the good agreement between results afforded by different models, all further calculations were performed at the b3lyp/def2tzvp/D3/PCM(water) level of theory. We considered multiple charge states for the BBL perinone monomer (thus half BBL repeating unit): pristine (no counter-ions) and a singly/doubly negatively charge states. The IR-spectra are shown in subsections a-f below and Supplementary Table 4 compiles the spectral position of the IR peaks associated to C=O vibrations. Calculations were performed in absence (subsection a) and in the presence of interacting ammonium ions (NH_4^+) localized close to the C=O (b) or the N site (c), in absence and in presence (d, e) of excess water molecules (as we have observed that this can influence protonation). A summarizing picture is provided in section (f).

DFT modeling of the IR spectra of the perinone monomer in its undoped and doped states matches relatively well the experimental observations for BBL, especially when considering (partial) protonation at high doping. Most importantly, the C=O vibration in the pristine molecule (calculated at ~1710 cm^{-1}) is shifted to lower wavenumbers (~1650 cm^{-1}) when the molecule is reduced to its singly negatively charged state in absence of counter-ions. This is accompanied by an increase in the intensity of the aromatic core breathing around 1500 cm^{-1}. A further red shift of the C=O stretching to ~1580 cm^{-1} (now overlapping/mixing with the aromatic core breathing) occurs when adding a second negative charge on the bare molecule. Experimentally, we see the disappearance of the C=O stretching at 1720 cm^{-1} (from the neutral state) and the emergence of two strong IR absorptions at 1630 and 1540 cm^{-1} upon doping. We tentatively assign the 1630 cm^{-1} mode to the shifted C=O vibration and the 1540 cm^{-1} mode to the polaronic, enhanced, aromatic core breathing mode. While this matches well the theoretical results at low doping, there is no clear experimental evidence of an increased spectral shift of the C=O stretching at higher level of doping, as would be expected from the ion-free simulations.

The simulated IR spectra reported in Supplementary Fig. 51, 53 (subsection b,d) show various degrees of spectral shifts and reshuffling of the IR absorption intensity when comparing the results for free (in absence of counterions) versus bound (in presence of counterions) C=O vibrations, indicating strong hydrogen bonding between the C=O groups and the ammonium cations. Interestingly, we find that ammonia localized close to the imidazolium nitrogen affords an N-protonated form of perinone (r(N:H) ~1 Å, Supplementary Fig. 52 subsection c). This N-protonated form of the doubly charged molecule displays vibration at about the same spectral range (around 1650 cm^{-1}) than the C=O vibration of the (ion-free) singly charged molecule, in line with experimental results. This result, however, was obtained in the absence of H_2O, and we find protonation (and the ~1650 cm^{-1} peak) is not reproduced in the presence of H_2O (Supplementary Fig. 54, 55 and Supplementary Table 4). Additionally, we note that an N-H vibration is not found in the experimental IR spectrum for NH_4Cl or NaCl (Supplementary Fig. 12, 13).

Of course, these calculations need to be taken with a grain of salt in view of the approximations applied in our modelling protocol. Therefore, the 'reality' (which is a complex case to describe based on single reference determinants approaches as that applied here) might lie somewhere between the extreme cases of weak H-bonds and full protonation and likely evolves continuously as the amount of electron transfer from the reduced BBL to the proton steadily increases (depending namely on the number of interacting water molecules and the associated local dielectric response). Therefore, we conclude these IR measurements and calculations hint at a nuanced balance between (partial) protonation and hydrogen bonding that is not fully elucidated. An additional, more clearcut fingerprint for the specific interactions between ions and BBL, is provided by the theoretical analysis of the cyclic voltammograms in Supplementary Note 6.

Supplementary Table 4 | DFT calculated C=O vibration frequency for all configurations considered, either free (no ions) or in presence of the ions. Ions can be localized either close to the carbonyl (/ C=O) or the imidazolium nitrogen (/N). Values followed by * indicate that C=O or N was protonated (partially or totally) during the relaxation. All frequencies are in cm^{-1}.

			q BBL = 0		q BBL = -1		q BBL = -2	
		Position of ion	C=O free	C=O ion	C=O free	C=O ion	C=O free	C=O ion
BBL (free)	Anti	-	1707	-	1637	-	1583	-
	Syn	-	1710<br>1726	-	1635<br>1650	-	1583<br>1596	-
BBL + 1 NH_4^+	Anti	/ C=O	1713	1672	1654	1569	1599	1549
		/ N	1712	-	1640	-	1589*<br>1598*	-
	Syn	/ C=O	1716	1673	1650	1570	1597	1532
		/ N	1720<br>1723	-	1640<br>1656	-	1593*<br>1596*	-
BBL + 1 (NH_4^+ + 4 H_2O)	Anti	/ C=O	1713	1648	1654	1566	1598	1565
		/ N	1712	-	1640	-	1589	-
	Syn	/ C=O	1716	1658	1651	1587	1598	1564
		/ N	1713<br>1721	-	1649	-	1590	-
BBL + 2 NH_4^+	Anti	/ C=O	-	1672	-	1498	-	1452
		/ N	1719	-	1645	-	1636*	-
	Syn	/ C=O	-	1675<br>1682	-	1514	-	1422<br>1435
		/ N	1722<br>1728	-	1647<br>1663	-	1638*	-
BBL + 2 (NH_4^+ + 4 H_2O)	Anti	/ C=O	-	1681	-	1570	-	1568
		/ N	1719	-	1643	-	1596	-
	Syn	/ C=O	-	1688	-	1561<br>1589	-	1570<br>1573
		/ N	1719<br>1726	-	1658	-	1578	-

a) IR spectra as a function of charge state (free perinone)

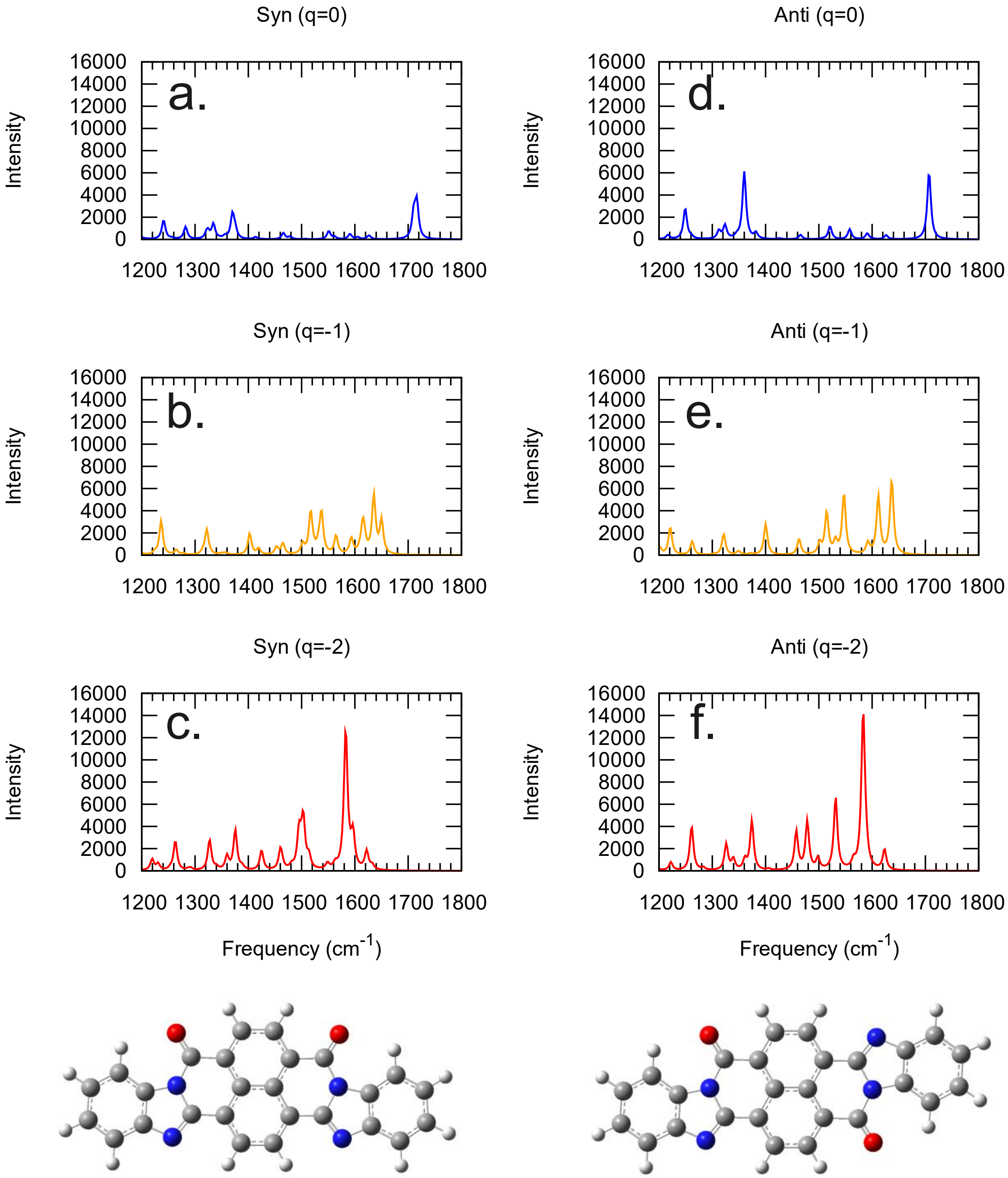

Supplementary Fig. 50 | a-f, DFT computed infra-red spectra of syn (**a-c**) and anti (**d-f**) configurations of one half BBL unit, a.k.a. perinone. The color code refers to the charge of the BBL unit: neutral (blue), singly negatively charged (orange) and doubly negatively charged (red).

b) IR spectra for BBL + NH_4^+ facing C=O

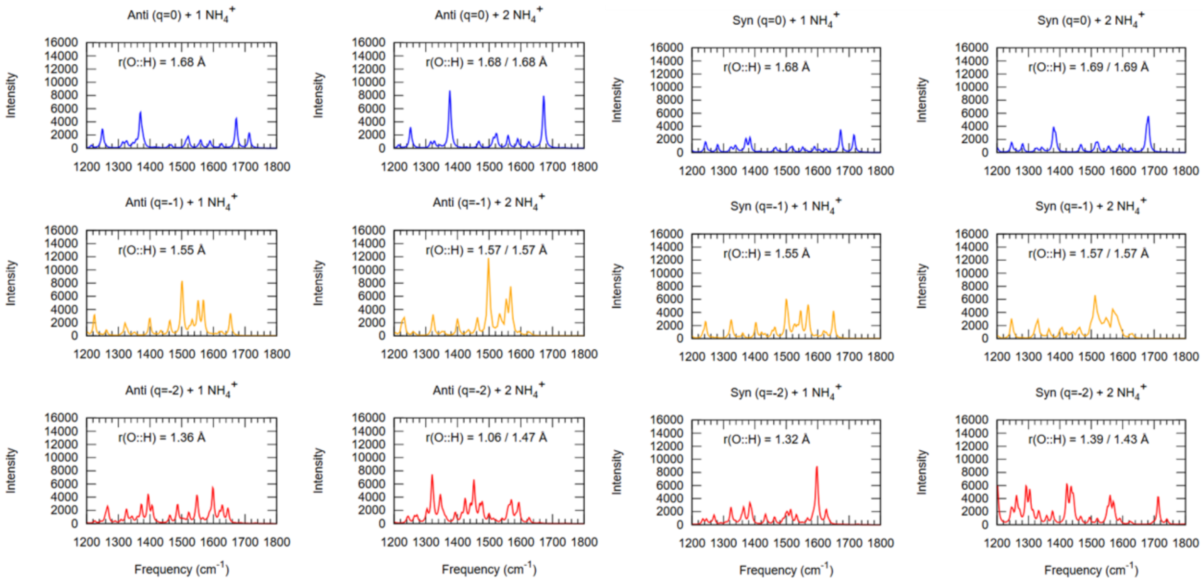

Supplementary Fig. 51 | DFT computed infra-red spectra for the Anti or Syn perinone with 1 or 2 ammonium ions localized close to carbonyl functions. The color code refers to the charge state: neutral (blue), singly negatively charged (orange) and doubly negatively charged (red). The shortest distance between the oxygen of the carbonyl and the hydrogen from the ammonium, r(O:H), is given for information for each case.

c) IR spectra for BBL + NH_4^+ facing N

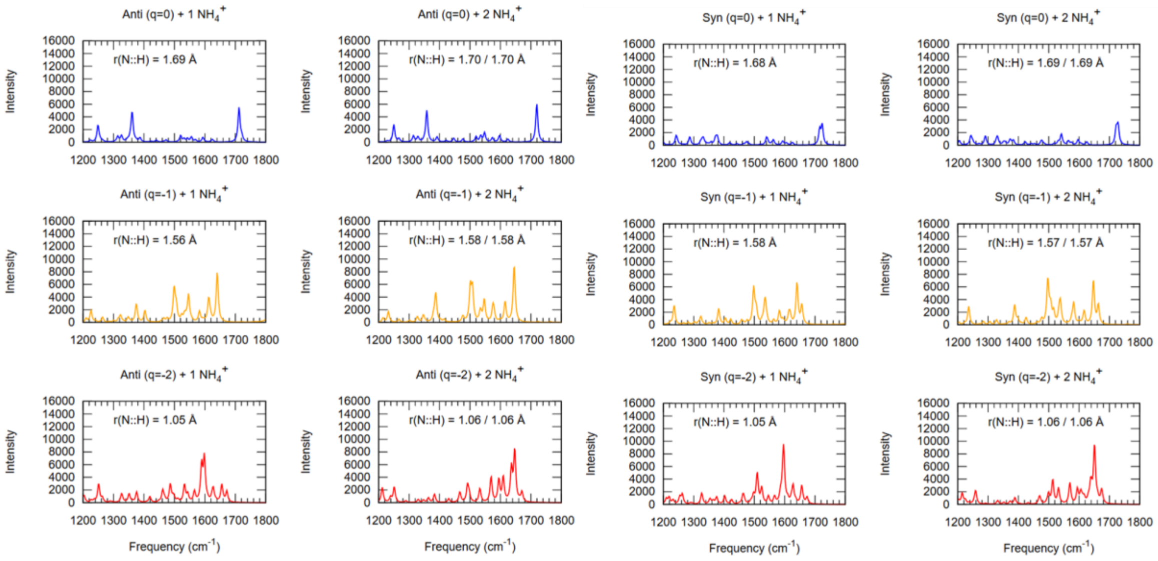

Supplementary Fig. 52 | DFT computed infra-red spectra for Anti or Syn perinone with 1 or 2 ammonium ions localized close to imidazolium nitrogen. The color code refers to the charge state: neutral (blue), singly negatively charged (orange) and doubly negatively charged (red). The shortest distance between the nitrogen of the imidazolium moiety and the hydrogen from the ammonium, r(N:H), is given for information for each case.

d) IR spectra for BBL + (NH_4^+ + 4 H_2O) facing C=O

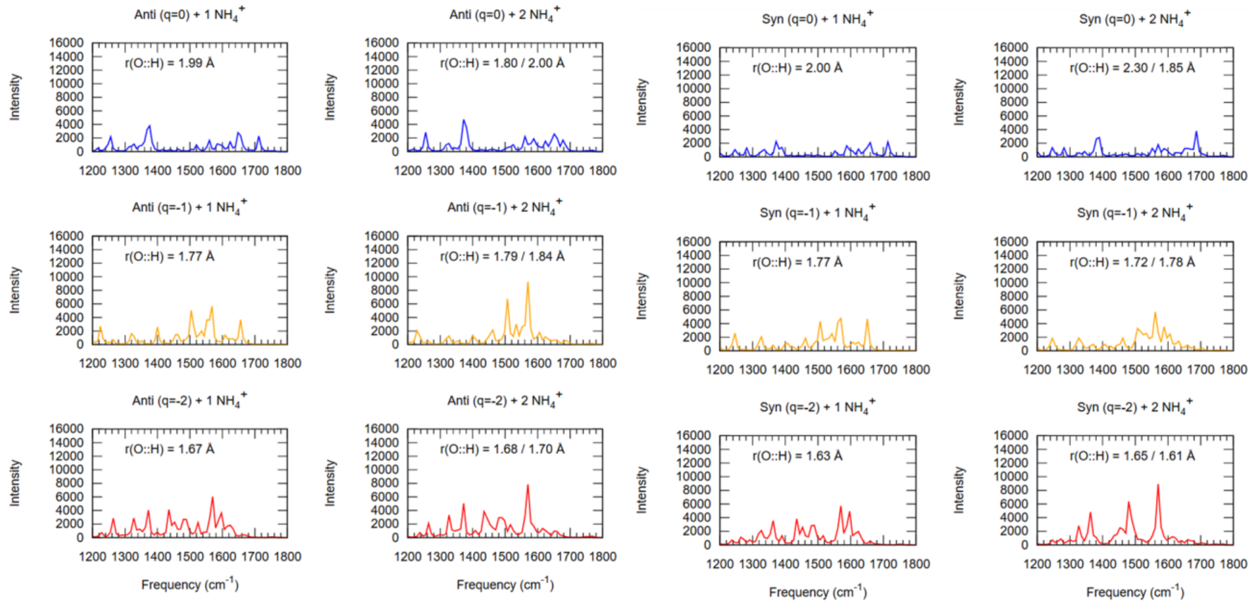

Supplementary Fig. 53 | DFT computed infra-red spectra for Anti or Syn perinone with 1 or 2 ammonium ions localized close to carbonyl functions and surrounded by 4 water molecules. The color code refers to the charge state: neutral (blue), singly negatively charged (orange) and doubly negatively charged (red). The shortest distance between the oxygen of the carbonyl and the hydrogen from the ammonium, r(O:H), is given for information for each case.

e) IR spectra for BBL + (NH_4^+ + 4 H_2O) facing N

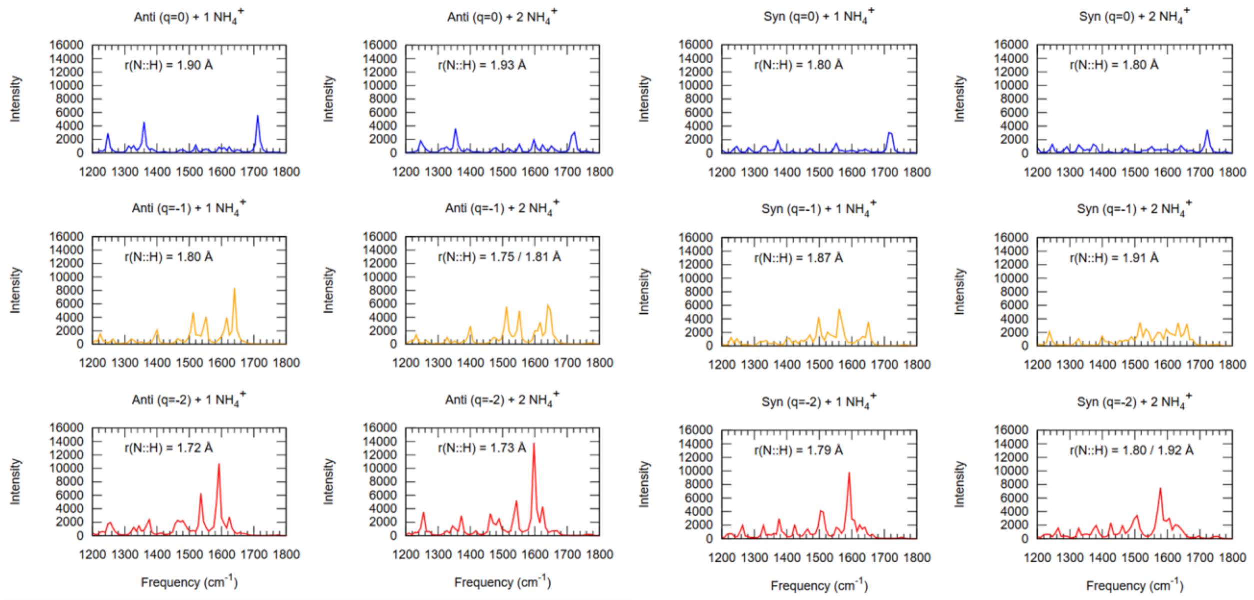

Supplementary Fig. 54 | DFT computed infra-red spectra for the Anti or Syn perinone with 1 or 2 ammonium ions localized close to imidazolium nitrogen and surrounded by 4 water molecules. The color code refers to the charge state: neutral (blue), singly negatively charged (orange) and doubly negatively

charged (red). The shortest distance between the nitrogen of the imidazolium moiety and the hydrogen from the ammonium, r(N:H), is given for information for each case.

f) Selection of IR spectra focused on BBL -2 / $2NH_4^+$ cases

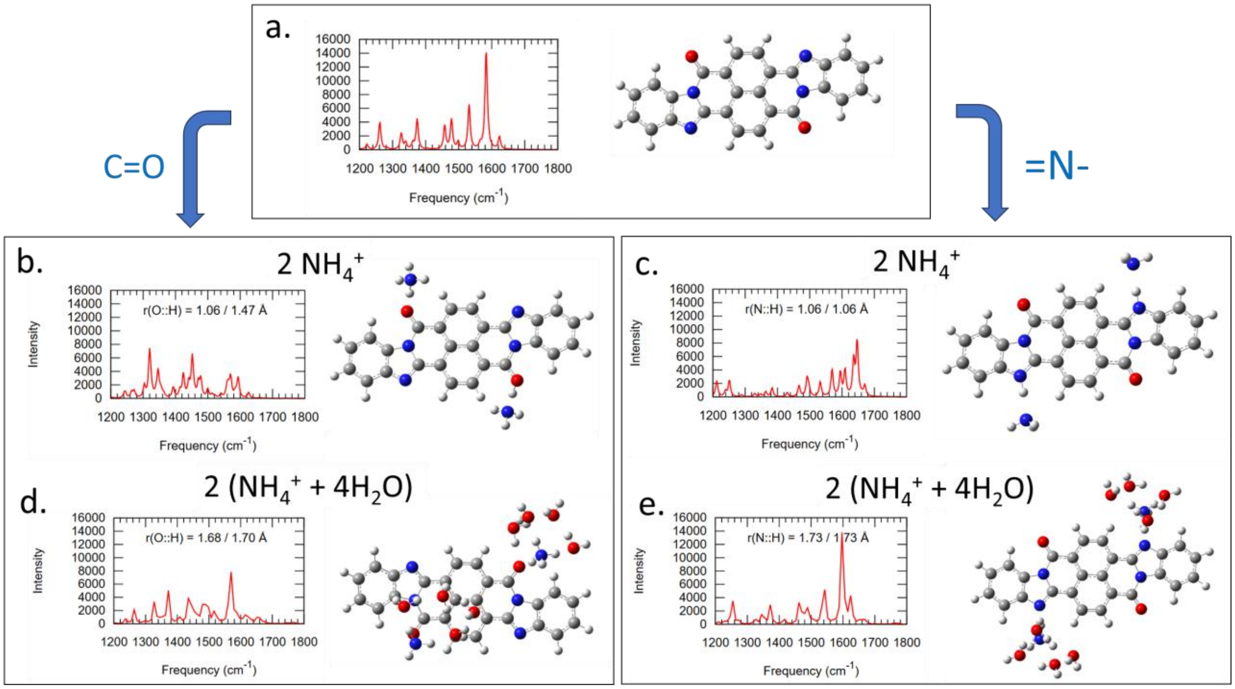

Supplementary Fig. 55 | DFT computed IR-spectra for anti-configuration of perinone in contact with two ammonium ions in its doubly charge state. **a**, IR-spectrum without ammonium ions is given for reference. **b-c**, IR-spectra for ammonium ions in close contact with C=O (**b**) or imine nitrogen (**c**) with corresponding optimized structures. Note that protonation of the BBL occurs during the relaxation at this level of calculation. **d-e**, IR-spectra when ammonium ions are surrounded by 4 water molecules. Crucially, no protonation is observed on both the final structures.

Supplementary Note 6: Computational modelling of cyclic voltammetry data – influence of pH and H-bonding

There are conflicting views on the importance of protonation during the doping process of perinone-based materials in aqueous electrolytes[28]. While IR and CV data of perinone-based COFs in highly acidic solutions suggest protonation occurs[29], the CV voltammograms reported here for BBL are markedly different and strongly affected by the nature of the cations (namely their ability to form H bonds) while the pH remains in the range 5-7 (Supplementary Fig. 29). As a first attempt to seek for possible protonation effects, we have performed geometry optimizations at the DFT level (b3lyp/def2tzvp/D3/PCM(water)) of the anti and syn forms of perinone at the charge state 0, -1 and -2, in the presence of a small water cluster brought in contact with the C=O or the imidazolium nitrogen functions, see Supplementary Fig. 56 and 57. The cluster was made of 1 H_3O^+ and up to 4 H_2O molecules (added one by one). We find that in the neutral and singly reduced forms, the H_3O^+ ions form a stable H-bonding network with the surrounding water molecules and the perinone C=O, but there is *no* protonation. In contrast, a proton transfer seems to occur when going to the doubly reduced form of the perinone. We stress, however, that this is sensitive to details of the models, number of molecules and arrangement of the water solvation shell, initial structure, etc. From these preliminary investigations, we conclude that the neutral and singly reduced forms of perinone are not basic enough to capture a proton in an acidic environment, while this might occur for the more basic doubly reduced monomer.

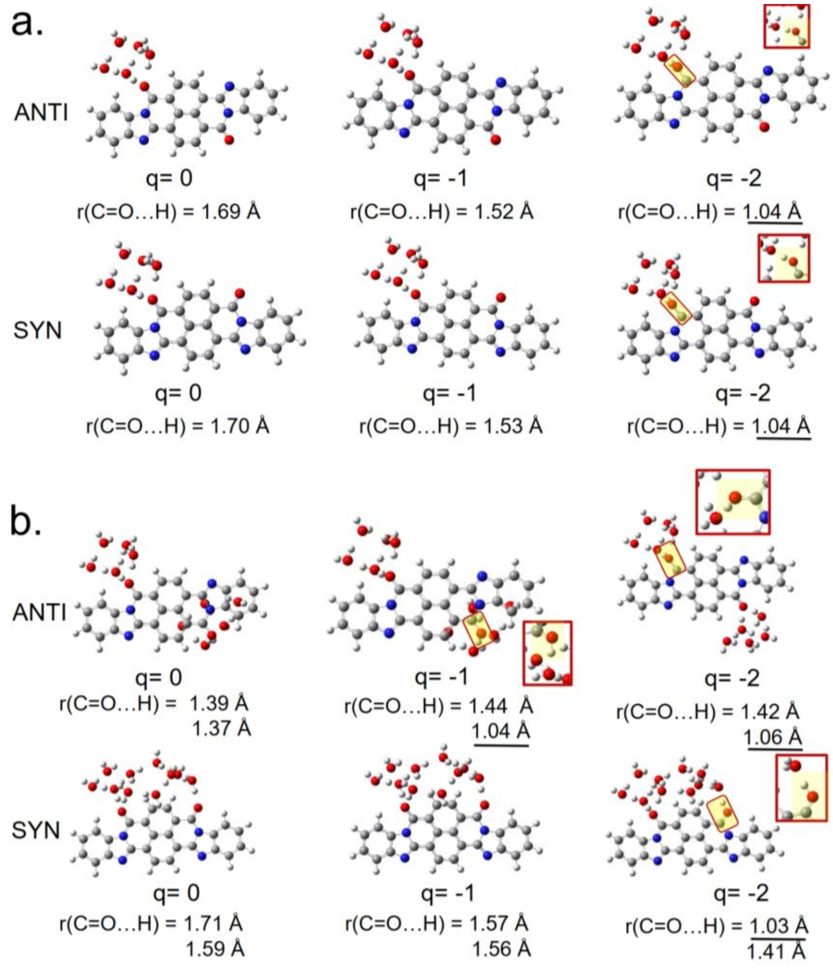

Supplementary Fig. 56 | a,b, Optimized perinone molecule with one (**a**) or two (**b**) clusters made of 1 H_3O_+ and 4 H_2O in function of the charge state of the perinone. The distance between C=O function and the closest hydrogen is given for information. Short C=O…H bonds associated with a proton transfer and the formation of an -OH group are highlighted. Alternative views of the -OH groups are given in the red squares.

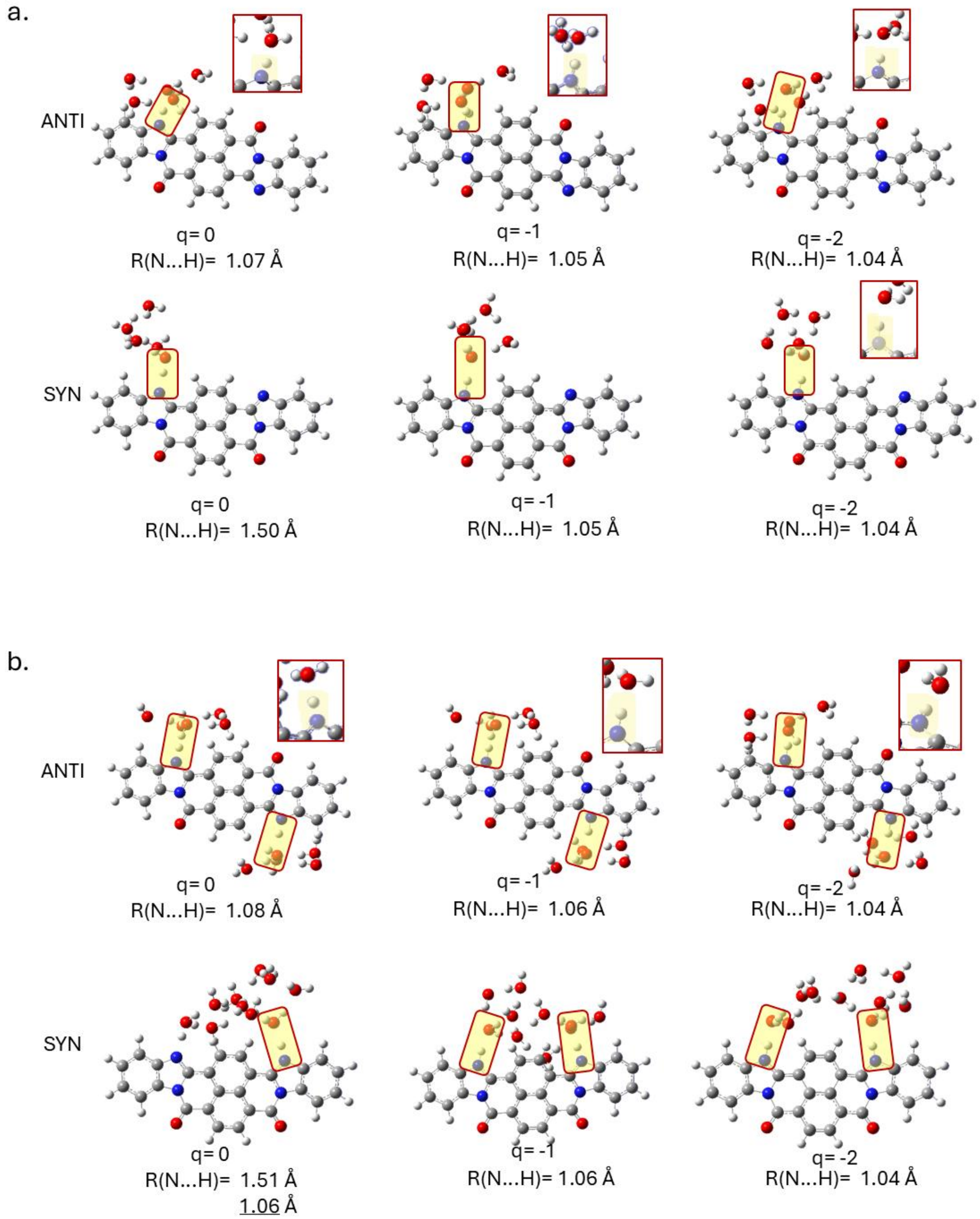

Supplementary Fig. 57 | a,b, Optimized perinone molecule with one (**a**) or two (**b**) clusters made of 1 H_3O_+ and 4 H_2O in function of the charge state of the perinone. The distance between nitrogen and the

closest hydrogen is given for information. Short N…H bonds associated with a proton transfer and the formation of an -NH group are highlighted. Alternative views of the -NH groups are given in the red squares.

A deeper investigation of the possible impact of pH was made through simulation of cyclic voltammetry (CV) considering the following scheme of square (Supplementary Fig. 58) where E and C stands for electron transfer and chemical reactions (here protonation), respectively. The redox potentials and pK_a data were computed by DFT using the gaussian 16 software at the ub3lyp/def2tzvp level with grimme-D3 correction and a PCM solvation model to include water.

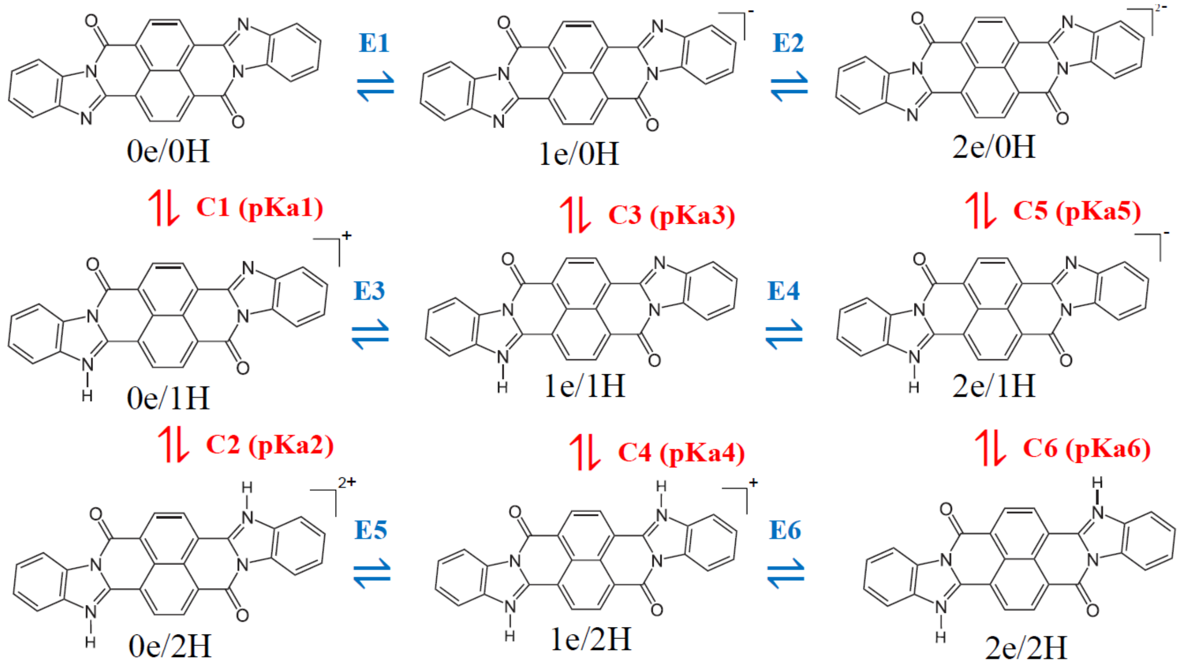

Supplementary Fig. 58 | Considered scheme of square for the anti form perinone with protonation on nitrogen. The same approach was used for the syn-conformation. The effect of protonation on carbonyl functions was also considered for comparison. ne/mH notation stands for the number of additional electron and/or proton respect to the initial neutral unprotonated form.

The redox potential E is defined from the Gibbs free energy difference of an electron transfer (ET) reaction as:

$$\Delta G(ET) = -n\,F\,E$$

$$\Delta G(ET) = G(A^-) - G(A) + n\,F\,E_{eff}(e,SHE)$$

where G is the total Gibbs free energy of the compound before (A) and after (A^-) an electron transfer. $E_{eff}(e,SHE)$ is the effective absolute potential and was set to 4.44 V[30].

The pK_a of a proton dissociation reaction was defined using the direct approach as:

$$A\text{-}H \rightleftharpoons A^- + H^+$$

$$pKa = \frac{\Delta G_{diss}}{RT\ln 10}$$

$$\Delta G_{diss} = G(A^{-}) - G(AH) + G(H^{+})$$

where $G(H^{+})$, the Gibbs free energy of the solvation of the proton, was set to -270.3 kcal/mol[31].

Supplementary Table 5 | Computed redox potential (in V) and pK_a for the anti- and syn-forms of perinone assuming a protonation on nitrogen or on carbonyl function. The labelling of E and pK_a refers to the scheme in Supplementary Fig 58.

	**Protonation on N**		**Protonation on C=O**	
	**Anti**	**Syn**	**Anti**	**Syn**
E1	-0.481	-0.454	-0.481	-0.454
E2	-1.131	-1.115	-1.131	-1.115
E3	+0.076	+0.066	+0.283	+0.319
E4	-0.625	-0.639	-0.481	-0.416
E5	+0.563	+0.587	+0.980	+1.022
E6	-0.111	-0.081	+0.130	+0.323
pK_a1	-1.9	-1.0	-16.7	-17.0
pK_a2	-5.5	-5.9	-21.5	-22.4
pK_a3	+7.6	+7.8	-3.8	-4.0
pK_a4	+2.8	+2.6	-9.7	-10.5
pK_a5	+16.1	+15.8	+7.2	+7.8
pK_a6	+11.5	+12.0	+0.6	+2.0

On the basis of the computed redox potentials and pK_a (Supplementary Table 5), we have generated the Pourbaix diagrams in the two considered protonation scenarios (C=O or imine N). Those show that partial protonation of the imidazolium nitrogen can be expected around neutral pH (Supplementary Fig. 59). Note, however, that this is based on a thermodynamical point of view only. In fact, including the same data input in the simulation of CV voltammograms with kinetics and concentration parameters leads to the apparition of protonation around pH 4 and below, while the unprotonated forms remain the majority species between pH 5-7 (see the discussion below and concentration profiles, Supplementary Fig. 61).

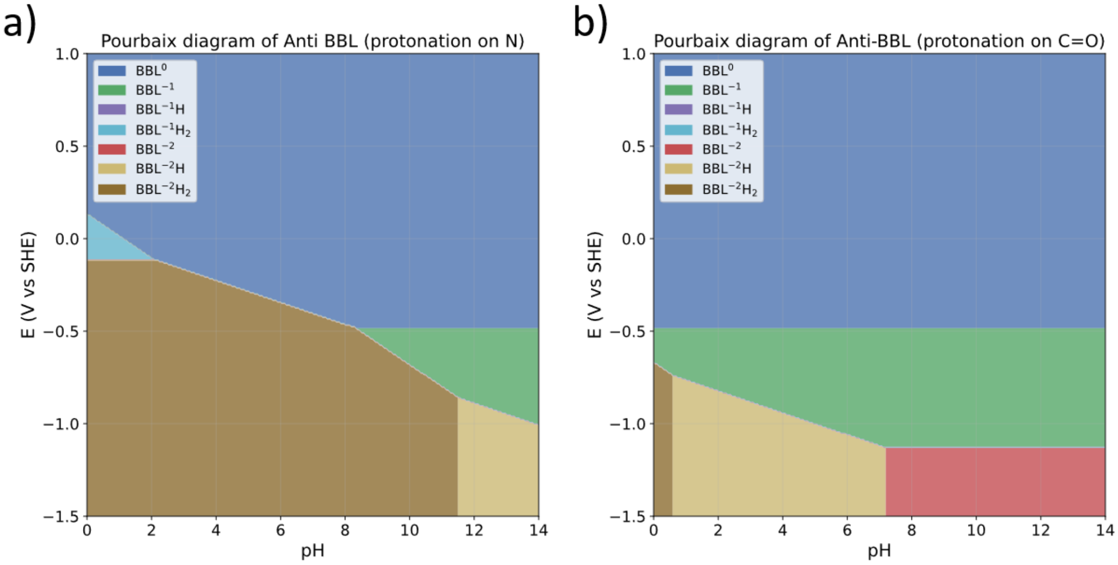

Supplementary Fig. 59 | a,b, Theoretical pourbaix diagram based on computed redox potential and pK_a for the anti-form of perinone assuming a protonation on the imine (**a**) or carbonyl (**b**) site. The syn-form shows similar diagrams.

The DigiElech (Gamry) software[32] was used for the simulation of CV voltammograms based on data from the DFT calculations by considering the scheme of square given in Supplementary Fig. 58. Due to the large differences in pK_a for protonation on C=O compared to N (~10 pK_a units), we only discuss the case of protonation on the imine sites in the following. E and C reactions given in Supplementary Fig. 58 were included for the simulation of CV spectra.

The pH dependence was included by an additional buffer reaction to maintain the concentration in H^+. The equilibrium constant of this last reaction was defined as equal to $[H^+]$[33].

$$\text{Buffer-H} \leftrightarrow \text{Buffer} + H^+$$

Butler-Volmer kinetics were used in the simulation with a charge transfer coefficient of 0.5 and a rate constant k_s of 1 cm^2/s for all electron transfer reactions. Chemical reactions (i.e. protonation reactions) were included with a forward rate constant k_f of 10^9 /s. The diffusion coefficients were set of 10^{-5} cm^2/s for the proton and 2 10^{-6} cm^2/s for all other species. The initial concentration of the perinone molecule (0e/0H) was set to 1 mmol/L. The scan rate was 0.1 V/s for all scan segments of the CV curve. We checked that the main findings discussed below are not sensitive to the actual values of these parameters within a reasonable range.

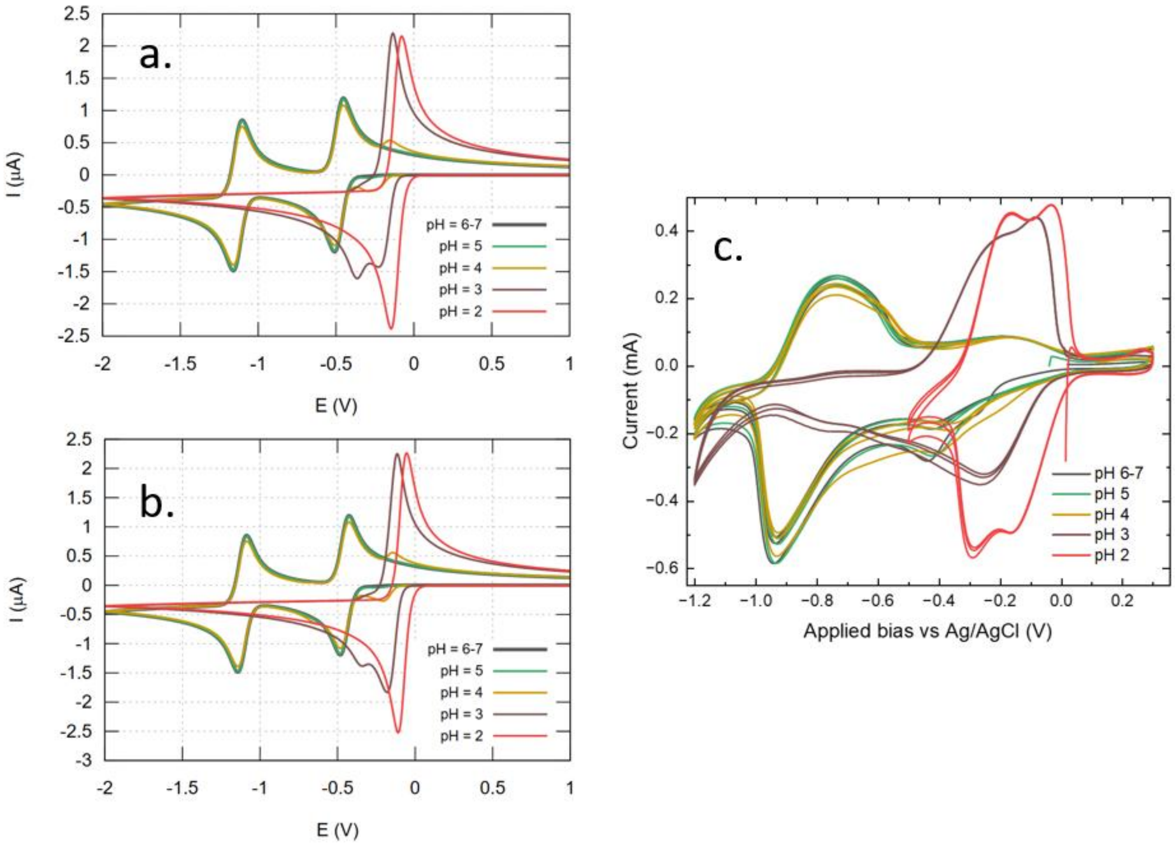

Supplementary Fig. 60 | a-b, Simulated CV voltammogram for anti (**a**) and syn (**b**) BBL as a function of pH when considering a protonation on imidazolium nitrogen. No effect of pH is observed between pH 4-7 while a large shift appears at pH 3 and below. **c**, Experimental CVs measured in 0.1 M NaCl (0.1 V/s) with varying amount of HCl given for comparison.

The resulting simulated CVs for both BBL-forms at several pH values are presented in Supplementary Fig. 60a,b alongside the experimental CV (Supplementary Fig. 60c) obtained in 0.1 M NaCl acidified with HCl. The simulated CVs indicate that the two redox waves at E1 and E2 are constant for small changes in pH (pH 7-4), but merge into a single redox event at more acidic pH (pH ≤ 3). These results are in excellent agreement with the experimental values and literature[34], showing virtually no changes upon decreasing the pH until pH 3 where both redox events occur at lower applied biases and the inter-peak distance decreases drastically. The corresponding surface concentration profiles are given in Supplementary Fig. 61 in the case of protonation on the N site (for anti-configuration of BBL). It confirms that for pH considered in this work, *i.e.*, pH in the range 5-7 (Supplementary Fig. 29), the protonation is negligible with a concentration profile dominated by unprotonated species. Singly and doubly protonated forms of the doubly reduced BBL start to appear at pH = 4 while doubly protonated (BBL^{-2}-2H) is the dominant species upon reduction at pH = 3 and below, and is associated with the disappearance of the E2 peak couple, *i.e.*, reduction follows a 2-electron 2-proton process at this pH.

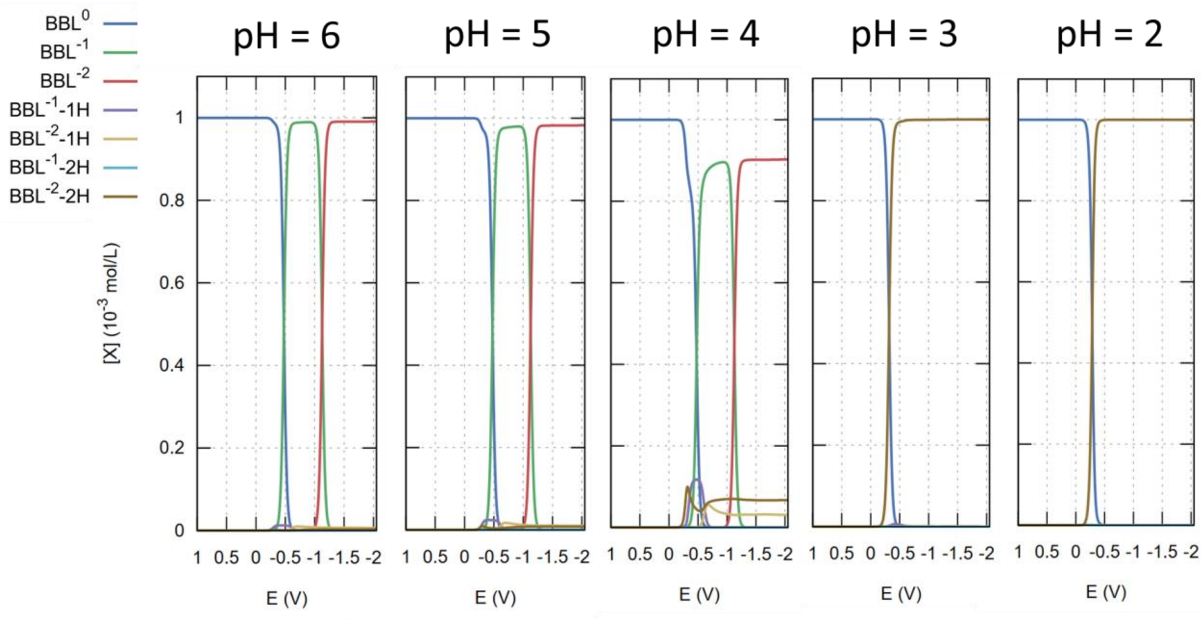

Supplementary Fig. 61 | Surface concentration profiles versus potential for each species for pH range 2-6 in case of Anti BBL protonated on N site.

The redox potentials E1 and E2 were also computed in the presence of ammonium ions to evaluate the possible influence of H-bonding interactions, as described in literature[34]. The resulting CV voltammograms were modelled assuming 1 NH_4^+ for the first reduction (E1) and 2 NH_4^+ for the second reduction (E2). Note that a cluster of 4 water molecules was included in addition to each ammonium ion, preventing proton transfer from the ammonium to the doubly charged BBL that occurs when 'dry' ammonium ions are simulated to interact with BBL. The CV curves are compared to those obtained in absence of compensating cations in Supplementary Fig. 62. The separation between the two main redox waves is computed to be

~0.65 V in the free-ion case, which compares very favorably to the measured values around ~0.5 V for weakly interacting ions such as Na^+. Most importantly, the model predicts a voltage difference drop to ~0.3 V in presence of ammonium groups in a similar way for both the two possible interacting sites (N or C=O), in excellent agreement with the experimental data (with a difference in peak potentials of ~0.25 V, Supplementary Table 6). Thus, on the basis of the combined pH dependence and explicit cation calculations, we can safely conclude that in the current experimental conditions, the interactions between BBL and the protonated cations are dominated by strong H-bonding interactions. These stabilize the doubly-reduced and, to a lesser extent, singly-reduced perinone forms, leading to displacements of the redox waves to lower voltages. We can, however, not exclude that regions of the samples (depending on local pH and water content) undergo protonation of the imine units, as suggested by IR (Supplementary Note 5) simulations and corroborated by water expulsion (Supplementary Note 7) simulations.

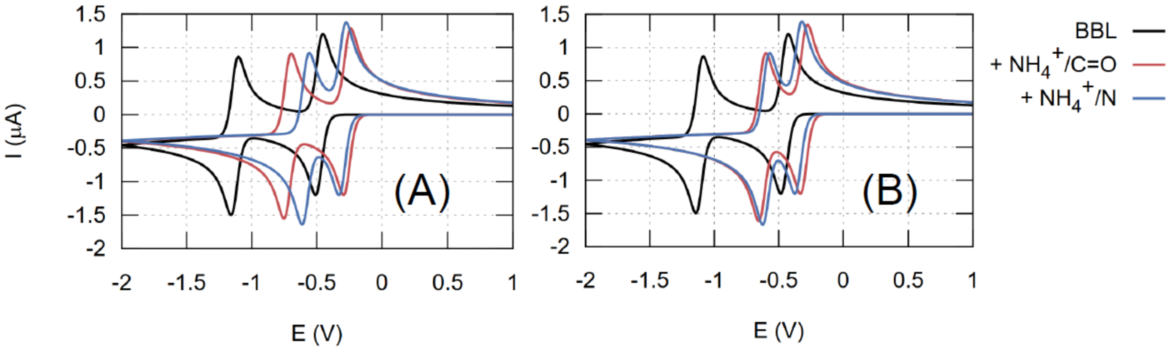

Supplementary Fig. 62 | a,b, Simulated cyclic voltammograms for anti (**a**) and syn (**b**) BBL using the redox potentials given in Supplementary Table 6. CVs were simulated without the presence of ions (black curve) and in presence of (NH_4^+ + 4 H_2O) cluster in close contact with carbonyl (red curve) or imidazolium nitrogen (blue curve).

Supplementary Table 6 | Computed redox potentials (E1, E2) for Anti and Syn BBL with and without the presence of ammonium ions. The inter-peak distance |ΔE| is given in the last column. Experimental data *vs* Ag/AgCl are given for comparison.

		E1 (V)	E2 (V)	|ΔE| (V)
No ion	Anti	-0.481	-1.131	0.650
	Syn	-0.454	-1.115	0.661
NH_4^+ + 4 H_2O facing N	Anti	-0.301	-0.586	0.286
	Syn	-0.346	-0.598	0.252
NH_4^+ + 4 H_2O facing C=O	Anti	-0.266	-0.725	0.459
	Syn	-0.303	-0.628	0.325
Experimental BBL + ….	NaCl	-0.393	-0.880	0.487
	NH_4Cl	-0.333	-0.611	0.278
	MMACl	-0.372	-0.759	0.387
	DMACl	-0.393	-0.740	0.347
	TriMACl	-0.351	-0.687	0.336
	TMACl	-0.351	-0.886	0.535
	HCl	-0.078	-0.190	0.112

Supplementary Note 7: MD simulations of fully doped protonated samples

To assess how protonation of the imidazole nitrogens affects the swelling of 200% doped (aka 2 electrons per BBL monomer) samples, we first determined the supramolecular organization of charged BBL chains with all imidazole nitrogens protonated using the same methodology as for neat BBL. As expected, the three-dimensional structure of neat BBL is not strongly altered by protonation or doping; the interlayer distance is now 7.94 Å compared to 7.64 Å for the unprotonated case.

We then constructed a small crystallite of protonated BBL comprising four layers of ten π-stacked chains, with an interlayer spacing of 10.2 Å, i.e., the average value observed in our doping simulations with NH_4Cl as the electrolyte. This crystallite was immersed in a large simulation box, and a small number of water molecules were manually inserted into the interlayer regions. A 1 ns molecular-dynamics equilibration was run with the BBL chains held fixed to equilibrate the interlayer water content, followed by a 2 ns MD production simulation run with all atoms relaxed.

After ~2 ns, the protonated BBL layers approach each other and are predominantly separated by a single water monolayer, leading to an average ~35% decrease in the number of interlayer water molecules (Supplementary Fig. 63). Water molecules in this monolayer adopt a specific, robust hydrogen-bonding layout: both H atoms donate H-bonds to carbonyl oxygens of one layer, while the water O atom accepts an H-bond from a protonated imidazolium N–H on the adjacent layer (Supplementary Fig. 64). In addition, while the π–π stacking distance was found to expand at high doping levels in our initial doping MD simulations, inclusion of imidazole protonation prevents this expansion: the π–π spacing remains close to ~3.53 Å, comparable to the undoped value. We consider that the formation of this ordered phase involving water monolayers associated with a preserved π–π stacking upon protonation at high doping levels may contribute to the larger deswelling process observed experimentally when using protic cations.

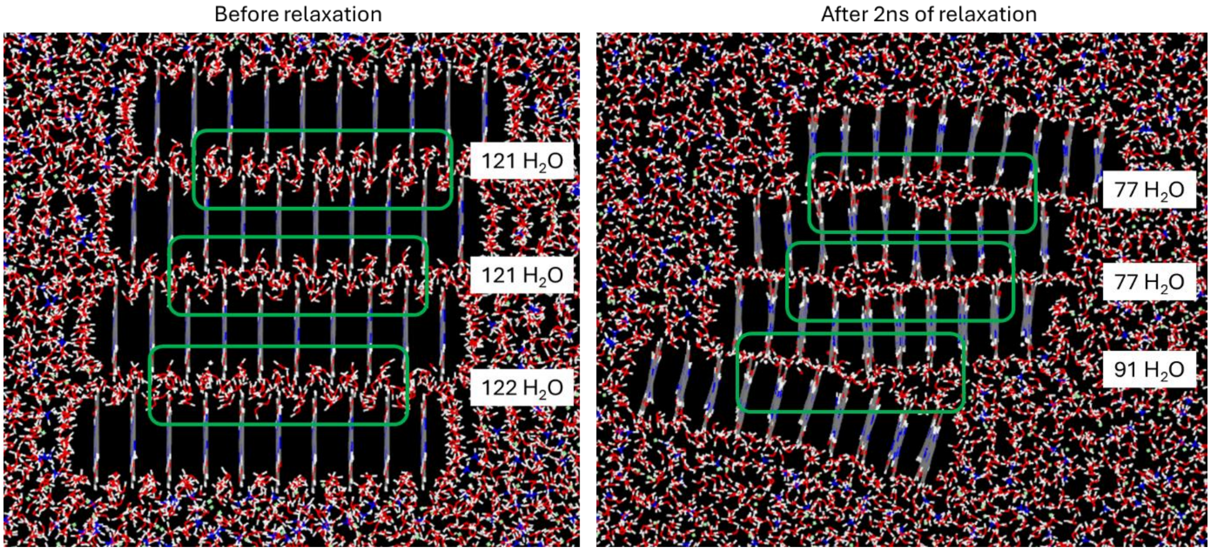

Supplementary Fig. 63 | Simulated protonated BBL:ion system at 200% doping, shown before BBL-layer relaxation (left) and after a 2 ns MD simulation (right). The initial interlayer spacing is set to 10.2 Å (left). The numbers of water molecules inserted within the interlayer galleries (highlighted in green) are indicated on each panel.

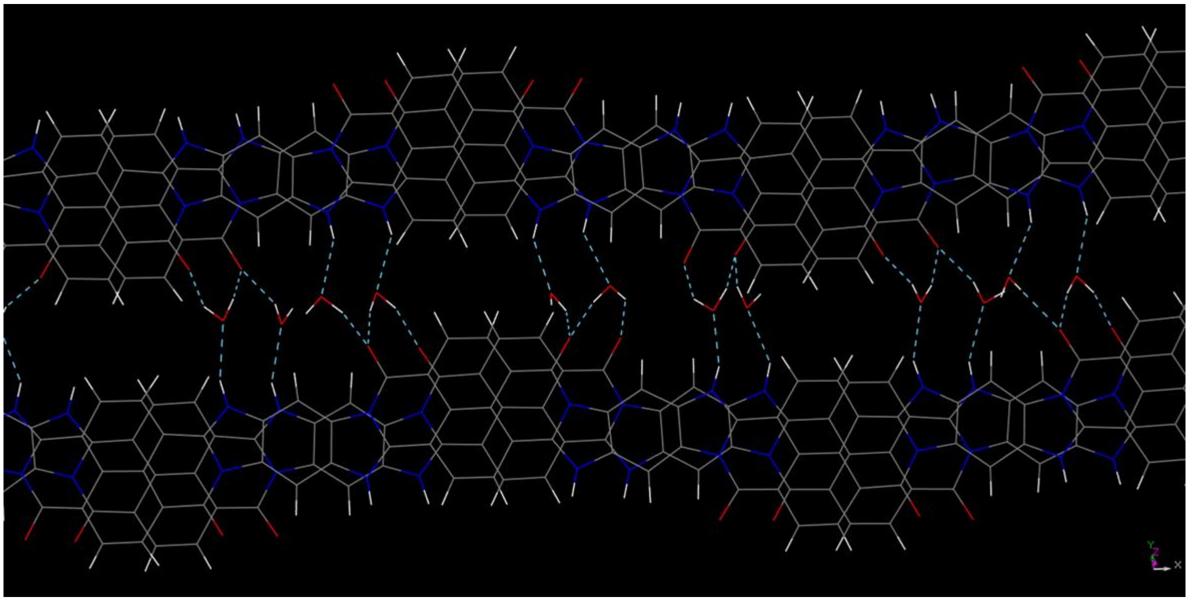

Supplementary Fig. 64 | Enlarged views of the interlayer regions in protonated 200% doped BBL:ions systems. The hydrogen-bonding motif adopted by interlayer water is shown with dashed green lines; the example illustrates a single water monolayer confined between adjacent BBL layers.

Supplementary Note 8: Periodic DFT calculations

a) Atomic charges.

The atomic charges were computed at the periodical DFT level using the VASP package. We considered a BBL dimer (BBL_2) in its "Syn" conformation as the repeated unit along the *a* direction of the unitcell. (Supplementary Fig. 65) The *b* and *c* directions were fixed to 20 Å and 15 Å, respectively to prevent interactions between BBL chains. PBE functional was used at the Projected Augmented-Wave (PAW) scheme with a mesh cutoff of 500 eV and a k-point grid of (2 × 1 × 1). We considered charge states of 0 (neutral), -1, -2, -3 and -4 for the dimer. The BBL chain at each charge state was allowed to fully relax with negligible differences found along the *a* axis (24.37 Å-24.40 Å). The atomic charges were then computed from the converged charge density using the DDEC6 partition scheme. The same approach was also repeated using hybrid HSE06 functional without showing significant differences in the atomic charge distribution (Supplementary Fig. 66).

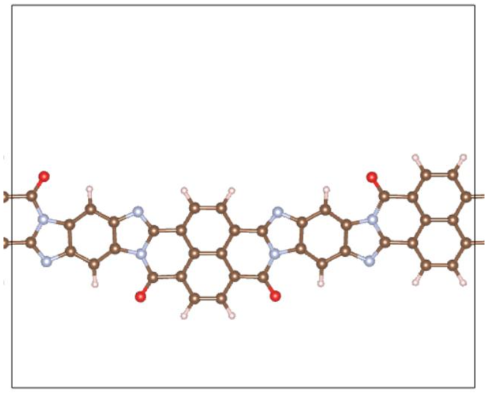

Supplementary Fig. 65 | Top view of the unit cell used at the DFT level.

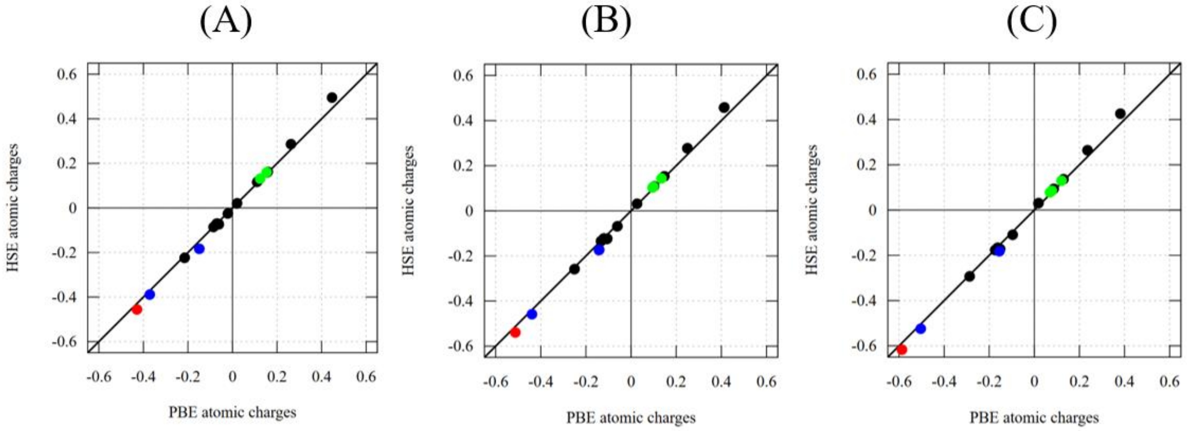

Supplementary Fig. 66 | a-c, Comparison between atomic charges obtained at the PBE (x axis) and HSE (y axis) level for a BBL dimer of total charge equal to 0 (**a**), -2 (**b**) and -4 (**c**). The color of the points refers to the different atomic species: oxygen (red), carbon (black), nitrogen (blue) and hydrogen (green).

b) Band structure calculations

As a first assessment of the changes in conductivity with doping and protonation, we have computed the electronic band structure of an infinite BBL chain (taking a dimer, aka 2 BBL monomers or BBL_2, as repeating unit) at the PBE level (see above) in neutral, 50% and 100 % (un)protonated forms, see Supplementary Fig. 67. At 50% doping (q=-2 in BBL_2), the conduction band of the pristine chain is half-filled, as expected at this level of theory (the opening of a Mott gap could only possibly occur when considering the interplay between electron correlation and disorder effects). The electronic band structure of the corresponding protonated form is very similar to the unprotonated chain at that level of doping. At 100% doping and in absence of protonation (q=-4 in BBL_2), the conduction band of the pristine chain is, again as expected, fully filled with excess electrons and there is a high density of states at Fermi energy (metallic-like behavior). In contrast, at the same level of doping, there is a massive opening of a bandgap (getting closer to that of the pristine, unprotonated, polymer) in the protonated form that shows no states close to the Fermi energy. These calculations indicate the system switches from a metallic-like to a semiconducting/insulating state upon protonation.

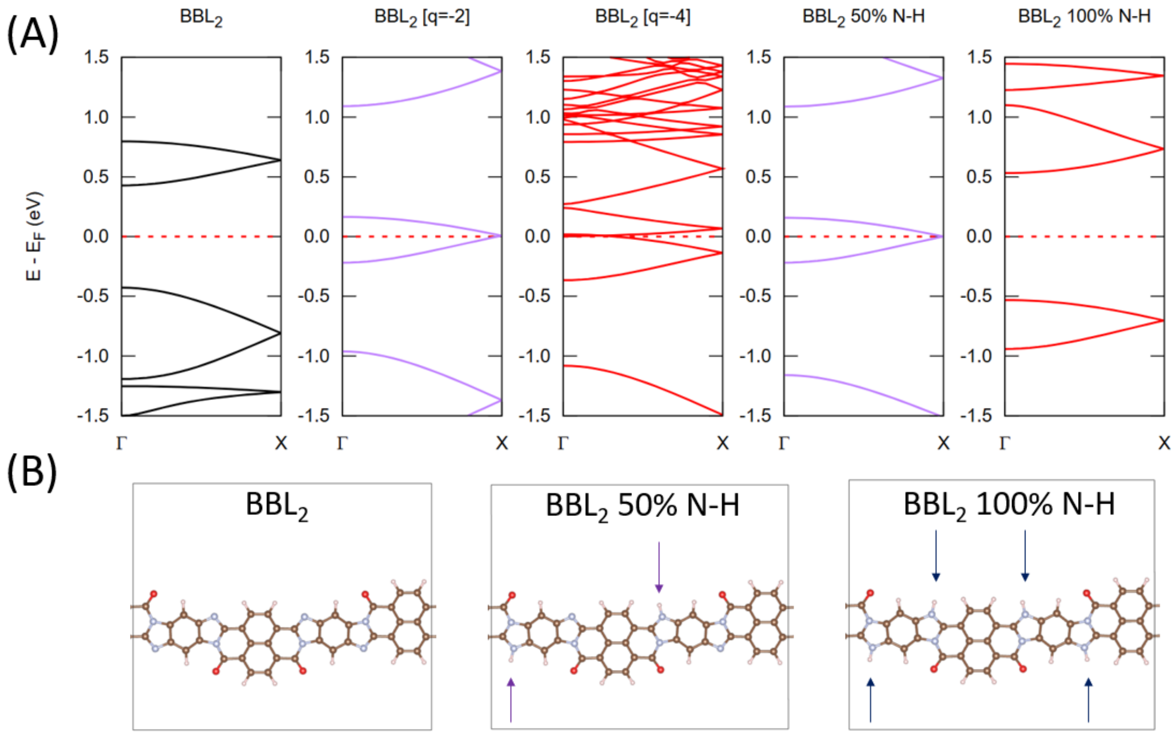

Supplementary Fig. 67 | a, Computed band structure along the molecular axis (Γ-X path) for a periodic BBL dimer in its neutral state (black) and for a 50% (purple) and 100% (blue) protonated chains. Band structures of -2 and -4 charged dimers (i.e. one and two charges per BBL monomert, respectively) are given for comparison. **b**, Top view of the optimized structures. We considered protonation on the nitrogen atoms (see arrows) as our simulation of pK_a suggest that this site is the most favorable (see Supplementary Note 5 and 6).

Supplementary Note 9: Optical response of BBL upon doping in NaCl and NH_4Cl

We perform operando spectroscopic ellipsometry at a 70-degree angle on a 20 nm BBL film on a gold-coated (Au/Cr) silicon substrate submerged in 0.1 M NH_4Cl or NaCl while applying up to -1.1 V *vs* Ag/AgCl. An excellent fit to the ellipsometry data is obtained with a collection of anisotropic Tauc-Lorentz oscillators including an IR pole and assuming a thickness expansion as measured with EC-AFM (Fig. 1d). The resulting (in-plane) complex refractive index data are shown in Supplementary Fig. 68a,b. The imaginary part of the refractive indices (*k*) closely match the spectro-electrochemical absorption of BBL at these biases,[5] confirming the quality of the ellipsometry and fitting.

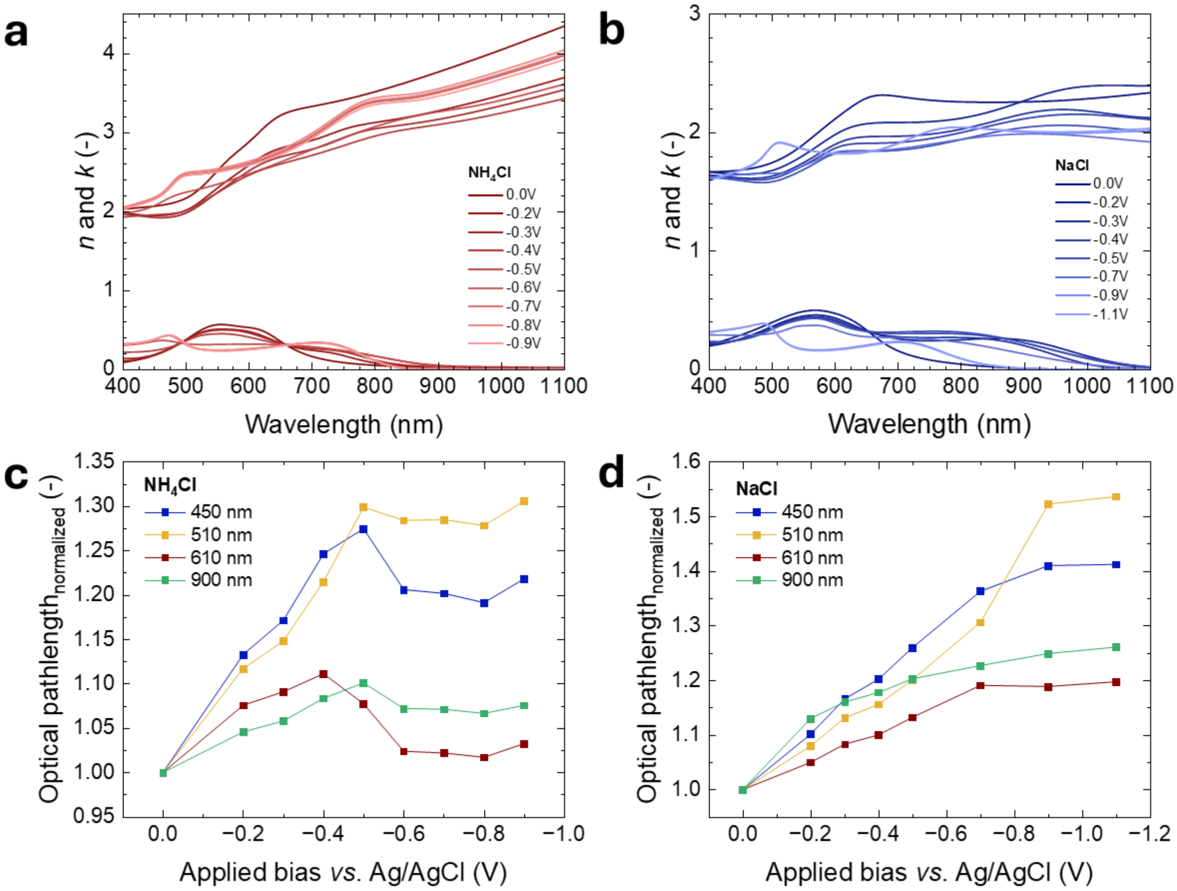

Supplementary Fig. 68 | a,b, Complex refractive index data of BBL doped in 0.1 M NH_4Cl or b) 0.1 M NaCl under applied bias. **c,d**, The normalized optical pathlength calculated as a function of bias for 450 nm, 510 nm, 610 nm, and 900 nm for c) 0.1 M NH4Cl and d) 0.1 M NaCl. Normalization is performed against the optical pathlength at 0.0 V.

By combining the refractive index and the film thickness at a given bias, the optical pathlength can be calculated. We perform these calculations for all employed biases and present several wavelengths in Supplementary Fig. 68c,d. Markedly, the optical pathlength for NH4Cl exhibits an initial increase followed by a distinct decrease for several wavelengths. For 450 nm and 610 nm, the decrease in pathlength can be up to 10% of the pristine pathlength at 0.0V. In the case of NaCl, however, the optical pathlength shows a constant increase and a relative flattening at higher bias (> -0.8 V). We note that these optical pathlength changes are similar to what has been recently reported for PEDOT:PSS applied in metasurfaces[35].

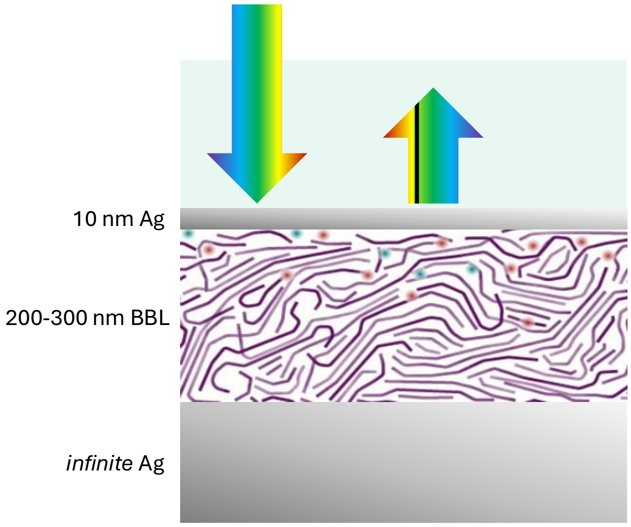

Supplementary Fig. 69 | A simple Fabry-Perot cavity consisting of a BBL film (200-300 nm) sandwiched between a semi-transparent mirror (10 nm Ag) and an infinitely thick mirror (Ag). Depending on the optical pathlength, there can be constructive and destructive interference within the cavity which removes a narrow band in the reflectance spectrum. This layout was used in the optical model based on the transfer-matrix formalism to predict bias-dependent reflectance.

This unique optical pathlength response to bias, enabled by the thickness and refractive index response of BBL gated in NH_4Cl, can be leveraged in applications for metasurfaces. As a proof of concept, we model a Fabry-Perot cavity employing BBL using the transfer-matrix formalism[36] (Supplementary Fig. 69) and predict the reflectance as function of applied bias. We regard both the changes in thickness due to (de-)swelling and complex refractive index for gating with 0.1 M NH_4Cl and 0.1 M NaCl, and the resulting spectra are displayed in Supplementary Fig. 70 and 71, respectively.

In Supplementary Fig. 68, we observe that the spectra of a film of 280 nm (Supplementary Fig. 70a) and 300 nm (Supplementary Fig. 70b) gated in NH_4Cl shows distinct dips in the reflectance. The exact wavelength and depth of this feature is strongly dependent on the bias applied to the film and, as a result, a specific wavelength shows a strongly bias dependent reflectance. Due to the concerted deswelling and change in refractive index, the reflectance dip for NH_4Cl shows an initially gradual red-shift that is rapidly followed by significant blue-shift. In Supplementary Fig 70c,d, we plot the reflectance for selected wavelengths as function of bias for both film thicknesses. We find that the reflectance shows a very sharp dip as a function of bias, amounting to a loss of up to 75% reflectance intensity in a ΔV of only -0.2 V. Depending on the wavelength that is focused on, the same bias step can result in a strong increase in reflectance, a decrease in reflectance, or even a near-zero reflectance, as can be seen in Supplementary Fig. 70d. Thereby this proof of concept shows that BBL in 0.1M NH_4Cl is a potentially interesting optical element, owing to its refractive index response and deswelling.

For doping a BBL film of 200 nm or 280 nm with NaCl, however, the Fabry-Perot cavity shows a reflectance dip without gradual changes but rather two distinct positions (Supplementary Fig. 71a,b, respectively). This is also apparent in the reflectance of a single wavelength as a function of bias (Supplementary Fig. 71c,d) where we observe that the reflectance gives either a semi-continuous high or low value when a bias is

applied. Clearly the unique thickness and refractive index response of BBL gated in NH_4Cl and NaCl allow for distinctly different and applicable optical responses in a Fabry-Perot cavity.

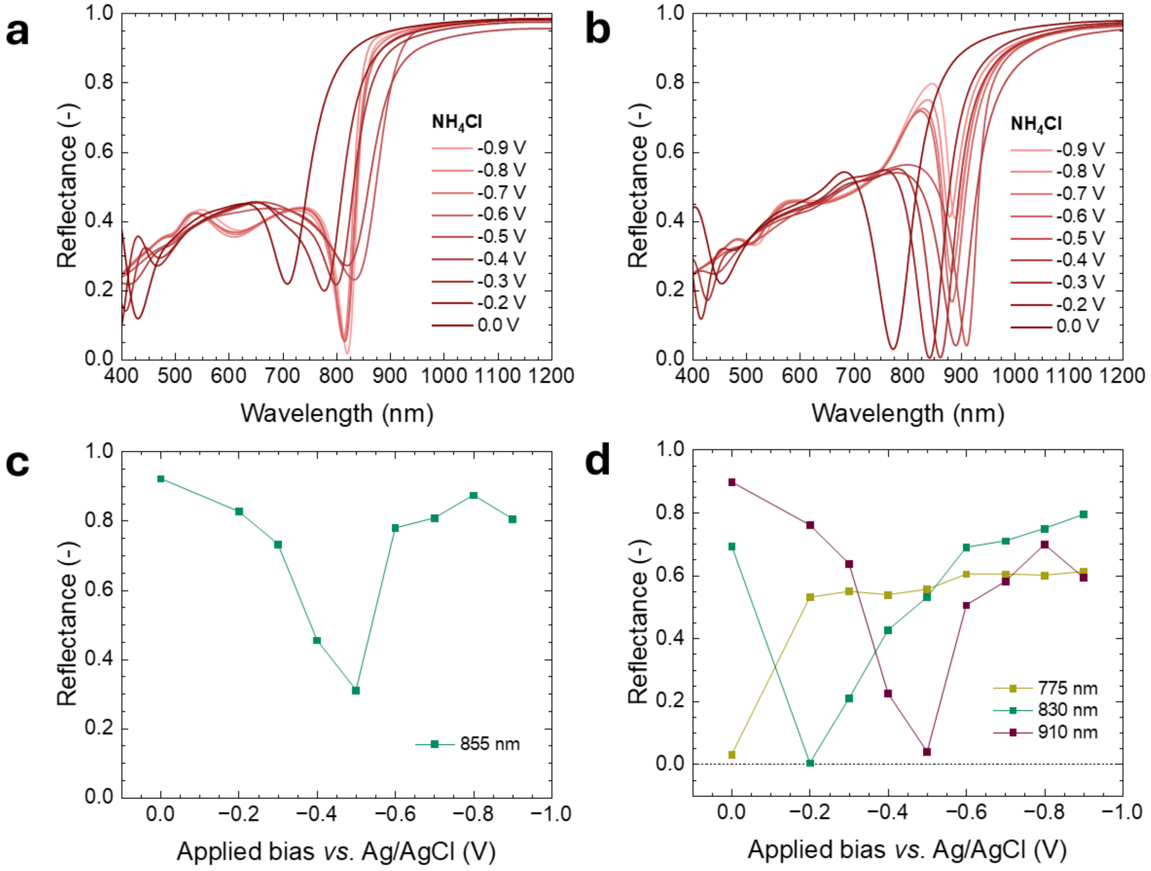

Supplementary Fig. 70 | a,b, The calculated reflectance for a Fabry-Perot cavity such as in Supplementary Fig. 69 with a BBL film of a) 280 nm and b) 300 nm gated with 0.1 M NH_4Cl as function of applied bias. **c,d**, Reflectance as function of applied bias for selected wavelengths for a Fabry-Perot cavity including c) a 280 nm BBL film or d) a 300 nm BBL film.

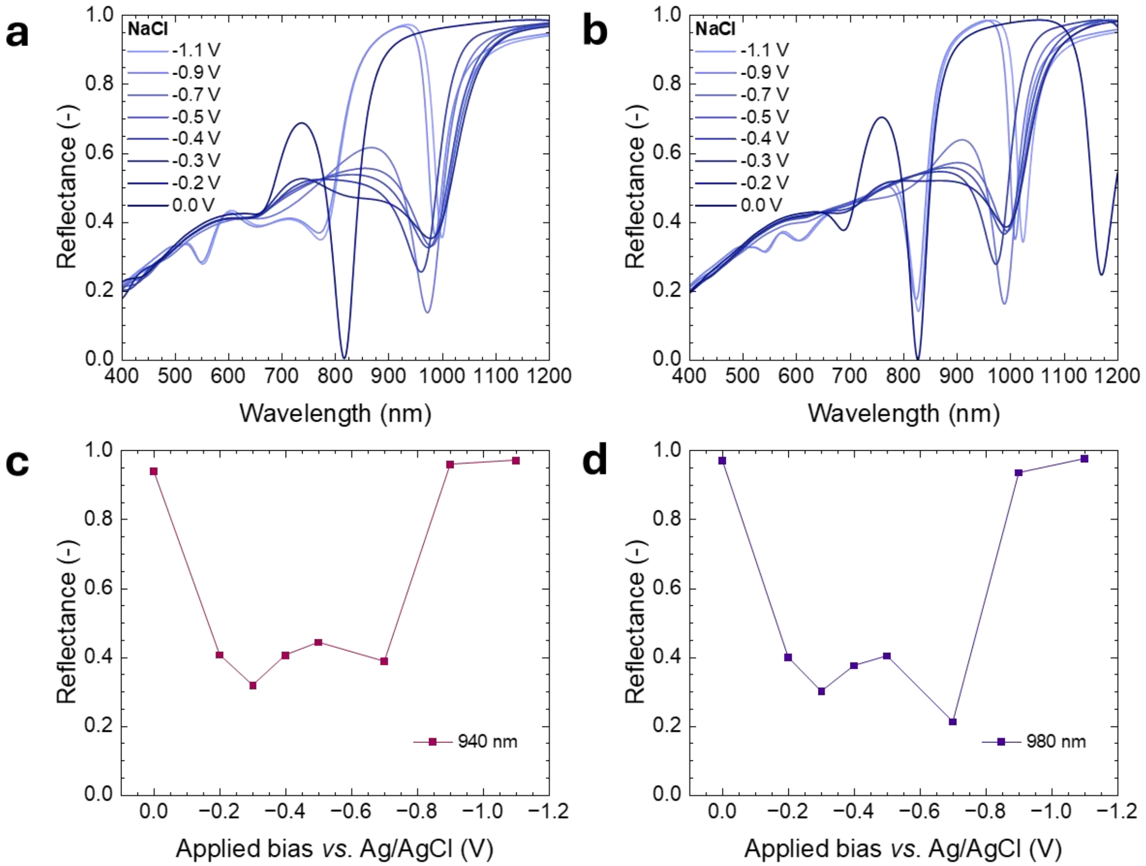

Supplementary Fig. 71 | a,b, The calculated reflectance for a Fabry-Perot cavity such as in Supplementary Fig. 69 with a BBL film of a) 200 nm and b) 280 nm gated with 0.1 M NaCl as function of applied bias. **c,d**, Reflectance as function of applied bias for selected wavelengths for a Fabry-Perot cavity including c) a 200 nm BBL film or d) a 280 nm BBL film.

References

1. Harikesh, P. C. et al. Ion-tunable antiambipolarity in mixed ion–electron conducting polymers enables biorealistic organic electrochemical neurons. *Nat Mater.* **22**, 242-248 (2023).
2. Bates, R. G. & Pinching, G. D. Acidic dissociation constant of ammonium ion at 0 to 50 C, and the base strength of ammonia. *J. Res. Natl. Bur.* **42**, 419-430 (1949).
3. Everett, D. H. & Wynne-Jones, W. F. K. The dissociation constants of the methylammonium ions and the basic strengths of the methylamines in water. *Proc. R. Soc. Lond. A* **177** 499–516 (1941).
4. Guo, J. et al. Hydration of a side-chain-free n-type semiconducting ladder polymer driven by electrochemical doping. *J Am Chem Soc* **145**, 1866–1876 (2023).
5. Wu, H. Y. et al. Influence of molecular weight on the organic electrochemical transistor performance of ladder-type conjugated polymers. *Advanced Materials* **34**, 2106235 (2022).
6. Märker, K. et al. Welcoming natural isotopic abundance in solid-state NMR: probing π-stacking and supramolecular structure of organic nanoassemblies using DNP. *Chem. Sci.* **8**, 974-987 (2017).
7. Schuettfort, T. et al. Microstructure of polycrystalline PBTTT films: domain mapping and structure formation. *ACS Nano* **6**, 1849-1864 (2012).
8. Schmidt-Rohr, K. & Spiess, H. W. *Multidimensional solid-state NMR and polymers* (Elsevier Science, 2012).
9. Brauckmann, J. O., Verhoef, R., Schotman, A. H. M. & Kentgens, A.P.M. Solid-state nuclear magnetic resonance characterization of residual 23Na in aramid fibers. *J. Phys. Chem. C* **123**, 14439-14448 (2019).
10. Lyu, D. et al. Operando NMR electrochemical gating studies of ion dynamics in PEDOT:PSS. *Nat. Mater.* **22**, 746-753 (2023).
11. Ye, G., Hayden, C. A. & Goward, G. R. Proton dynamics of nafion and nafion/SiO2 composites by solid state NMR and pulse field gradient NMR. *Macromolecules* **40**, 1529-1537 (2007).
12. Cherry, B. R., Fujimoto, C. H., Cornelius, C. J. & Alam, T. M. Investigation of domain size in polymer membranes using double-quantum-filtered spin diffusion magic angle spinning NMR. *Macromolecules* **35**, 1201-1206 (2005).
13. Ye, G., Janzen, N. & Goward, G. R. Solid-state NMR study of two classic proton conducting polymers: nafion and sulfonated poly(ether ether ketone)s. *Macromolecules* **39**, 3283-3290 (2006).
14. Fung, B. M. Orientation of water in striated frog muscle. *Science* **190**, 800-802 (1975).
15. Ooms, K. J., Vega, A. J., Polenova, T., Cannella, M., & Marcolongo, M. Double and zero quantum filtered 2H NMR analysis of D2O in intervertebral disc tissue. *Journal of Magnetic Resonance*, **258**, 6-11 (2015).
16. Shinar, H. et al. Mapping the fiber orientation in articular cartilage at rest and under pressure studied by 2H double quantum filtered MRI. *Magn. Reson. Med.* 48, 322-330 (2002).
17. Sharf, Y., Eliav, U., Shinar, H. & Navon, G. Detection of anisotropy in cartilage using 2h double-quantum-filtered NMR-spectroscopy. *J. Magn. Reson. Ser. B* **107**, 60-67 (1995).
18. Sun, C. & Boutis, G. S. Investigation of the dynamical properties of water in elastin by deuterium Double Quantum Filtered NMR. *J. Magn. Reson.* **205**, 86-92 (2010).
19. Vanderschee, C. R. & Ooms, K. J. Investigating water interactions with collagen using 2h multiple quantum filtered NMR spectroscopy to provide insights into the source of Double Quantum Filtered signal in tissue. *J. Phys. Chem. B* **118**, 3491-3497 (2014).

20. Woudstra, J. M. & Ooms, K. J. Investigating the water in hydrated sPEEK membranes using multiple quantum filtered 2H NMR spectroscopy. *J. Phys. Chem. B* **116**, 14724-14730 (2012).
21. John, M. & Rüttger, F. Multinuclear residual quadrupolar couplings for structure and assignment. *ChemPhysChem* **25**, e202400068 (2024).
22. Schäfer, H., Mädler, B. & Sternin, E. Determination of orientational order parameters from ^{2}H NMR spectra of magnetically partially oriented lipid bilayers. *Biophysical Journal* **74**, 1007-1014 (1998).
23. Butler, L. G. & Keiter, E. A. Interpretation of electric field gradients at deuterium as measured by solid-state NMR spectroscopy. *Journal of Coordination Chemistry 32*, 121–134 (1994).
24. Flagg, L. Q. et al. P-type electrochemical doping can occur by cation expulsion in a high-performing polymer for organic electrochemical transistors. *ACS Materials Lett.* **2**, 254−260 (2020).
25. Price, W. S., Kuchel, P. W. & Cornell, B. A. A ^{35}Cl and ^{37}Cl NMR study of chloride binding to the erythrocyte anion transport protein. *Biophysical chemistry* **40**, 329-337 (1991).
26. Frisch, M. J. et al. Gaussian 16 revision C.01, *Gaussian Inc. Wallingford CT* (2016).
27. Alecu, I. M., Zheng, J., Zhao, Y., Truhlar, D. G. Computational thermochemistry: scale factor databases and scale factors for vibrational frequencies obtained from electronic model chemistries. *J. Chem. Theory Comput.* **6**, 2872-2887 (2010).
28. Łapkowski, M. Perinone-new life of an old molecule. *Materials* **14**, 6880 (2021).
29. Wang, M. et al. Poly(benzimidazobenzophenanthroline)-ladder-type two-dimensional conjugated covalent organic framework for fast proton storage. *Angew. Chemie Int. Ed.* **62**, e202310937 (2023).
30. Trasatti, S. The absolute electrode potential: an explanatory note (recommendations 1986). *Pure & Appl. Chem. 58*, 955-966 (1986).
31. Ribeiro Dutra, F. & Custodio, R. Comparative assessment of the direct and isodesmic methods for pKa calculation of monocarboxylic acids using density functional theory. *Comput. Theor. Chem.* **1237**, 114629 (2024).
32. Rudolph, M. DigiElch 8.0; Available on http://www.elchsoft.com.
33. Wiberg, C., Busch, M., Evenäs, L. & Ahlberg, E. The electrochemical response of core-functionalized naphthalene diimides (NDI) – a combined computational and experimental investigation. *Electrochim. Acta* **367**, 137480 (2021).
34. Gupta, N. & Henry, L. Hydrogen-bonding and protonation effects in electrochemistry of quinones in aprotic solvents. *J. Am. Chem. Soc.* **119**, 6384-6391 (1997).
35. Doshi, S. et al. Electrochemically mutable soft metasurfaces. *Nat. Mater.* **24**, 205-211 (2024).
36. Burkhard, G. F., Hoke, E. T. & McGehee, M. D. Accounting for interference, scattering, and electrode absorption to make accurate internal quantum efficiency measurements in organic and other thin solar cells. *Adv. Mater.* **22**, 3293 (2010).
